# Supplementary material for: Cellular and molecular landscapes of human tendons across the lifespan revealed by spatial and single-cell transcriptomics
Source: Cell Rep. 2026 Mar 20;45(4):117085. doi: 10.1016/j.celrep.2026.117085 (PMC13328346; doi:10.1016/j.celrep.2026.117085)
Supplement: Document S1. Figures S1–S15 and Data S1 and S2 [file mmc1.pdf]

Cell Reports, Volume *45*

Supplemental information

Cellular and molecular landscapes of human tendons across the lifespan revealed by spatial and single-cell transcriptomics

Alina Kurjan, Jolet Y. Mimpen, Lorenzo Ramos-Mucci, Ali C. Aksu, Carla J. Cohen, Mate Naszai, Christopher D. Buckley, Adam P. Cribbs, Mathew J. Baldwin, and Sarah J.B. Snelling

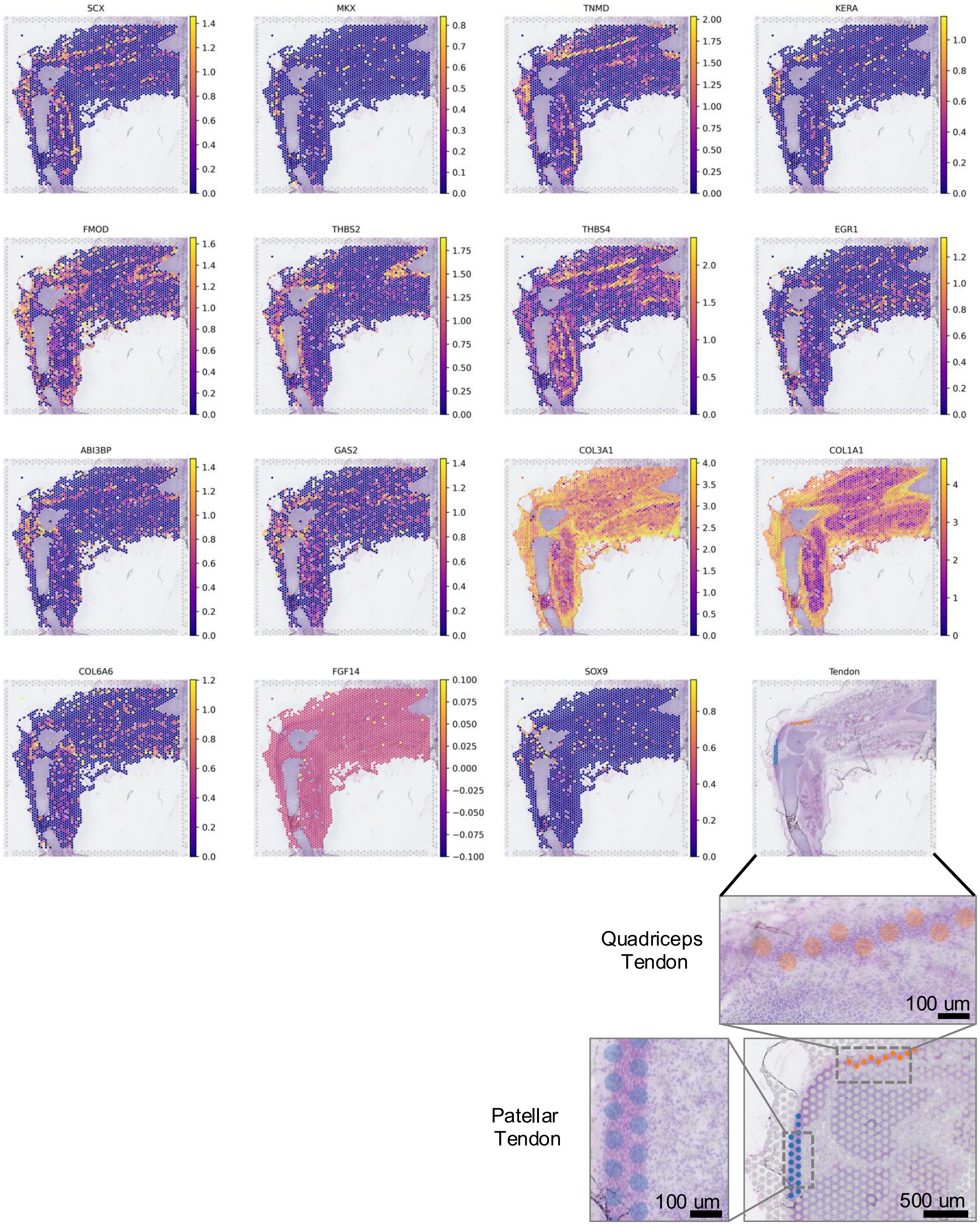
SCX
MKX
TNMD
KERA
FMOD
THBS2
THBS4
EGR1
ABI3BP
GAS2
COL3A1
COL1A1
COL6A6
FGF14
SOX9
Tendon
Quadriceps Tendon
100 um
Patellar Tendon
100 um
500 um

Figure S1. Tendon cell marker expression and tissue demarcation in a whole 8 post-conception week embryonic human leg.

Visium scatterplots show normalised gene expression values for key tendon cell markers, revealing their spatial distribution within the developing leg. The ‘Tendon’ panel shows manually annotated tendon regions identified from H&E-stained tissue sections. These annotated regions were used to train the random forest classifier, enabling the identification of tendon cell types in embryonic whole limb single-cell data based on this spatial RNA-sequencing data. The inset shows the annotated knee region of the embryonic limb. Orange spots correspond to the quadriceps tendon, while blue spots demarcate the patellar tendon.

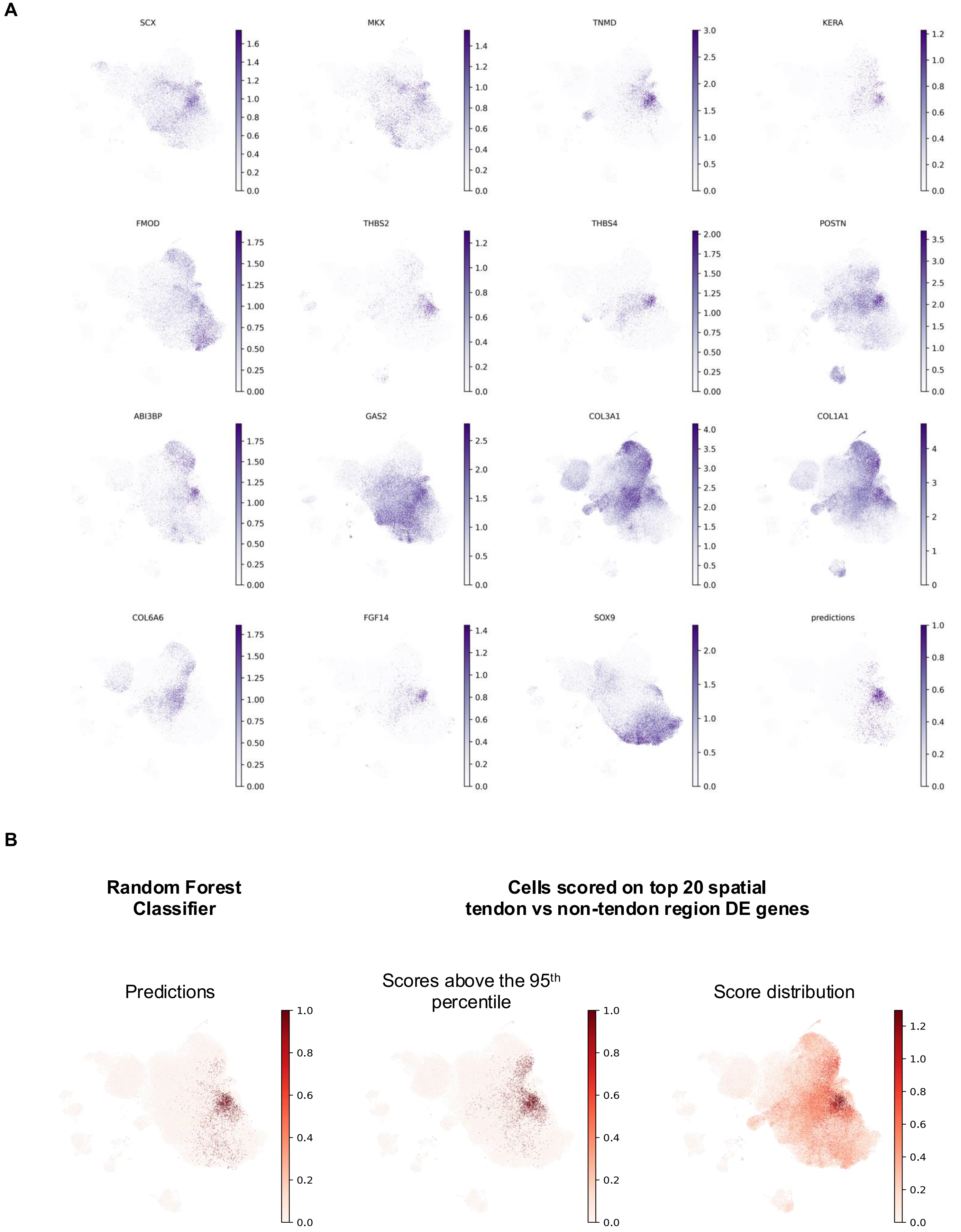
A
SCX
MKX
TNMD
KERA
FMOD
THBS2
THBS4
POSTN
ABI3BP
GAS2
COL3A1
COL1A1
COL6A6
FGF14
SOX9
predictions
B
Random Forest Classifier
Cells scored on top 20 spatial tendon vs non-tendon region DE genes
Predictions
Scores above the 95th percentile
Score distribution

Figure S2. Expression of early tendon markers in human embryonic single-cell RNA-sequencing data from fore- and hindlimbs of 11 human embryonic donors.

(A) UMAPs show normalised gene expression values for early tendon markers relevant to early tendon development. The 'predictions' panel shows the likely tendon cell types identified using a random forest classifier, which was trained on spatial RNA-sequencing data from 8 post-conception week embryonic hindlimbs. (B) UMAPs show comparison of random forest classifier vs gene scoring methods for tendon cell identification within whole limb embryonic scRNA-seq data. Random forest classifier was trained on the spatial gene expression profiles of tendon and non-tendon regions. The Predictions UMAP plot (left) highlights all cells identified as tendons by this classifier. Middle and right UMAP plots show tendon cell identification by cell scoring on the top 20 differentially expressed genes between spatial tendon and non-tendon regions. Right plot shows the scores. Middle plot shows the cells with scores above the 95th percentile.

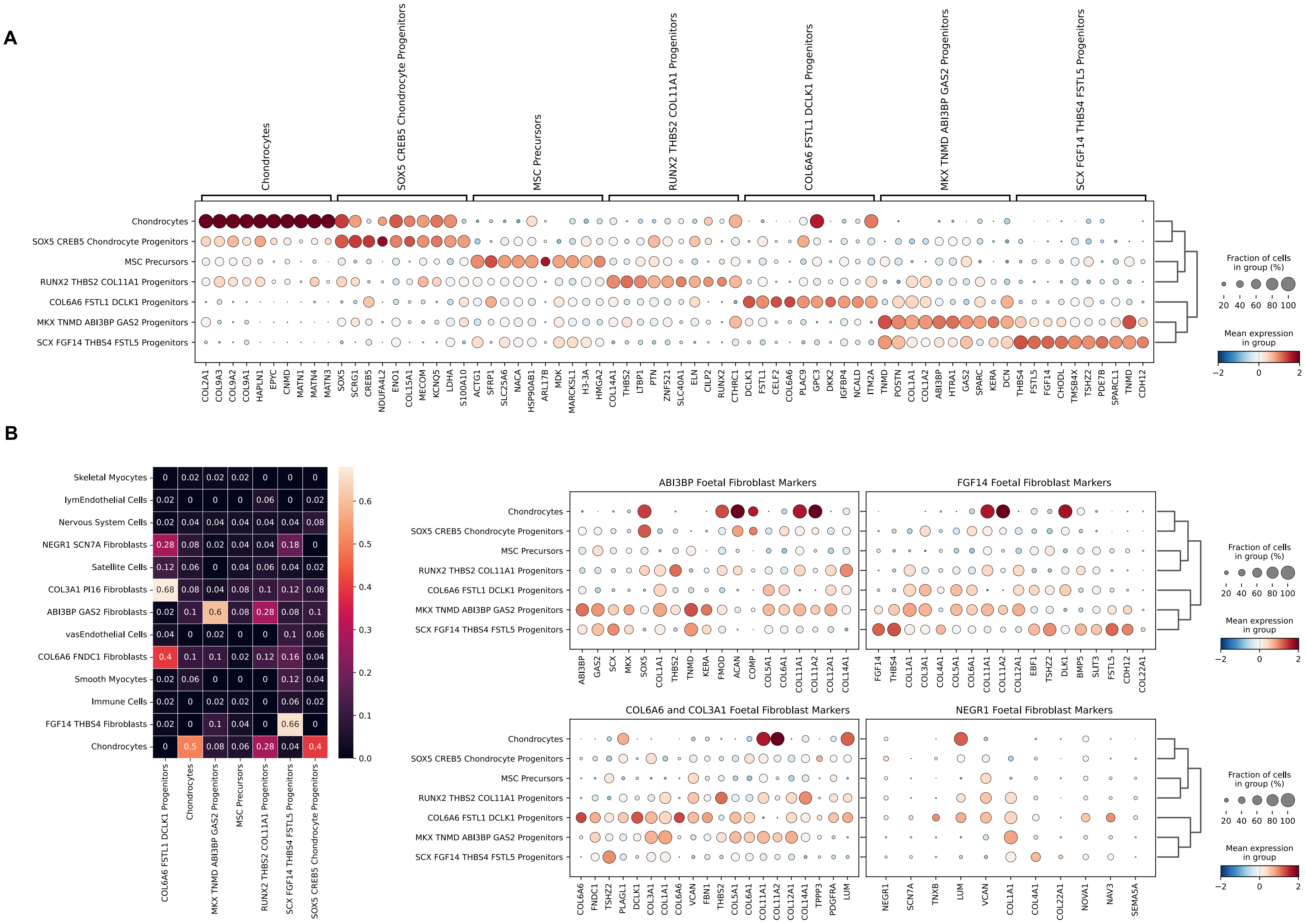
A
Chondrocytes
SOX5 CREB5 Chondrocyte Progenitors
MSC Precursors
RUNX2 THBS2 COL11A1 Progenitors
COL6A6 FSTL1 DCLK1 Progenitors
MKX TNMD ABI3BP GAS2 Progenitors
SCX FGF14 THBS4 FSTL5 Progenitors
Fraction of cells in group (%)
20 40 60 80 100
Mean expression in group
−2 0 2
B
Skeletal Myocytes
lymEndothelial Cells
Nervous System Cells
NEGR1 SCN7A Fibroblasts
Satellite Cells
COL3A1 PI16 Fibroblasts
ABI3BP GAS2 Fibroblasts
vasEndothelial Cells
COL6A6 FNDC1 Fibroblasts
Smooth Myocytes
Immune Cells
FGF14 THBS4 Fibroblasts
Chondrocytes
ABI3BP Foetal Fibroblast Markers
FGF14 Foetal Fibroblast Markers
COL6A6 and COL3A1 Foetal Fibroblast Markers
NEGR1 Foetal Fibroblast Markers

Figure S3. Differentially expressed genes in human 6.5-9.3 post-conception week (pcw) embryonic tendon cell types and comparison with foetal tendon cell signatures.

(A) Dotplot shows clustered log1pPF-normalised and scaled expression of top 10 differentially expressed genes for each embryonic tendon cell type. (B) (Left) Matrixplot shows proportional overlap of the top 50 differentially expressed genes identified in cell types from 12 pcw foetal tendons against an array of embryonic cell types. Each cell in the matrix represents the percentage of overlapping DEGs, calculated as the number of shared genes divided by the total number of genes in the embryonic reference set, multiplied by 100 to express the value as a percentage. (Right) Dotplots show clustered log1pPF-normalised and scaled expression of foetal tendon fibroblast markers in embryonic cell types.

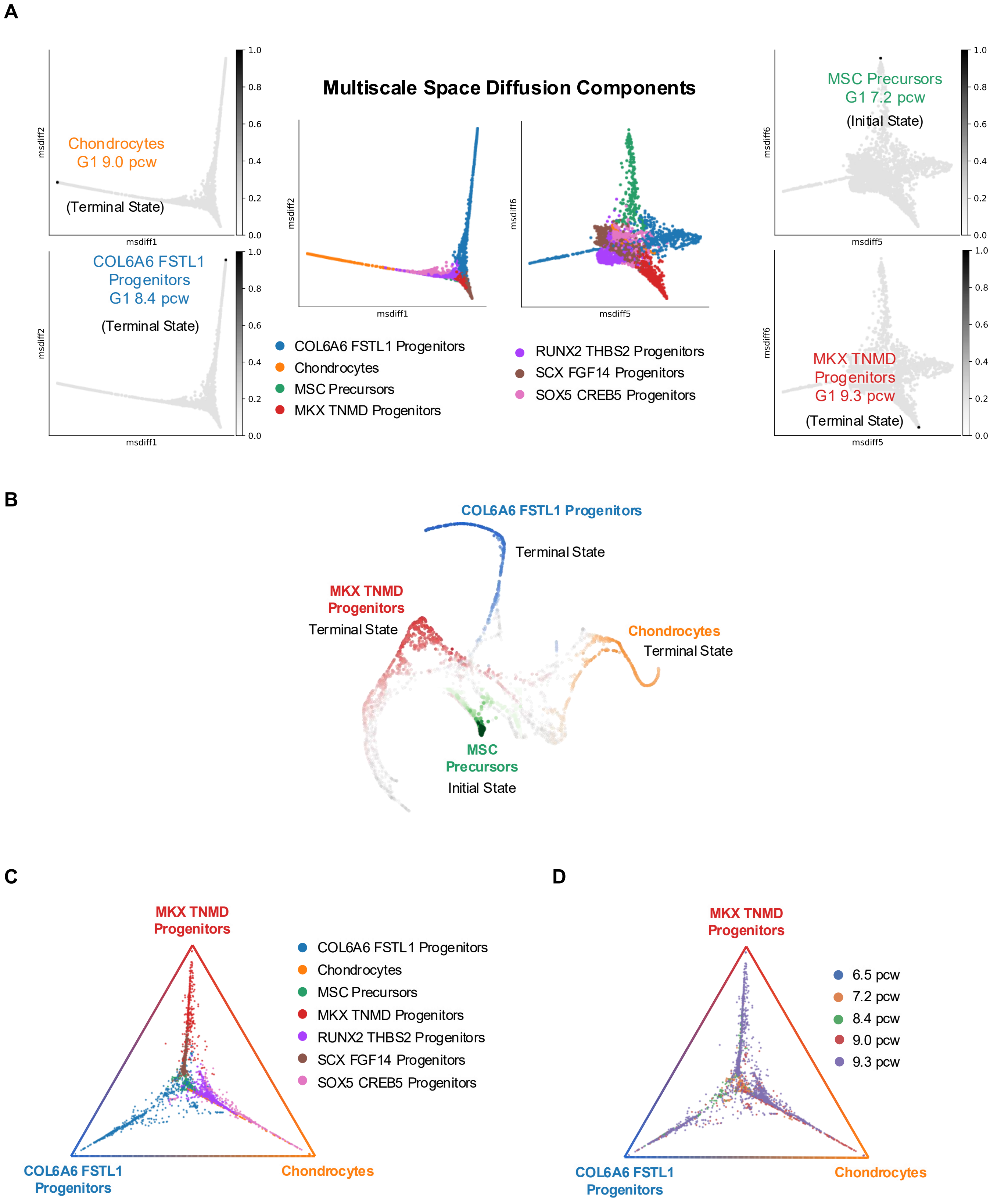
A
Multiscale Space Diffusion Components
Chondrocytes
G1 9.0 pcw
(Terminal State)
COL6A6 FSTL1
Progenitors
G1 8.4 pcw
(Terminal State)
MSC Precursors
G1 7.2 pcw
(Initial State)
MKX TNMD
Progenitors
G1 9.3 pcw
(Terminal State)
msdiff1
msdiff2
msdiff5
msdiff6
COL6A6 FSTL1 Progenitors
Chondrocytes
MSC Precursors
MKX TNMD Progenitors
RUNX2 THBS2 Progenitors
SCX FGF14 Progenitors
SOX5 CREB5 Progenitors
B
COL6A6 FSTL1 Progenitors
Terminal State
MKX TNMD
Progenitors
Terminal State
Chondrocytes
Terminal State
MSC
Precursors
Initial State
C
MKX TNMD
Progenitors
COL6A6 FSTL1
Progenitors
Chondrocytes
COL6A6 FSTL1 Progenitors
Chondrocytes
MSC Precursors
MKX TNMD Progenitors
RUNX2 THBS2 Progenitors
SCX FGF14 Progenitors
SOX5 CREB5 Progenitors
D
MKX TNMD
Progenitors
COL6A6 FSTL1
Progenitors
Chondrocytes
6.5 pcw
7.2 pcw
8.4 pcw
9.0 pcw
9.3 pcw

Figure S4. CellRank fate mapping of embryonic tendon cell differentiation.

(A) Selected initial and terminal states using multiscale space embedding components for Palantir pseudotime calculation. Multiscale space was calculated based on the Palantir diffusion map embeddings. (B) Force-directed graph showing the location of manually selected cells corresponding to the initial and terminal states. (C,D) Circular projections of fate probabilities coloured by (C) cell states or (D) donor ages in weeks post-conception (pcw) showing the trajectory and density of cells transitioning from initial MSC Precursors in the middle towards the specified terminal fates.

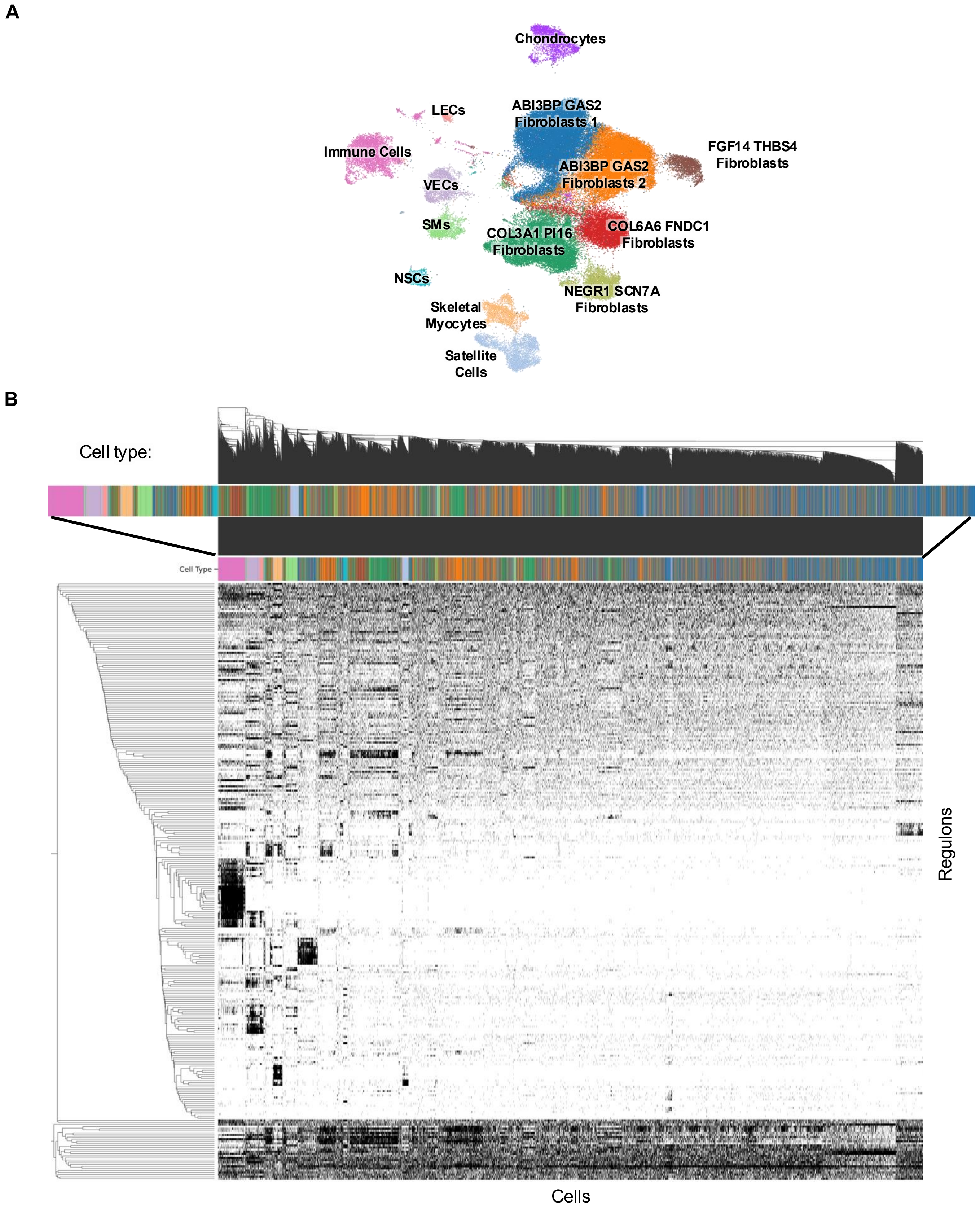
A
Chondrocytes
LECs
ABI3BP GAS2
Fibroblasts 1
Immune Cells
FGF14 THBS4
Fibroblasts
ABI3BP GAS2
Fibroblasts 2
VECs
SMs
COL3A1 PI16
Fibroblasts
COL6A6 FNDC1
Fibroblasts
NSCs
NEGR1 SCN7A
Fibroblasts
Skeletal
Myocytes
Satellite
Cells
B
Cell type:
Cell Type
Regulons
Cells

Figure S5. SCENIC analysis of cell type-specific regulon activity.

(A) UMAP showing annotated 12-20pcw foetal Achilles and quadriceps tendon cell types. (B) Clustermap showing regulon-based clustering in 20 post-conception week (pcw) foetal Achilles and quadriceps tendon cell types. Each row represents a regulon, and each column corresponds to an individual cell. Cell types are indicated by colours in the top bar, synchronised with the UMAP visualisation.

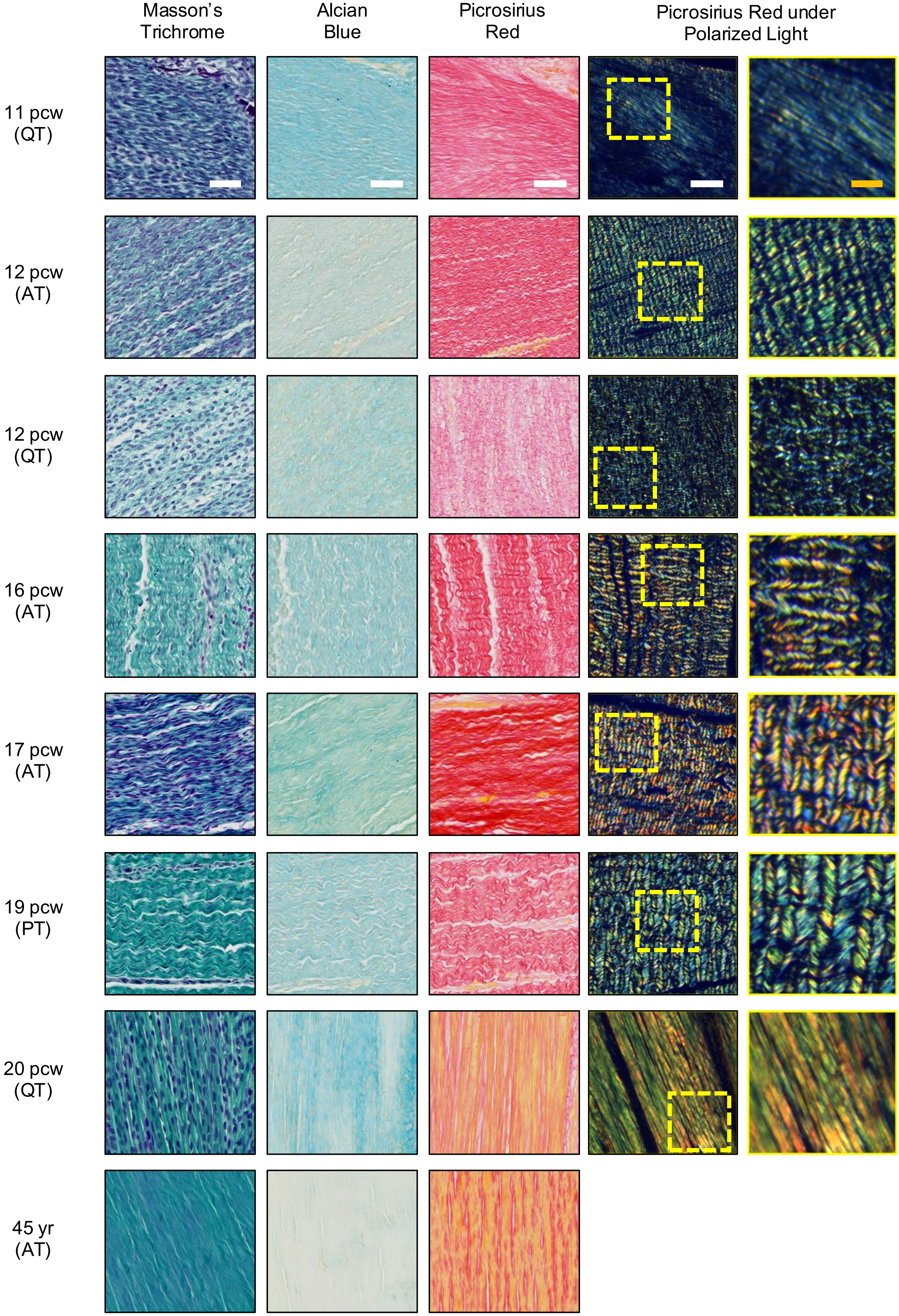

Masson's Trichrome
Alcian Blue
Picrosirius Red
Picrosirius Red under Polarized Light
11 pcw (QT)
12 pcw (AT)
12 pcw (QT)
16 pcw (AT)
17 pcw (AT)
19 pcw (PT)
20 pcw (QT)
45 yr (AT)

Figure S6. Photomicrographs of foetal and adult tendon tissues stained with masson's trichrome, alcian blue, and picrosirius red.

Brightfield microscopy was used for visualisation, with polarizing light filters additionally applied to the picrosirius red-stained tissues. Masson's trichrome stains for collagen fibres (blue), muscle fibres (red), and cell nuclei (dark purple). Alcian blue specifically stains for acidic mucopolysaccharides and glycosaminoglycans, which are components of proteoglycans and glycoproteins forming the ground substance (blue). Picrosirius red stains for collagen fibres, which appear red under brightfield microscopy and show varying colours (red, orange, yellow, and green) under polarized light, depending on the thickness and type of collagen. The size bars correspond to 50 µm (white) or 20 µm (orange). Examined tissues include Achilles tendon (AT), quadriceps tendon (QT), and patellar tendon (PT). Foetal tissues are denoted by post-conception weeks (pcw) and adult tissues by years (yr).

A

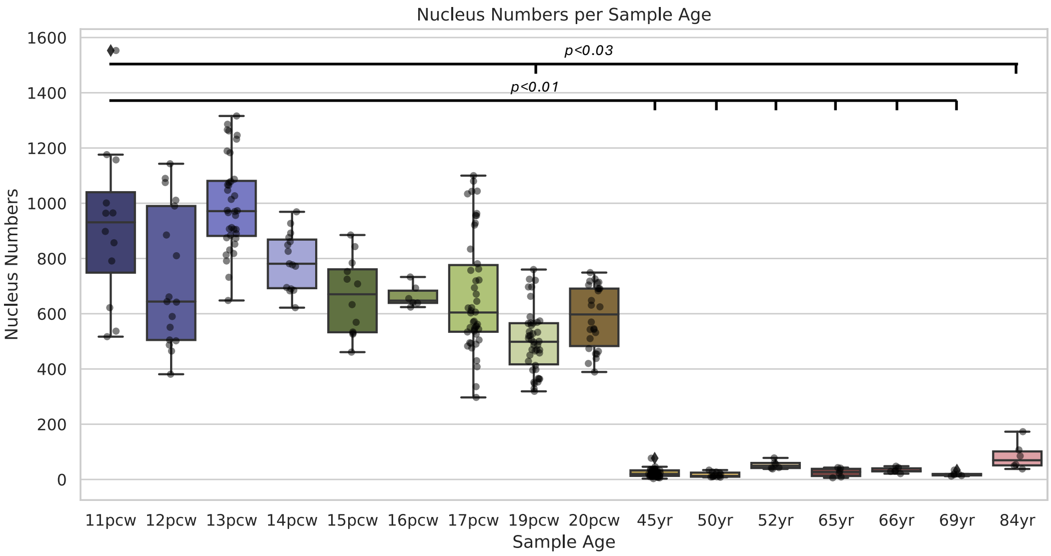

Nucleus Numbers per Sample Age
p<0.03
p<0.01
Nucleus Numbers
1600
1400
1200
1000
800
600
400
200
0
11pcw 12pcw 13pcw 14pcw 15pcw 16pcw 17pcw 19pcw 20pcw 45yr 50yr 52yr 65yr 66yr 69yr 84yr
Sample Age

B

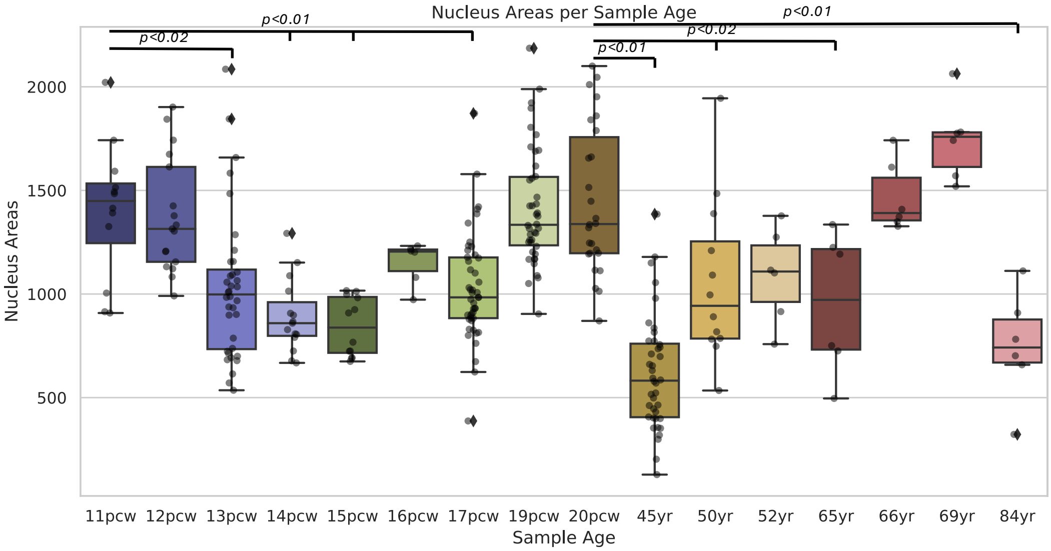

Nucleus Areas per Sample Age
p<0.01
p<0.02
p<0.01
p<0.02
p<0.01
Nucleus Areas
2000
1500
1000
500
11pcw 12pcw 13pcw 14pcw 15pcw 16pcw 17pcw 19pcw 20pcw 45yr 50yr 52yr 65yr 66yr 69yr 84yr
Sample Age

C

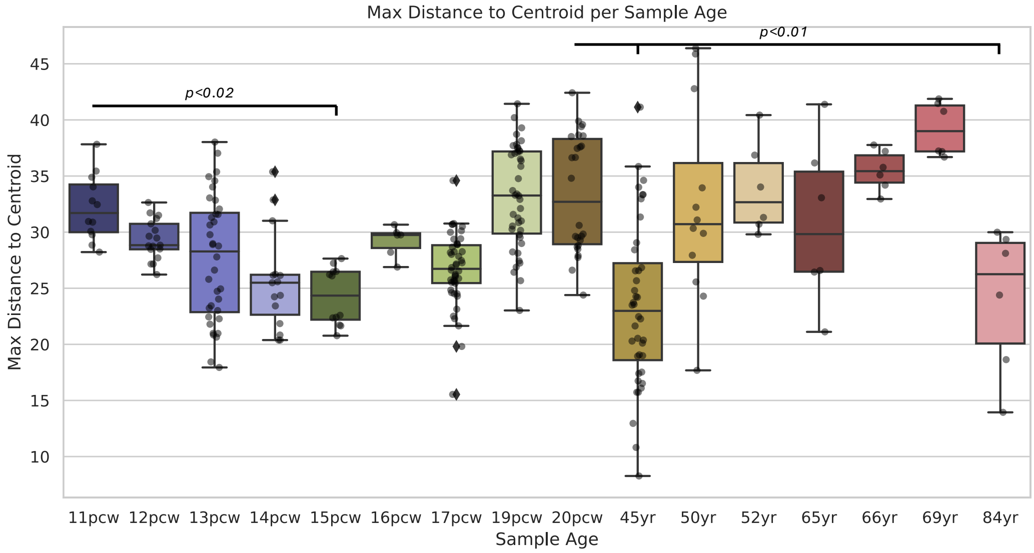

Max Distance to Centroid per Sample Age
p<0.01
p<0.02
Max Distance to Centroid
45
40
35
30
25
20
15
10
11pcw 12pcw 13pcw 14pcw 15pcw 16pcw 17pcw 19pcw 20pcw 45yr 50yr 52yr 65yr 66yr 69yr 84yr
Sample Age

Figure S7. Results of StarDist quantification of nuclear properties in haematoxylin-stained tissues.

Boxplots display (A) nuclei numbers, (B) nuclei areas, and (C) maximum distances to nuclei centroids for each sample age. Foetal tissues are denoted by post-conception weeks (pcw) and adult tissues by years (yr). Adult ages correspond to single donors. Statistical analysis was performed using a Kruskal-Wallis test followed by Dunn's post-hoc test for nuclei numbers (A), and ANOVA with Tukey HSD for nuclei areas and distances (B, C), measured in pixels within 250 μm^2 tiles. Significant comparisons against a single timepoint are shown: all foetal and adult comparisons against 11 pcw in (A), and foetal comparisons against 11 pcw and adult comparisons against 20 pcw in (B) and (C).

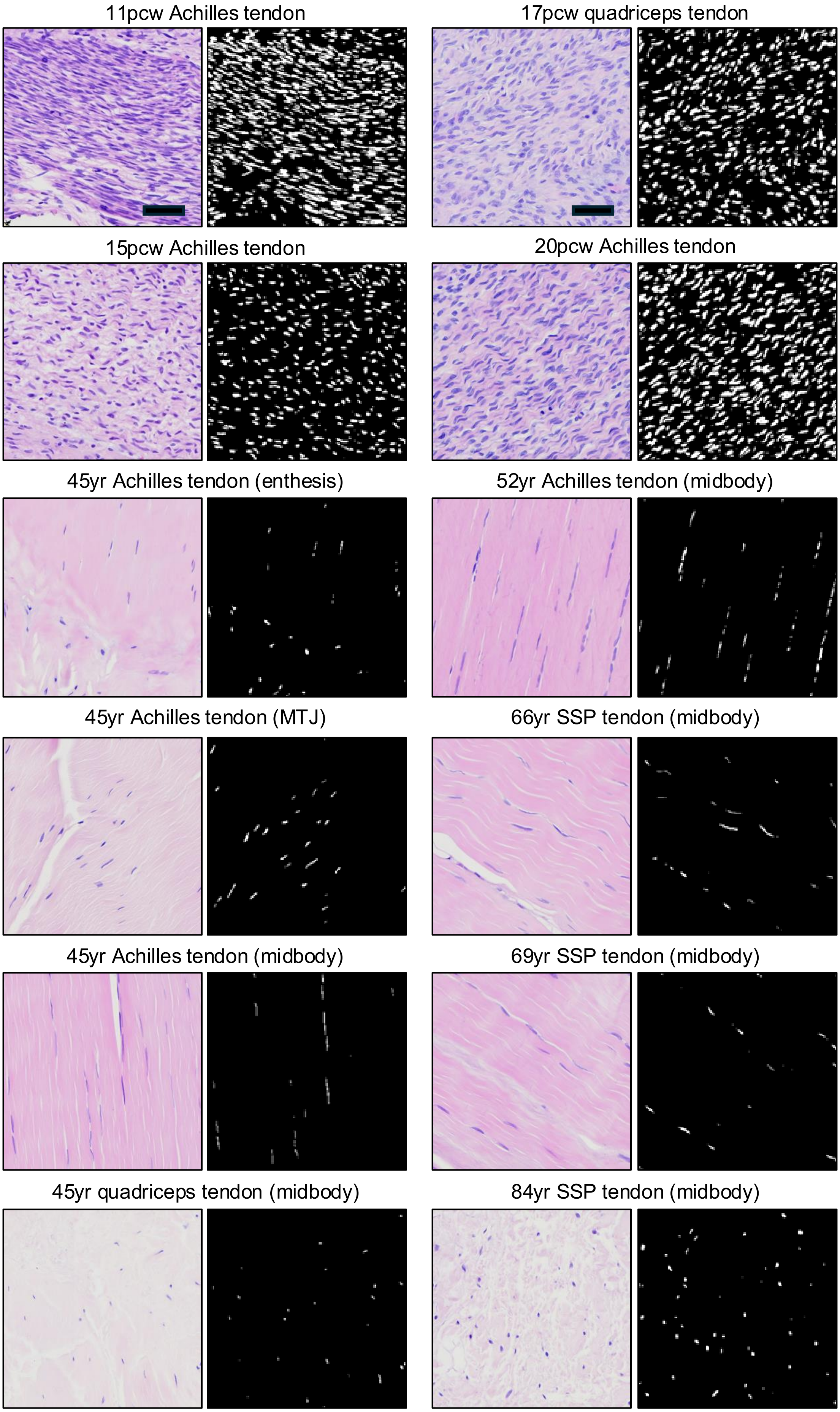
11pcw Achilles tendon
17pcw quadriceps tendon
15pcw Achilles tendon
20pcw Achilles tendon
45yr Achilles tendon (enthesis)
52yr Achilles tendon (midbody)
45yr Achilles tendon (MTJ)
66yr SSP tendon (midbody)
45yr Achilles tendon (midbody)
69yr SSP tendon (midbody)
45yr quadriceps tendon (midbody)
84yr SSP tendon (midbody)

Figure S8. Examples of foetal and adult tendon sample tiles used for StarDist nuclei segmentation.

Left panels show the original H&E-stained images, while right panels display the corresponding isolated haematoxylin channels used for nuclei segmentation and measurements with StarDist. Size bars correspond to 50 µm. Foetal tissues are denoted by post-conception weeks (pcw) and adult tissues by years (yr). SSP: supraspinatus tendon of the shoulder; MTJ: myotendinous junction.

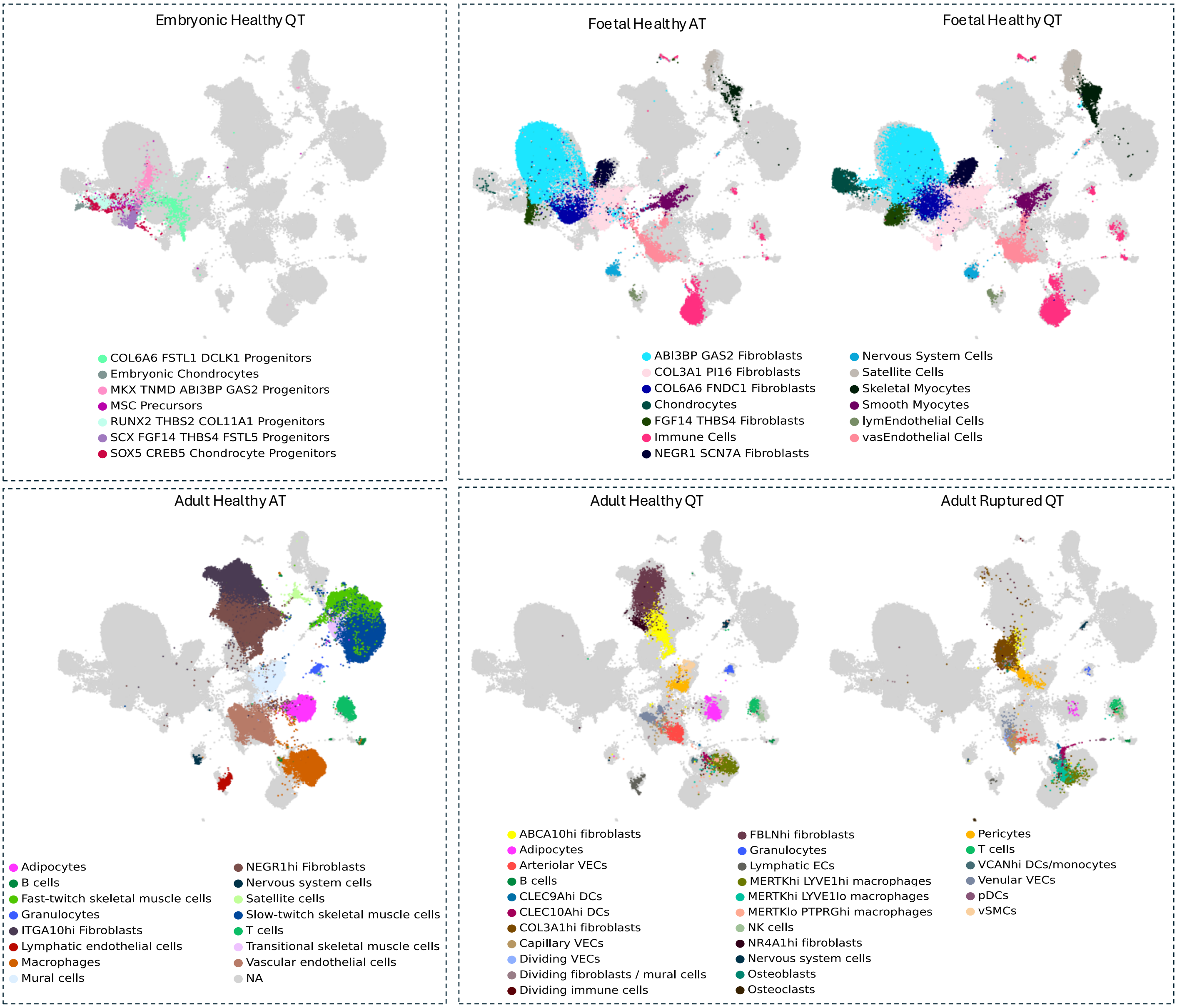

Embryonic Healthy QT
COL6A6 FSTL1 DCLK1 Progenitors
Embryonic Chondrocytes
MKX TNMD ABI3BP GAS2 Progenitors
MSC Precursors
RUNX2 THBS2 COL11A1 Progenitors
SCX FGF14 THBS4 FSTL5 Progenitors
SOX5 CREB5 Chondrocyte Progenitors
Foetal Healthy AT
Foetal Healthy QT
ABI3BP GAS2 Fibroblasts
COL3A1 PI16 Fibroblasts
COL6A6 FNDC1 Fibroblasts
Chondrocytes
FGF14 THBS4 Fibroblasts
Immune Cells
NEGR1 SCN7A Fibroblasts
Nervous System Cells
Satellite Cells
Skeletal Myocytes
Smooth Myocytes
lymEndothelial Cells
vasEndothelial Cells
Adult Healthy AT
Adipocytes
B cells
Fast-twitch skeletal muscle cells
Granulocytes
ITGA10hi Fibroblasts
Lymphatic endothelial cells
Macrophages
Mural cells
NEGR1hi Fibroblasts
Nervous system cells
Satellite cells
Slow-twitch skeletal muscle cells
T cells
Transitional skeletal muscle cells
Vascular endothelial cells
NA
Adult Healthy QT
Adult Ruptured QT
ABCA10hi fibroblasts
Adipocytes
Arteriolar VECs
B cells
CLEC9Ahi DCs
CLEC10Ahi DCs
COL3A1hi fibroblasts
Capillary VECs
Dividing VECs
Dividing fibroblasts / mural cells
Dividing immune cells
FBLNhi fibroblasts
Granulocytes
Lymphatic ECs
MERTKhi LYVE1hi macrophages
MERTKhi LYVE1lo macrophages
MERTKlo PTPRGhi macrophages
NK cells
NR4A1hi fibroblasts
Nervous system cells
Osteoblasts
Osteoclasts
Pericytes
T cells
VCANhi DCs/monocytes
Venular VECs
pDCs
vSMCs

Figure S9. Distribution of original cell type annotations from scANVI-integrated embryonic, foetal, and adult tendon datasets.

Annotated adult datasets were provided by Dr Carla Cohen (Achilles tendon) and Dr Jolet Mimpen (quadriceps tendon).

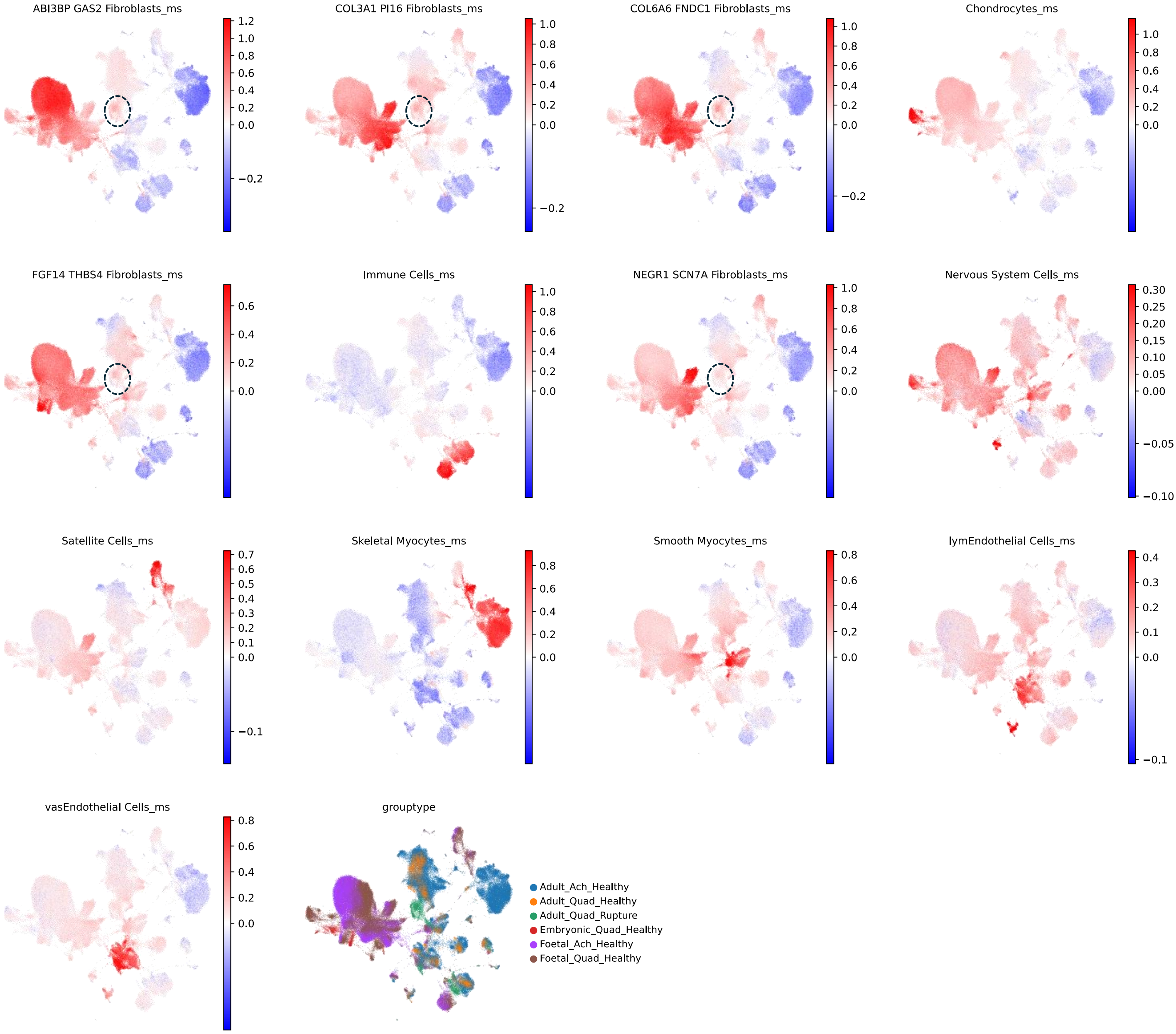
ABI3BP GAS2 Fibroblasts_ms
1.2
1.0
0.8
0.6
0.4
0.2
0.0
−0.2
COL3A1 PI16 Fibroblasts_ms
1.0
0.8
0.6
0.4
0.2
0.0
−0.2
COL6A6 FNDC1 Fibroblasts_ms
1.0
0.8
0.6
0.4
0.2
0.0
−0.2
Chondrocytes_ms
1.0
0.8
0.6
0.4
0.2
0.0
FGF14 THBS4 Fibroblasts_ms
0.6
0.4
0.2
0.0
Immune Cells_ms
1.0
0.8
0.6
0.4
0.2
0.0
NEGR1 SCN7A Fibroblasts_ms
1.0
0.8
0.6
0.4
0.2
0.0
Nervous System Cells_ms
0.30
0.25
0.20
0.15
0.10
0.05
0.00
−0.05
−0.10
Satellite Cells_ms
0.7
0.6
0.5
0.4
0.3
0.2
0.1
0.0
−0.1
Skeletal Myocytes_ms
0.8
0.6
0.4
0.2
0.0
Smooth Myocytes_ms
0.8
0.6
0.4
0.2
0.0
lymEndothelial Cells_ms
0.4
0.3
0.2
0.1
0.0
−0.1
vasEndothelial Cells_ms
0.8
0.6
0.4
0.2
0.0
grouptype
Adult_Ach_Healthy
Adult_Quad_Healthy
Adult_Quad_Rupture
Embryonic_Quad_Healthy
Foetal_Ach_Healthy
Foetal_Quad_Healthy

Figure S10. Marker gene overlap between foetal and adult tendon cell types.

UMAPs show the spatial distribution of gene expression scores for the same top 50 DEGs specific to individual foetal tendon cell types (see Figure 6A). Colour intensity is scaled to the 99th percentile of the score distribution and indicates the relative expression level of the markers within the cells, with red corresponding to high expression of the top 50 foetal DEGs. Black ovals highlight the rupture-specific fibroblast population.

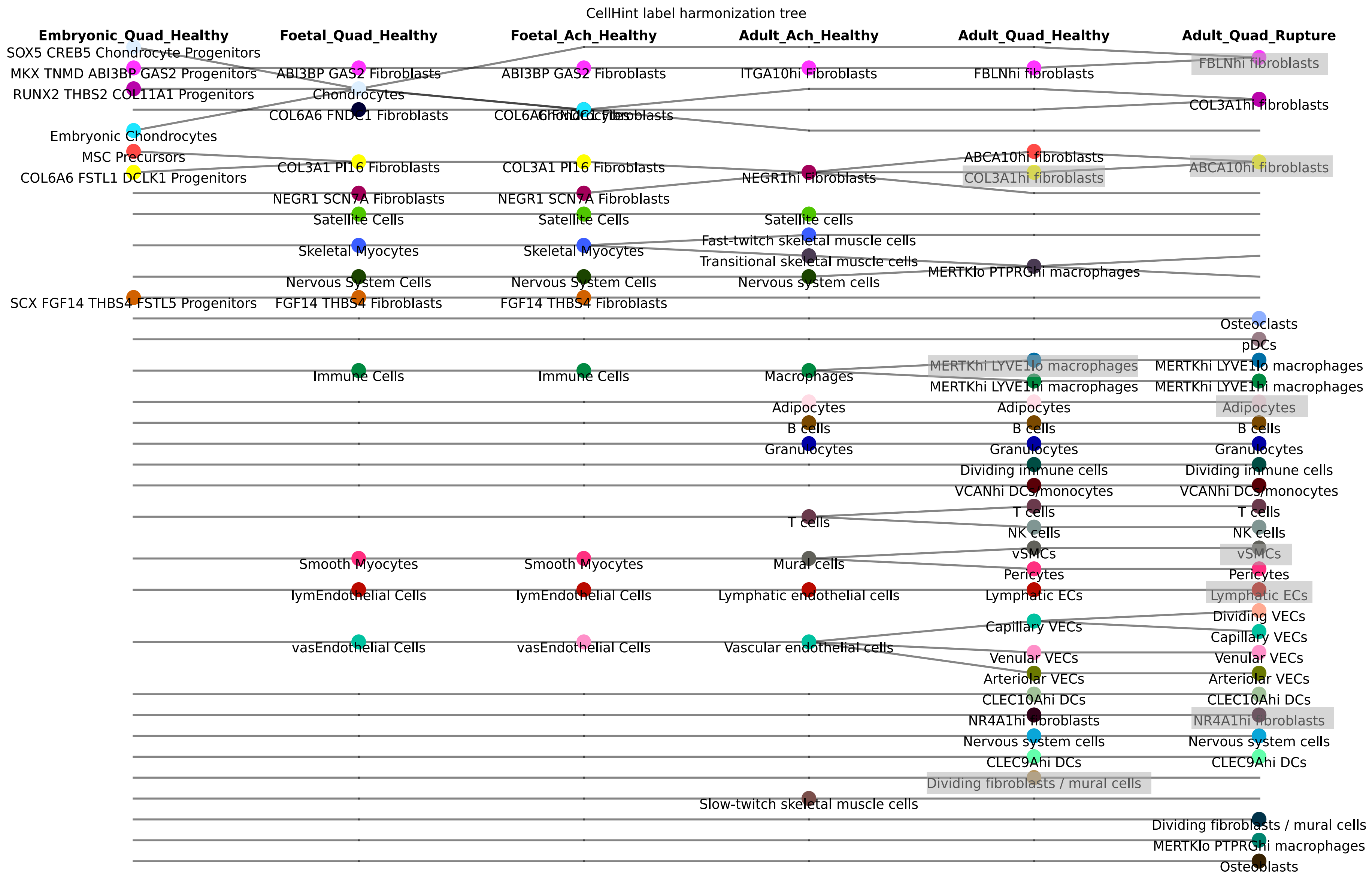
CellHint label harmonization tree
Embryonic_Quad_Healthy
Foetal_Quad_Healthy
Foetal_Ach_Healthy
Adult_Ach_Healthy
Adult_Quad_Healthy
Adult_Quad_Rupture
SOX5 CREB5 Chondrocyte Progenitors
MKX TNMD ABI3BP GAS2 Progenitors
RUNX2 THBS2 COL11A1 Progenitors
Embryonic Chondrocytes
MSC Precursors
COL6A6 FSTL1 DCLK1 Progenitors
SCX FGF14 THBS4 FSTL5 Progenitors
ABI3BP GAS2 Fibroblasts
Chondrocytes
COL6A6 FNDC1 Fibroblasts
COL3A1 PI16 Fibroblasts
NEGR1 SCN7A Fibroblasts
Satellite Cells
Skeletal Myocytes
Nervous System Cells
FGF14 THBS4 Fibroblasts
Immune Cells
Smooth Myocytes
lymEndothelial Cells
vasEndothelial Cells
ITGA10hi Fibroblasts
NEGR1hi Fibroblasts
Satellite cells
Fast-twitch skeletal muscle cells
Transitional skeletal muscle cells
Nervous system cells
Macrophages
Adipocytes
B cells
Granulocytes
T cells
Mural cells
Lymphatic endothelial cells
Vascular endothelial cells
Slow-twitch skeletal muscle cells
FBLNhi fibroblasts
ABCA10hi fibroblasts
COL3A1hi fibroblasts
MERTKlo PTPRChi macrophages
MERTKhi LYVE1lo macrophages
MERTKhi LYVE1hi macrophages
Dividing immune cells
VCANhi DCs/monocytes
NK cells
vSMCs
Pericytes
Lymphatic ECs
Capillary VECs
Venular VECs
Arteriolar VECs
CLEC10Ahi DCs
NR4A1hi fibroblasts
CLEC9Ahi DCs
Dividing fibroblasts / mural cells
Osteoclasts
pDCs
Dividing VECs
Osteoblasts

Figure S11. CellHint harmonisation of annotated cell types across datasets and conditions.

Tree plot shows transcriptional relationships between matched cell types, based on distances calculated from batch-corrected scVI embeddings. Greyed out cell types represent unusually low cell populations (less than 102 cells) within a given dataset.

Adult Ruptured COL3A1hi Fibroblasts vs Foetal COL6A6 FNDC1 Fibroblasts

A

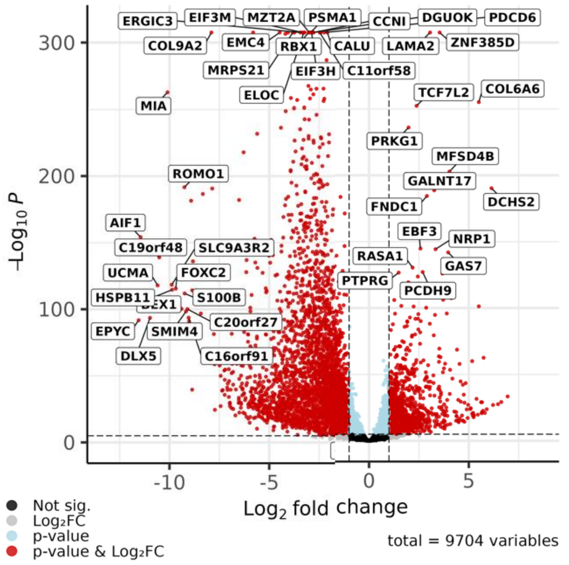

Downregulated BH FDR <0.01 (571):	Upregulated BH FDR <0.01 (325):
- Energy Metabolism and Mitochondrial Function<br>- Nucleotide and Ribose Phosphate Mechanism<br>- Protein Metabolism and Folding<br>- RNA Metabolism<br>- Protein Translation<br>- Cell Cycle and Division<br>- Response to Stress and Apoptosis<br>- Autophagy and Senescence<br>- Responses to TGF-b Signalling	- Cell Adhesion and Extracellular Matrix Organisation<br>- Muscle Development and Contraction<br>- Calcium Ion Transport and Homeostasis<br>- Neuron and Synapse Development and Organisation<br>- Cell Signalling and Transduction (Wnt signalling, GTPase, Rac, Rho, and GPCR transduction pathways)<br>- Organ/Tissue Developmental Processes and Cell Growth<br>- Vascular Development and Blood Circulation<br>- Cell Migration and Morphogenesis<br>- Immune and Inflammatory Responses

B

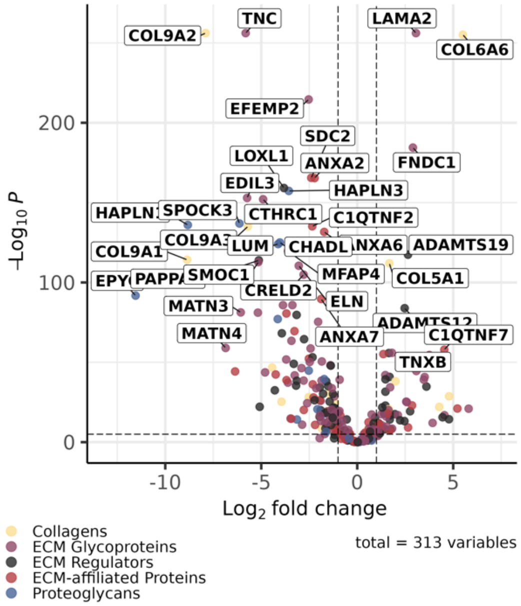

Downregulated padj <0.01, log2FC < -1 (127):	Upregulated padj <0.01, log2FC > 1 (66):
**Collagens:** COL9A1, COL9A2, COL9A3, COL8A1, COL2A1, COL26A1, COL11A2, COL11A1, COL15A1, COL8A2, COL13A1<br>**Proteoglycans:** EPYC, HAPLN1, SPOCK3, PODNL1, LUM, CHADL, PRELP, FMOD, ACAN, OGN, SPOCK1, OMD<br>**Glycoproteins:** MATN4, MATN3, TNC, EDIL3, EFEMP1, SMOC1, CTHRC1, MATN1, MFAP2, PCOLCE2, CILP2, MFAP4, NPNT, NELL2, LTBP1, IGFBP2, ELN, TSKU, FBN3, CRELD2, VWA1, CRISPLD2, EFEMP2, MFGE8, IGFBP5, MFAP1, IGFBP3, GAS6, RSPO3, FBLN2, IGFBP4, COMP, MGP, TGFBI, CRISPLD1, FBLN1, SMOC2, THBS2, EMID1, AEBP1, EMILIN1, SRPX, SPARC<br>**ECM-affiliated Proteins:** LGALS1, SDC4, GPC1, CLEC11A, SEMA3E, SDC1, SDC2, C1QTNF3, GPC5, SEMA5B, C1QTNF2, C1QTNF4, ANXA2, PLXNA4, ANXA5, ANXA7, ANXA6, LGALS3, GREM1, C1QTNF1, C1QTNF6<br>**ECM Regulators:** PAPPA2, CSTB, CST3, TIMP1, LOXL1, HYAL2, SERPINF1, MMP11, CTSD, CTSH, CTSK, SERPINH1, MMP28, EGLN2, CTSA, CTSB, PLOD3, PLOD1, SERPING1, P4HTM, TIMP3, SERPINI1, ADAMTS6, ADAMTS17, MMP2, SERPINE1, CTSC, MMP14, KAZALD1, ADAMTSL1, ADAM15	**Collagens:** COL6A6, COL5A3, COL10A1, COL6A3, COL5A1, COL18A1<br>**Glycoproteins:** HMCN2, EYS, FGL2, TNXB, NTN1, MFAP5, EMILIN2, LAMA2, FNDC1, ECM2, IGSF10, POSTN, NTNG1, DPT, LAMB1, FBN1, LAMA1, TNFAIP6, MATN2, HMCN1, RSPO2, SLIT2, ABI3BP, SPON2, POMZP3<br>**ECM-affiliated Proteins:** FREM3, C1QTNF7, PLXNC1, EMCN, FREM1, SEMA6C, SEMA3C, ELFN1, SEMA6D, SEMA3D, PLXNA3<br>**ECM Regulators:** MMP19, MMP21, ITIH5, ADAM33, ADAMTS19, ADAMTS12, PCSK6, PAPPA, ADAM32, ADAM19, ADAM12, ADAMTS2, ADAMTSL3, ADAM22, SULF2, ADAMTS9, CD109, ADAMTS15, ADAMTS5, SULF1, FAM20A, FAM20C, MMP24, SERPINB1

Figure S12. Analysis of significantly up- and downregulated genes and pathways in adult ruptured COL3A1hi Fibroblasts vs foetal COL6A6 FNDC1 fibroblasts.

(A) Volcano plot illustrating significantly up- and downregulated genes determined by the DESeq2 Wald test (p-adjusted < 0.01, log2FC ±1). The table to the right summarises the results of gene ontology (GO) biological processes (BP) analyses conducted using the gProfiler g:GOSt tool (Raudvere et al., 2019). GO:BP pathways sized 20-500 terms with Benjamini-Hochberg (BH) FDR values <0.01 were analysed and categorised by common functions (e.g. different pathways related to apoptosis were grouped together into an "Apoptosis" category). The numbers in brackets show the total number of different GO:BP pathways meeting the BH FDR criteria. (B) Volcano plot and accompanying table showing significantly up- and downregulated matrisomal genes, identified using the DESeq2 Wald test (p-adjusted < 0.01, log2FC ±1).

Adult Ruptured COL3A1hi Fibroblasts vs Foetal Chondrocytes

A

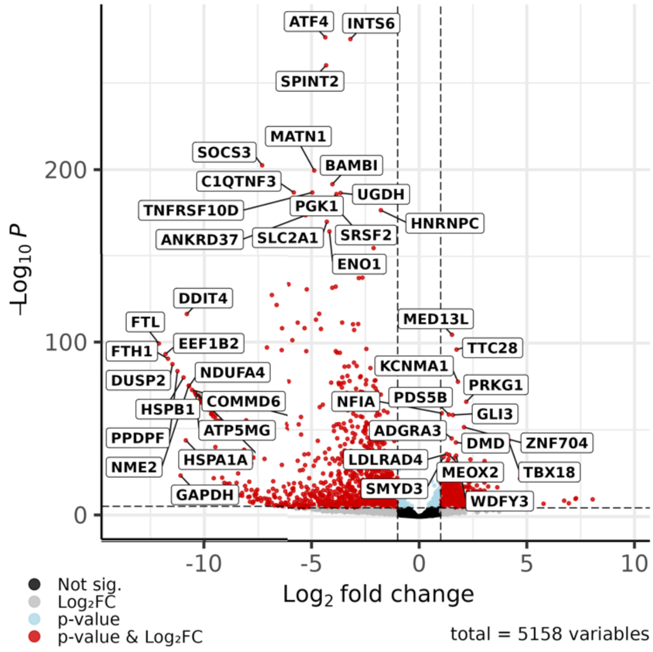

Downregulated BH FDR <0.01 (519):	Upregulated BH FDR <0.01 (126):
- Energy Metabolism and Mitochondrial Function<br>- Nucleotide and Ribose Phosphate Mechanism<br>- Protein Metabolism and Folding<br>- RNA Metabolism<br>- Protein Translation<br>- Cell Cycle and Division<br>- Response to Stress and Apoptosis<br>- Autophagy	- Cell Migration and Adhesion<br>- Cell Morphogenesis<br>- Muscle Cell Development and Differentiation<br>- Neuron and Axon Development<br>- Limb Development and Morphogenesis<br>- Cell Signalling (GTPase, Wnt, TGF-b, Smoothened, TOR and insulin)<br>- Cell Cycle and Division<br>- Metabolism and Energy Production<br>- Protein Modification and Degradation

B

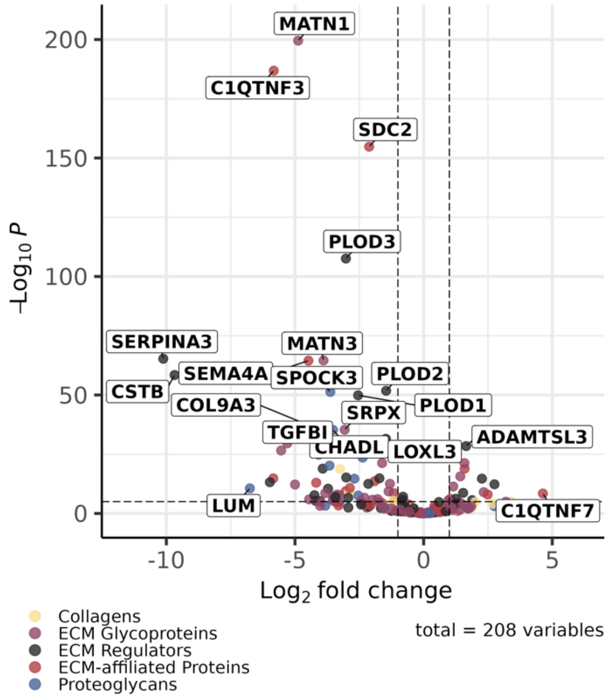

Downregulated padj <0.01, log2FC < -1 (78):	Upregulated padj <0.01, log2FC > 1 (28):
**Collagens:** COL9A3, COL9A2, COL6A2, COL9A1, COL2A1, COL15A1, COL8A1<br>**Proteoglycans:** LUM, EPYC, PRELP, SPOCK3, CHADL, DCN, HAPLN1, CHAD, SPOCK1, FMOD<br>**Glycoproteins:** MATN4, TGFBI, MGP, MATN1, IGFBP4, CTHRC1, EFEMP1, MFAP4, MATN3, VWA1, IGFBP7, PCOLCE, IGFBP2, IGFBP5, EFEMP2, SRPX, FBLN1, MFAP2, COMP, PCOLCE2, GAS6, LAMB2, NID2, AEBP1<br>**ECM-affiliated Proteins:** GPC3, C1QTNF3, SEMA4A, LGALS3, LGALS1, ANXA5, ANXA2, GPC1, PLXNA2, SDC2, SDC1, GPC5, LMAN1, ANXA6, ANXA7<br>**ECM Regulators:** SERPINA3, CSTB, TIMP1, SERPINH1, CTSL, CTSD, LOXL1, EGLN3, SERPINE1, PLOD3, P4HA2, SERPINE2, CST3, PLOD1, TIMP3, LOXL2, MMP2, P4HA1, CTSB, LOXL3, PLOD2	**Collagens:** COL1A1, COL5A2, COL24A1, COL12A1, COL25A1<br>**Proteoglycans:** ASPN<br>**Glycoproteins:** ECM2, FNDC1, LAMA2, SPON1, LTBP2, THSD4, SLIT3, SMOC1, CRISPLD1, SVEP1, TNC<br>**ECM-affiliated Proteins:** C1QTNF7, SEMA6D, PLXDC2, COLEC12<br>**ECM Regulators:** CD109, ADAM12, ADAM22, ADAMTSL3, ADAMTS19, FAM20C, MMP16

Figure S13. Analysis of significantly up- and downregulated genes and pathways in adult ruptured COL3A1hi Fibroblasts vs foetal Chondrocytes.

(A) Volcano plot illustrating significantly up- and downregulated genes determined by the DESeq2 Wald test (p-adjusted < 0.01, log2FC ±1). The table to the right summarises the results of gene ontology (GO) biological processes (BP) analyses conducted using the gProfiler g:GOSt tool (Raudvere et al., 2019). GO:BP pathways sized 20-500 terms with Benjamini-Hochberg (BH) FDR values <0.01 were analysed and categorised by common functions (e.g. different pathways related to apoptosis were grouped together into an “Apoptosis” category). The numbers in brackets show the total number of different GO:BP pathways meeting the BH FDR criteria. (B) Volcano plot and accompanying table showing significantly up- and downregulated matrisomal genes, identified using the DESeq2 Wald test (p-adjusted < 0.01, log2FC ±1).

Adult Healthy FBLNhi Fibroblasts vs Foetal ABI3BP GAS2 Fibroblasts

A

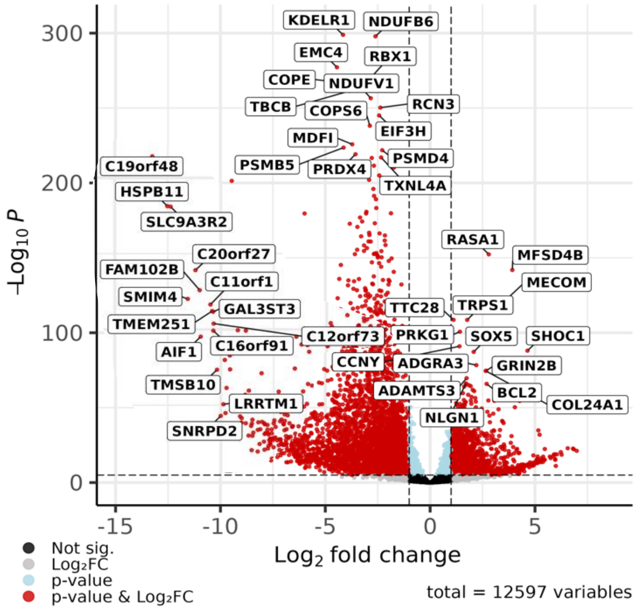

<table>
<tr><th><u>Downregulated BH FDR <0.01 (573):</u></th><th><u>Upregulated BH FDR <0.01 (141):</u></th></tr>
<tr><td>- Energy Metabolism and Mitochondrial Function
- Nucleotide and Ribose Phosphate Mechanism
- Protein Metabolism and Folding
- RNA Metabolism
- Protein Translation
- Proteolysis, Protein Catabolism
- Cell Cycle and Division
- Response to Stress and Apoptosis</td><td>- Cell Migration and Adhesion
- ECM Organisation
- Cell Morphogenesis
- Neuron and Axon Development
- Limb Development and Morphogenesis
- Cell Signalling (GTPase, Wnt, TGF-b, Smoothened, P3PKB)
- Ion Transport
- Protein and Lipid Metabolism</td></tr>
</table>

B

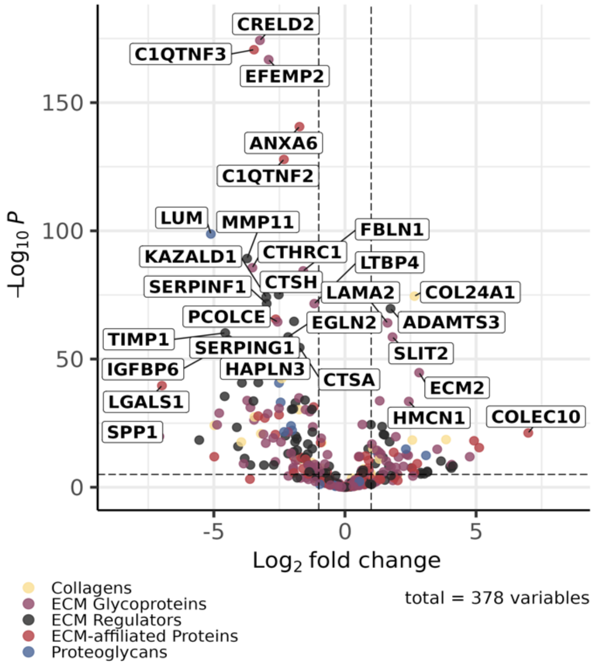

<table>
<tr><th><u>Downregulated padj <0.01, log2FC < -1 (124):</u></th><th><u>Upregulated padj <0.01, log2FC > 1 (74):</u></th></tr>
<tr><td>Collagens: COL9A1, COL22A1, COL9A3, COL4A1, COL3A1, COL26A1, COL6A2, COL4A2, COL2A1, COL25A1
Proteoglycans: LUM, HAPLN3, ASPN, DCN, SPOCK1, BGN, OGN, KERA, CHADL
Glycoproteins: SPP1, IGFBP2, IGFBP4, IGFBP6, EFEMP1, MFAP4, MFAP2, CTHRC1, BGLAP, CRELD2, MGP, TSKU, VWA1, EFEMP2, RSPO3, FBN3, PCOLCE, TNFAIP6, IGFBP5, SPARCL1, LAMC3, VWCE, NTNG2, IGFBP3, NTNG1, FBLN2, MFAP1, SPON2, NTN4, FBLN1, EMILIN1, IGFBP7, THBS4, LGI4, MFGE8, NELL2, FBLN5, LTBP4, SPARC, TNC, TGFBI
ECM-affiliated Proteins: LGALS1, CLEC14A, ANXA8, C1QTNF3, GPC5, GPC3, ANXA2, ANXA1, C1QTNF2, SDC4, GPC1, CLEC11A, SEMA4A, SEMA3A, SEMA7A, ANXA5, ANXA6, ANXA7, C1QL1
ECM Regulators: CSTB, CST3, TIMP1, SERPING1, AGT, C17orf58, HYAL2, A2M, MMP11, CTSC, TLL1, SERPINE2, F13A1, KAZALD1, SERPINF1, PLAT, CTSH, CTSK, CTSL, CTSZ, LOXL1, ADAM15, CTSF, HTRA3, MMP2, SERPINE1, CTSA, CTSD, P4HTM, P4HA2, MMP14, MMP23B, EGLN2, EGLN3, TIMP3, SERPINB6, PLOD1, PLOD3, MMP15, OGFOD2</td><td>Collagens: COL10A1, COL24A1, COL6A6, COL4A3, COL6A3, COL5A3, COL8A1, COL27A1
Proteoglycans: IMPG2, OMD, ACAN
Glycoproteins: EYS, MMRN2, SNED1, ECM2, TSPEAR, HMCN1, NDNF, GLDN, SMOC1, NTN1, LGI2, RSPO2, MATN2, FGL2, SLIT2, COMP, LAMA2, SPON1, POMZP3, LAMA1, CRIM1, CILP2, ABI3BP, LAMA3, CRISPLD2, SVEP1
ECM-affiliated Proteins: COLEC10, FREM3, C1QTNF7, PLXNC1, MUC20, SEMA4G, EMCN, MUC4, MUC1, SEMA3D, PLXNB1, SFTPD, CLEC4A
ECM Regulators: SERPIND1, MASP2, MMP19, MMP21, CELA2B, ADAM21, ADAMTS18, A2ML1, MEP1B, ADAMTS13, HPSE2, PAPPA, ADAMTS3, CD109, ADAM32, HYAL1, ADAMTS19, ADAMTS17, ADAMTS14, ADAM22, ADAMTS6, LOXL3, ADAM12, ADAMTSL2</td></tr>
</table>

Figure S14. Analysis of significantly up- and downregulated genes and pathways in adult healthy FBLNhi Fibroblasts vs foetal ABI3BP GAS2 fibroblasts.

(A) Volcano plot illustrating significantly up- and downregulated genes determined by the DESeq2 Wald test (p-adjusted < 0.01, log2FC ±1). The table to the right summarises the results of gene ontology (GO) biological processes (BP) analyses conducted using the gProfiler g:GOSt tool (Raudvere et al., 2019). GO:BP pathways sized 20-500 terms with Benjamini-Hochberg (BH) FDR values <0.01 were analysed and categorised by common functions (e.g. different pathways related to apoptosis were grouped together into an “Apoptosis” category). The numbers in brackets show the total number of different GO:BP pathways meeting the BH FDR criteria. (B) Volcano plot and accompanying table showing significantly up- and downregulated matrisomal genes, identified using the DESeq2 Wald test (p-adjusted < 0.01, log2FC ±1).

Adult Healthy ABCA10hi Fibroblasts vs Foetal COL3A1 PI16 Fibroblasts

A

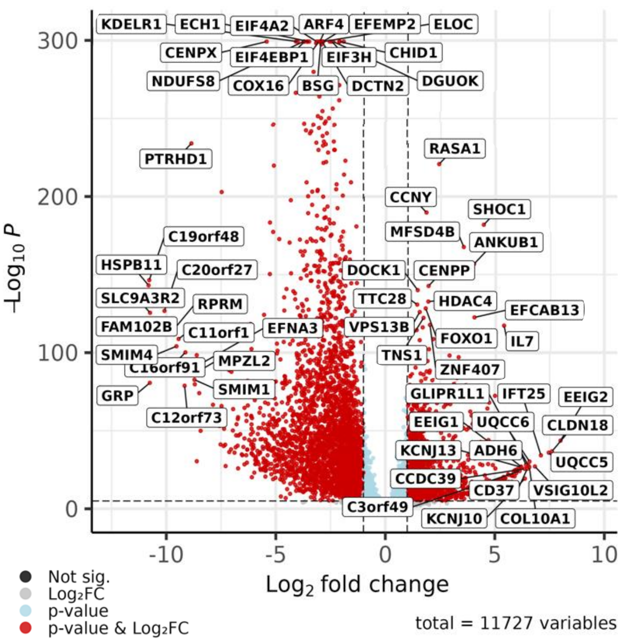

Downregulated BH FDR <0.01 (482):	Upregulated BH FDR <0.01 (186):
- Energy Metabolism and Mitochondrial Function<br>- Nucleotide and Ribose Phosphate Mechanism<br>- Protein Metabolism and Folding<br>- RNA Metabolism<br>- Protein Translation<br>- Proteolysis, Protein Catabolism<br>- Cell Cycle and Division<br>- Response to Stress and Apoptosis<br>- "Epithelial-to-mesenchymal transition"<br>- "Stem cell population maintenance"	- Cell Migration and Adhesion<br>- ECM Organisation<br>- Cell Morphogenesis<br>- Neuron and Axon Development<br>- Limb Development and Morphogenesis<br>- Cell Signalling (GTPase, Wnt, TGF-b, Smoothened, P3PKB)<br>- Ion Transport<br>- Protein and Lipid Metabolism

B

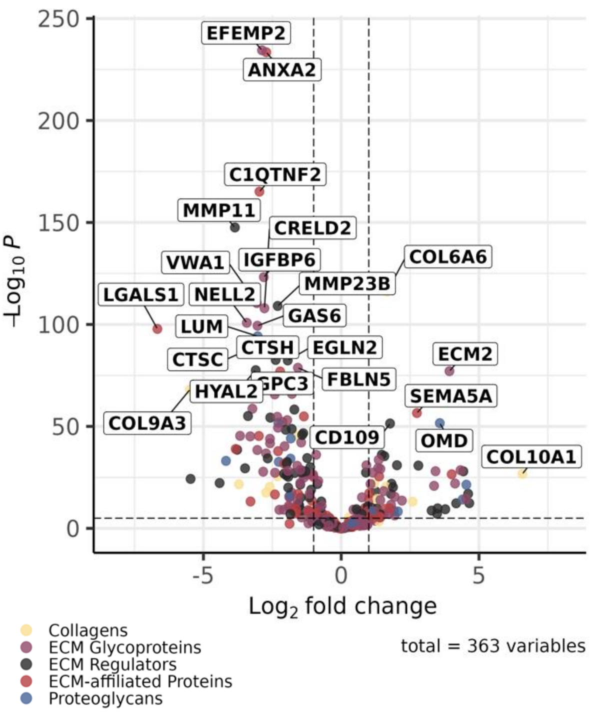

Downregulated padj <0.01, log2FC < -1 (124):	Upregulated padj <0.01, log2FC > 1 (74):
**Collagens:** COL9A3, COL9A1, COL9A2, COL26A1, COL13A1, COL6A2, COL2A1, COL1A1<br>**Proteoglycans:** SPOCK3, LUM, ASPN, FMOD, HAPLN3, DCN, OGN<br>**Glycoproteins:** BGLAP, NELL2, MFAP2, MFAP4, VWA1, CTHRC1, GAS6, EFEMP2, MGP, IGFBP6, CRELD2, RELN, VWCE, PCOLCE, TGFBI, IGFBP3, TSKU, FBN3, MATN4, FBLN1, SPARC, SRPX, IGFBP2, FRAS1, EFEMP1, AEBP1, COCH, FBLN5, VWA5A, TNFAIP6, EMILIN1, MFAP1, EDIL3, IGFBP5, FBLN2, NDNF, MFGE8, PXDNL, LGI4, SPON2, NID2<br>**ECM-affiliated Proteins:** LGALS1, ANXA3, ANXA2, ANXA5, GPC3, ANXA1, CLEC2B, CLEC14A, SDC1, SDC4, SEMA3A, SEMA4A, PARM1, C1QTNF2, C1QTNF3, C1QTNF4, C1QTNF6, ANXA6, ANXA8, LMAN1, GPC1, SDC3, SEMA4F, SEMA3D, SEMA6D, PLXNA3<br>**ECM Regulators:** MASP1, CSTB, MMP11, AGT, CST3, CTSC, TIMP1, CTSD, CTSH, SERPINF1, CTSD, CTSF, LOXL1, SERPINE1, P4HTM, HPSE2, EGLN2, PAPPA2, MMP23B, MMP17, MMP2, ADAMTS1, ADAMTSL2, ADAM15, ADAMTSL3, PLOD1, PLOD3, P4HTM, SERPINB6, SERPING1, LOX, F10, C17orf58	**Collagens:** COL10A1, COL4A3, COL6A6, COL5A3, COL28A1, COL4A1, COL6A3<br>**Proteoglycans:** PRG4, CHAD, OMD, IMPG2, PODN<br>**Glycoproteins:** MMRN2, EYS, NTN4, ECM2, SNED1, VIT, TNN, POMZP3, HMCN2, GLDN, PCOLCE2, EGFLAM, SPON1, LAMA1, MATN2, HMCN1, FBN1, RSPO2, LAMA4, IGFBP7, CRIM1, NTNG1<br>**ECM-affiliated Proteins:** FREM3, SEMA5A, EMCN, PLXDC1, PLXNA2, GPC6, MUC20, CLEC3B, PLXND1, CLEC2L, SEMA5B, SEMA3C, CLEC2D, C1QTNF7<br>**ECM Regulators:** MASP2, MMP21, ADAMTS16, ADAM21, ADAMTS18, A2M, SERPINE3, MEP1B, ADAMTS14, ADAM32, ADAM19, CD109, TLL1, ADAMTS3, ADAMTSL3, ADAMTS19, MMP19, ADAMTS15, ADAM12, MMP24, EGLN3, MMP16, LOXL3

Figure S15. Analysis of significantly up- and downregulated genes and pathways in adult healthy ABCA10 fibroblasts vs foetal COL3A1 PI16 fibroblasts.

(A) Volcano plot illustrating significantly up- and downregulated genes determined by the DESeq2 Wald test (p-adjusted < 0.01, log2FC ±1). The table to the right summarises the results of gene ontology (GO) biological processes (BP) analyses conducted using the gProfiler g:GOSt tool (Raudvere et al., 2019). GO:BP pathways sized 20-500 terms with Benjamini-Hochberg (BH) FDR values <0.01 were analysed and categorised by common functions (e.g. different pathways related to apoptosis were grouped together into an “Apoptosis” category). The numbers in brackets show the total number of different GO:BP pathways meeting the BH FDR criteria. (B) Volcano plot and accompanying table showing significantly up- and downregulated matrisomal genes, identified using the DESeq2 Wald test (p-adjusted < 0.01, log2FC ±1).

A

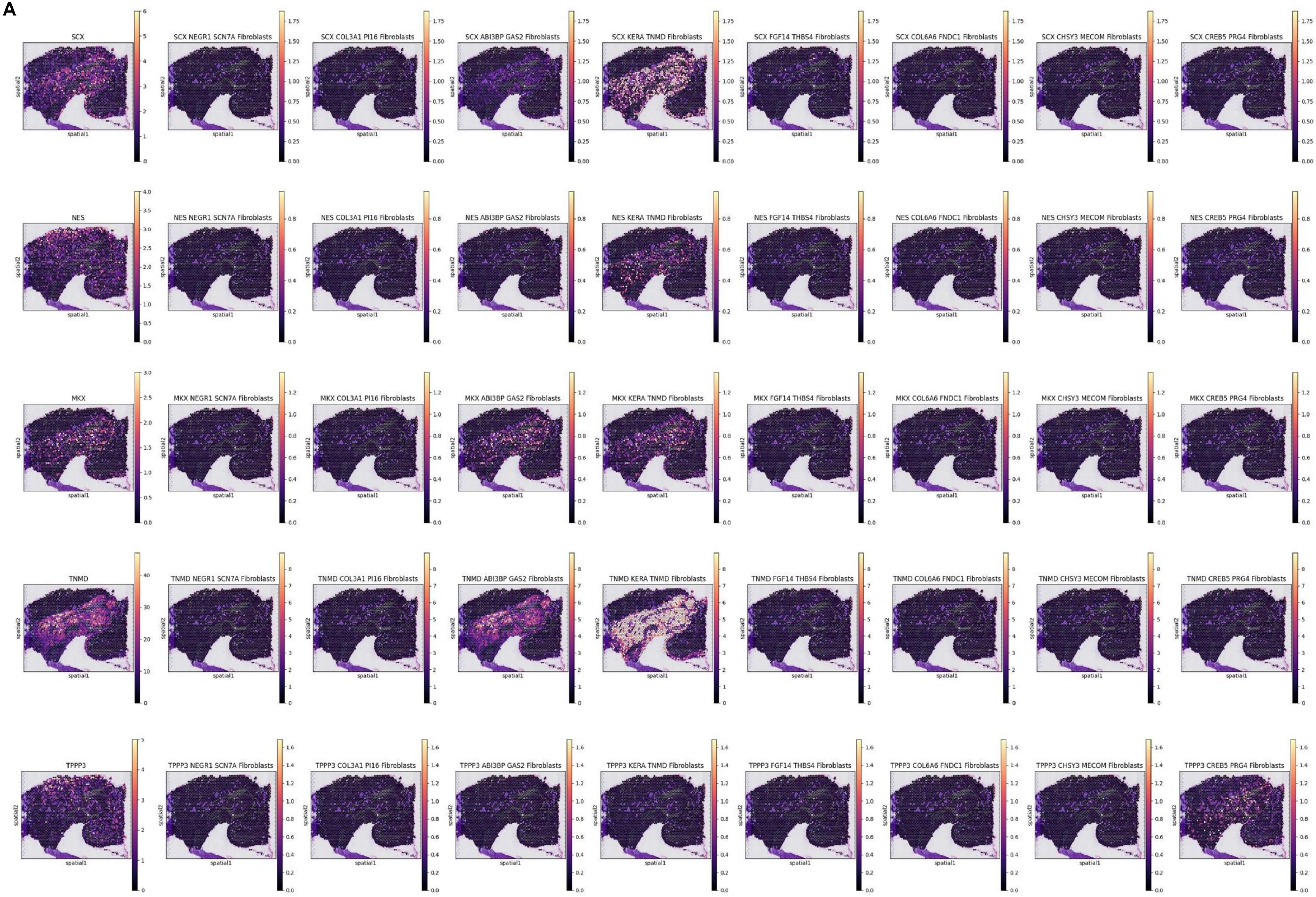
SCX
SCX NEGR1 SCN7A Fibroblasts
SCX COL3A1 PI16 Fibroblasts
SCX ABI3BP GAS2 Fibroblasts
SCX KERA TNMD Fibroblasts
SCX FGF14 THBS4 Fibroblasts
SCX COL6A6 FNDC1 Fibroblasts
SCX CHSY3 MECOM Fibroblasts
SCX CREB5 PRG4 Fibroblasts
NES
NES NEGR1 SCN7A Fibroblasts
NES COL3A1 PI16 Fibroblasts
NES ABI3BP GAS2 Fibroblasts
NES KERA TNMD Fibroblasts
NES FGF14 THBS4 Fibroblasts
NES COL6A6 FNDC1 Fibroblasts
NES CHSY3 MECOM Fibroblasts
NES CREB5 PRG4 Fibroblasts
MKX
MKX NEGR1 SCN7A Fibroblasts
MKX COL3A1 PI16 Fibroblasts
MKX ABI3BP GAS2 Fibroblasts
MKX KERA TNMD Fibroblasts
MKX FGF14 THBS4 Fibroblasts
MKX COL6A6 FNDC1 Fibroblasts
MKX CHSY3 MECOM Fibroblasts
MKX CREB5 PRG4 Fibroblasts
TNMD
TNMD NEGR1 SCN7A Fibroblasts
TNMD COL3A1 PI16 Fibroblasts
TNMD ABI3BP GAS2 Fibroblasts
TNMD KERA TNMD Fibroblasts
TNMD FGF14 THBS4 Fibroblasts
TNMD COL6A6 FNDC1 Fibroblasts
TNMD CHSY3 MECOM Fibroblasts
TNMD CREB5 PRG4 Fibroblasts
TPPP3
TPPP3 NEGR1 SCN7A Fibroblasts
TPPP3 COL3A1 PI16 Fibroblasts
TPPP3 ABI3BP GAS2 Fibroblasts
TPPP3 KERA TNMD Fibroblasts
TPPP3 FGF14 THBS4 Fibroblasts
TPPP3 COL6A6 FNDC1 Fibroblasts
TPPP3 CHSY3 MECOM Fibroblasts
TPPP3 CREB5 PRG4 Fibroblasts
spatial1
spatial2

B

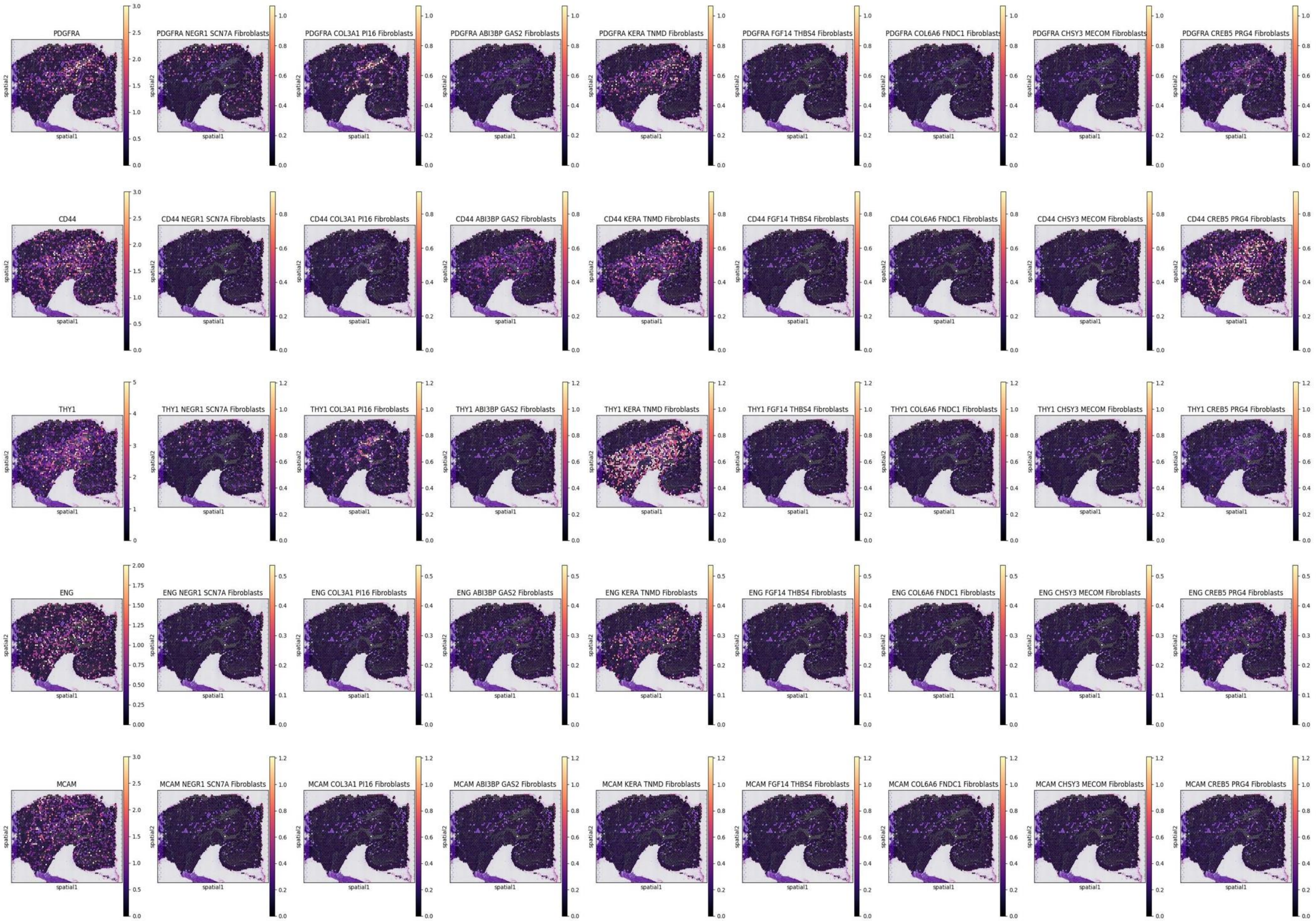
PDGFRA
PDGFRA NEGR1 SCN7A Fibroblasts
PDGFRA COL3A1 PI16 Fibroblasts
PDGFRA ABI3BP GAS2 Fibroblasts
PDGFRA KERA TNMD Fibroblasts
PDGFRA FGF14 THBS4 Fibroblasts
PDGFRA COL6A6 FNDC1 Fibroblasts
PDGFRA CHSY3 MECOM Fibroblasts
PDGFRA CREB5 PRG4 Fibroblasts
CD44
CD44 NEGR1 SCN7A Fibroblasts
CD44 COL3A1 PI16 Fibroblasts
CD44 ABI3BP GAS2 Fibroblasts
CD44 KERA TNMD Fibroblasts
CD44 FGF14 THBS4 Fibroblasts
CD44 COL6A6 FNDC1 Fibroblasts
CD44 CHSY3 MECOM Fibroblasts
CD44 CREB5 PRG4 Fibroblasts
THY1
THY1 NEGR1 SCN7A Fibroblasts
THY1 COL3A1 PI16 Fibroblasts
THY1 ABI3BP GAS2 Fibroblasts
THY1 KERA TNMD Fibroblasts
THY1 FGF14 THBS4 Fibroblasts
THY1 COL6A6 FNDC1 Fibroblasts
THY1 CHSY3 MECOM Fibroblasts
THY1 CREB5 PRG4 Fibroblasts
ENG
ENG NEGR1 SCN7A Fibroblasts
ENG COL3A1 PI16 Fibroblasts
ENG ABI3BP GAS2 Fibroblasts
ENG KERA TNMD Fibroblasts
ENG FGF14 THBS4 Fibroblasts
ENG COL6A6 FNDC1 Fibroblasts
ENG CHSY3 MECOM Fibroblasts
ENG CREB5 PRG4 Fibroblasts
MCAM
MCAM NEGR1 SCN7A Fibroblasts
MCAM COL3A1 PI16 Fibroblasts
MCAM ABI3BP GAS2 Fibroblasts
MCAM KERA TNMD Fibroblasts
MCAM FGF14 THBS4 Fibroblasts
MCAM COL6A6 FNDC1 Fibroblasts
MCAM CHSY3 MECOM Fibroblasts
MCAM CREB5 PRG4 Fibroblasts
spatial1
spatial2

Data S1. Fibroblast type-specific expression of fascicular and sheath TSPC markers.

(A, B) Visium spatial RNA-seq plots show normalised gene expression scores of selected markers across all cells (left column) and within select cell types (all other columns).

A

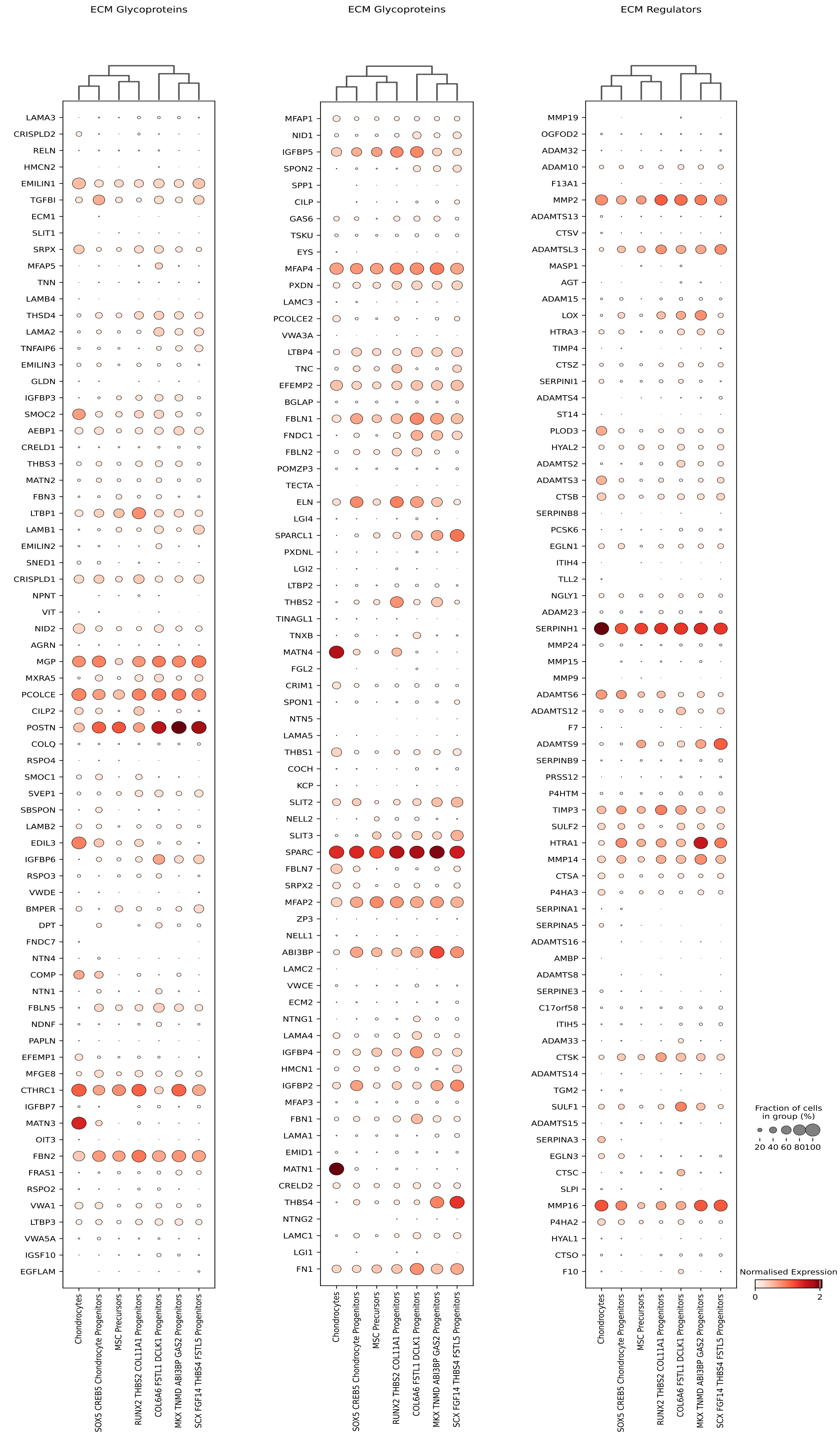
ECM Glycoproteins
ECM Glycoproteins
ECM Regulators
Fraction of cells in group (%)
20 40 60 80 100
Normalised Expression
0
2
Chondrocytes
SOX5 CREB5 Chondrocyte Progenitors
MSC Precursors
RUNX2 THBS2 COL11A1 Progenitors
COL6A6 FSTL1 DCLK1 Progenitors
MKX TNMD ABI3BP GAS2 Progenitors
SCX FGF14 THBS4 FSTL5 Progenitors

B

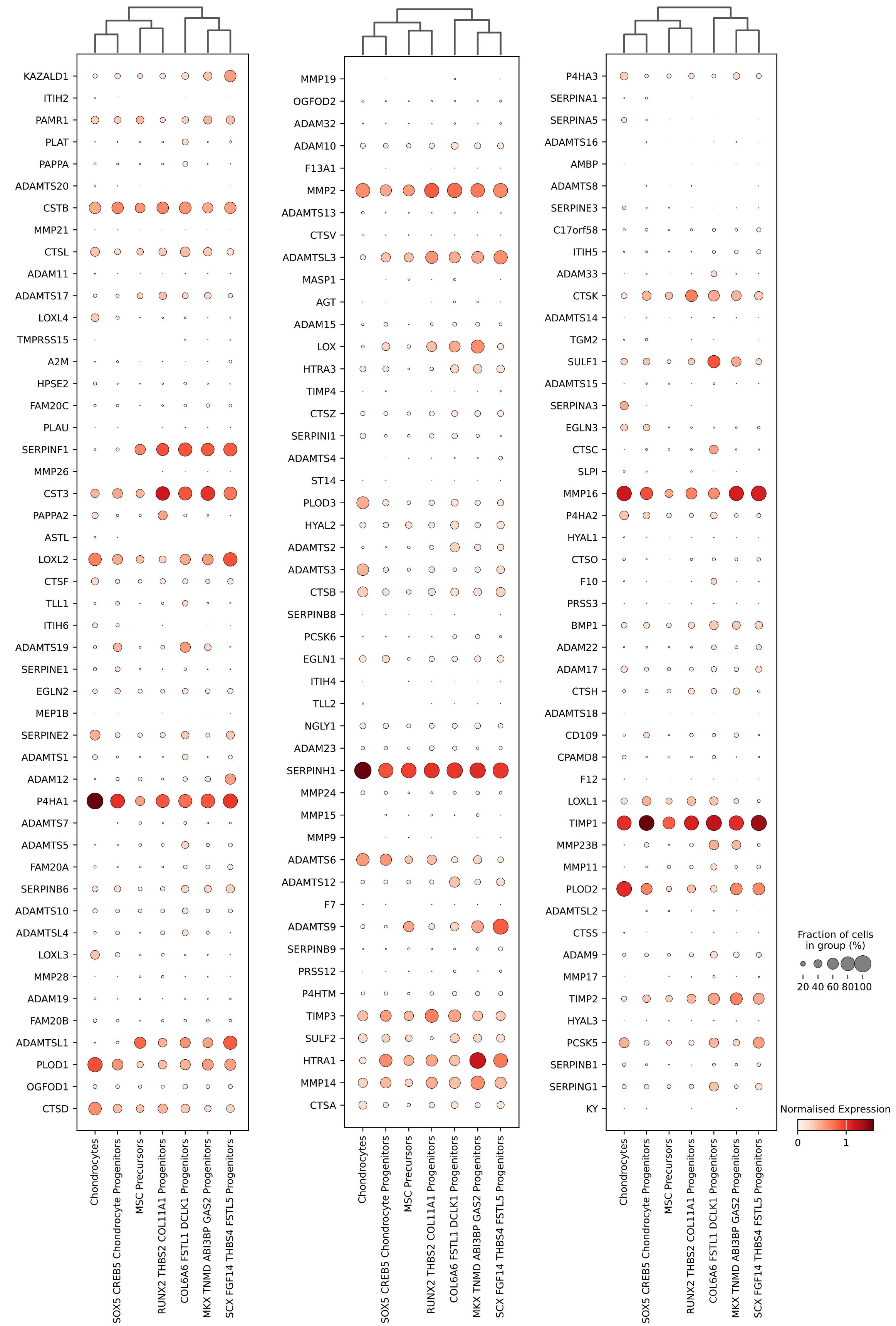
ECM Regulators
ECM Regulators
ECM Regulators
KAZALD1
ITIH2
PAMR1
PLAT
PAPPA
ADAMTS20
CSTB
MMP21
CTSL
ADAM11
ADAMTS17
LOXL4
TMPRSS15
A2M
HPSE2
FAM20C
PLAU
SERPINF1
MMP26
CST3
PAPPA2
ASTL
LOXL2
CTSF
TLL1
ITIH6
ADAMTS19
SERPINE1
EGLN2
MEP1B
SERPINE2
ADAMTS1
ADAM12
P4HA1
ADAMTS7
ADAMTS5
FAM20A
SERPINB6
ADAMTS10
ADAMTSL4
LOXL3
MMP28
ADAM19
FAM20B
ADAMTSL1
PLOD1
OGFOD1
CTSD
MMP19
OGFOD2
ADAM32
ADAM10
F13A1
MMP2
ADAMTS13
CTSV
ADAMTSL3
MASP1
AGT
ADAM15
LOX
HTRA3
TIMP4
CTSZ
SERPINI1
ADAMTS4
ST14
PLOD3
HYAL2
ADAMTS2
ADAMTS3
CTSB
SERPINB8
PCSK6
EGLN1
ITIH4
TLL2
NGLY1
ADAM23
SERPINH1
MMP24
MMP15
MMP9
ADAMTS6
ADAMTS12
F7
ADAMTS9
SERPINB9
PRSS12
P4HTM
TIMP3
SULF2
HTRA1
MMP14
CTSA
P4HA3
SERPINA1
SERPINA5
ADAMTS16
AMBP
ADAMTS8
SERPINE3
C17orf58
ITIH5
ADAM33
CTSK
ADAMTS14
TGM2
SULF1
ADAMTS15
SERPINA3
EGLN3
CTSC
SLPI
MMP16
P4HA2
HYAL1
CTSO
F10
PRSS3
BMP1
ADAM22
ADAM17
CTSH
ADAMTS18
CD109
CPAMD8
F12
LOXL1
TIMP1
MMP23B
MMP11
PLOD2
ADAMTSL2
CTSS
ADAM9
MMP17
TIMP2
HYAL3
PCSK5
SERPINB1
SERPING1
KY
Chondrocytes
SOX5 CREB5 Chondrocyte Progenitors
MSC Precursors
RUNX2 THBS2 COL11A1 Progenitors
COL6A6 FSTL1 DCLK1 Progenitors
MKX TNMD ABI3BP GAS2 Progenitors
SCX FGF14 THBS4 FSTL5 Progenitors
Fraction of cells in group (%)
20 40 60 80 100
Normalised Expression
0
1

C

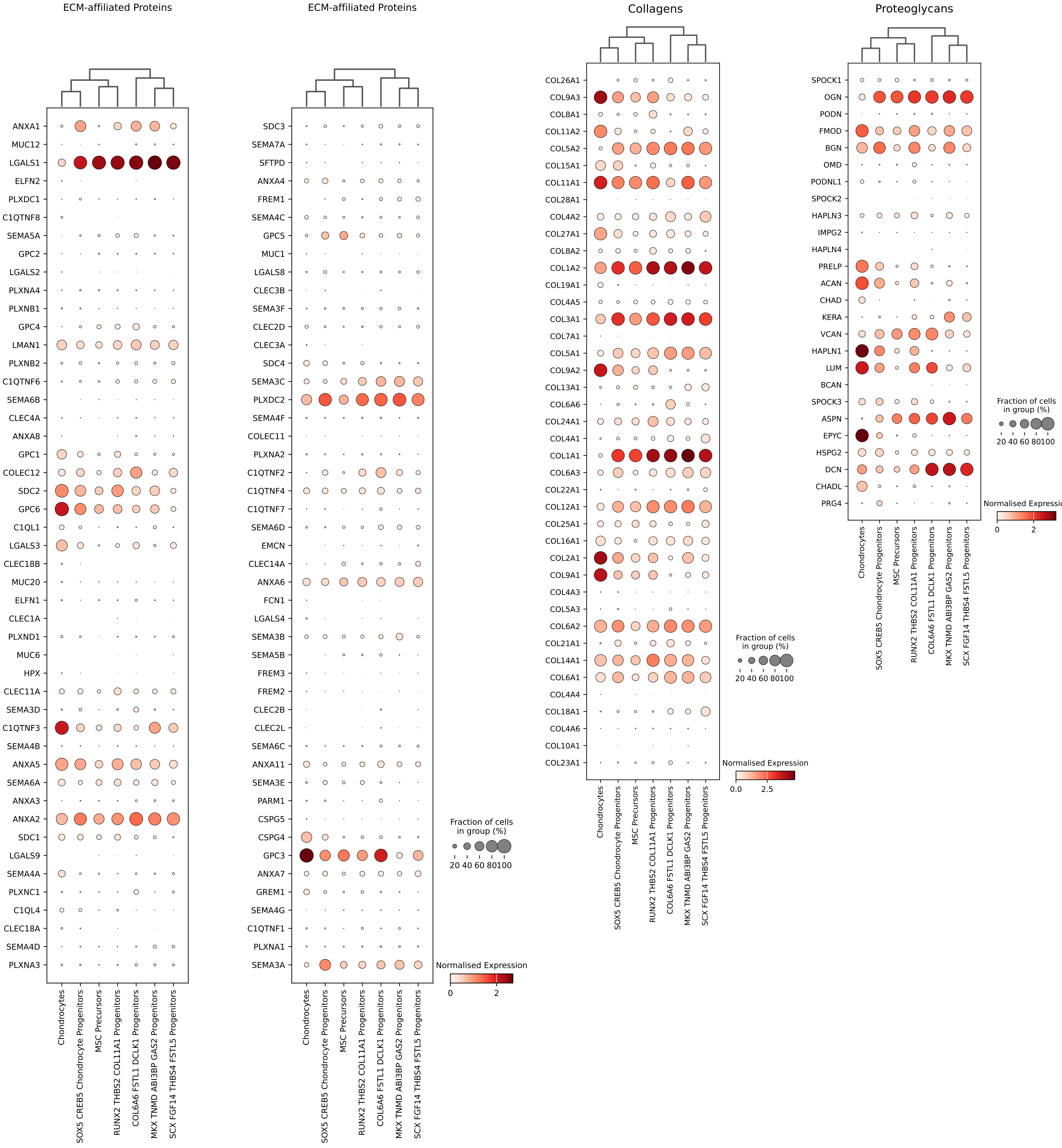

Data S2. Embryonic 6-9pcw tendon matrisomal gene expression profiles.

(A-C) Dotplots show mean normalised expression levels alongside the proportions of expressing cell types for matrisomal genes categorised as ECM-affiliated proteins, ECM glycoproteins, and ECM regulators. The lists of matrisomal genes were obtained from the matrisomeDB database (Shao et al., 2020).
