# Supplementary material for: Cellular and molecular landscapes of human tendons across the lifespan revealed by spatial and single-cell transcriptomics
Source: Cell Rep. 2026 Mar 20;45(4):117085. doi: 10.1016/j.celrep.2026.117085 (PMC13328346; doi:10.1016/j.celrep.2026.117085)
Supplement: Document S2. Article plus supplemental information [file mmc5.pdf]

Cell Reports

Resource

Cellular and molecular landscapes of human tendons across the lifespan revealed by spatial and single-cell transcriptomics

Graphical abstract

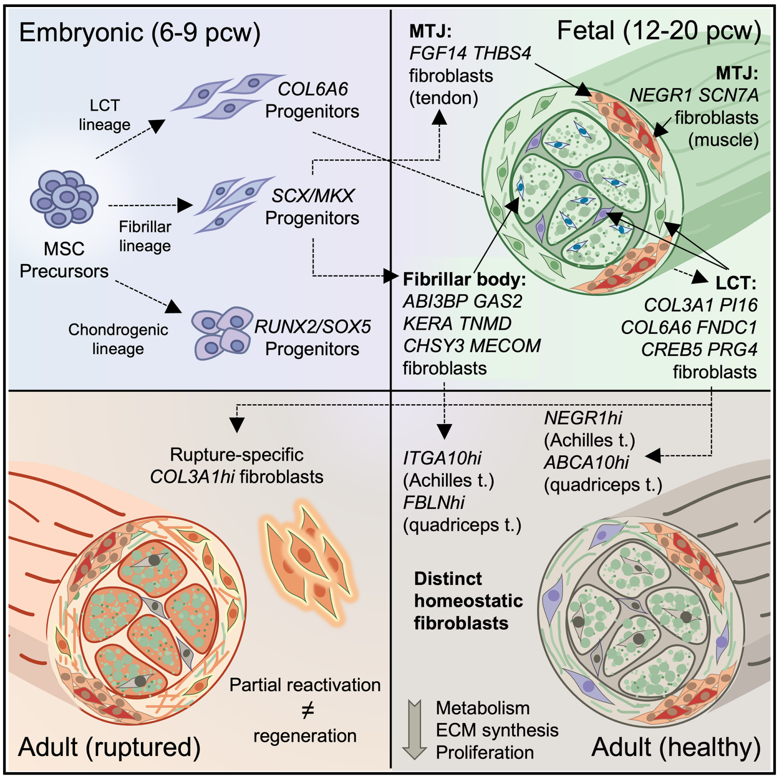

Highlights

- Embryonic progenitors generate fibrillar, connective, and chondrogenic tendon lineages
- Fibroblasts undergo profound transcriptional reprogramming from fetus to adult
- Non-fibroblast populations remain more transcriptionally stable across the lifespan
- Ruptured tendons partially reactivate fetal programs without full regeneration

Authors

Alina Kurjan, Jolet Y. Mimpen, Lorenzo Ramos-Mucci, ..., Adam P. Cribbs, Mathew J. Baldwin, Sarah J.B. Snelling

Correspondence

sarah.snelling@ndorms.ox.ac.uk

In brief

Kurjan et al. map human tendon architecture from embryo to adult using spatial and single-cell transcriptomics. They show embryonic progenitors generate fibrillar, connective, and chondrogenic tendon lineages. Fibroblasts reprogram with age, whereas immune, vascular, and neural cells remain stable. Ruptured adult tendons partially reactivate fetal programs without full regeneration.

Kurjan et al., 2026, Cell Reports *45*, 117085
April 28, 2026 © 2026 The Authors. Published by Elsevier Inc.
https://doi.org/10.1016/j.celrep.2026.117085

CellPress

Cell Reports

CellPress
OPEN ACCESS

Resource

Cellular and molecular landscapes of human tendons across the lifespan revealed by spatial and single-cell transcriptomics

Alina Kurjan,[1] Jolet Y. Mimpen,[1,2] Lorenzo Ramos-Mucci,[1] Ali C. Aksu,[1] Carla J. Cohen,[1,3] Mate Naszai,[4] Christopher D. Buckley,[2] Adam P. Cribbs,[1] Mathew J. Baldwin,[1,5] and Sarah J.B. Snelling[1,5,6,*]
[1]Botnar Institute of Musculoskeletal Sciences, Nuffield Department of Orthopaedics Rheumatology and Musculoskeletal Sciences, University of Oxford, Oxford, UK
[2]Kennedy Institute of Rheumatology, Nuffield Department of Orthopaedics Rheumatology and Musculoskeletal Sciences, University of Oxford, Oxford, UK
[3]Centre for Computational Biology, MRC Weatherall Institute of Molecular Medicine, University of Oxford, Oxford, UK
[4]Southmead Hospital, North Bristol NHS Trust, Bristol, UK
[5]Senior author
[6]Lead contact
*Correspondence: sarah.snelling@ndorms.ox.ac.uk
https://doi.org/10.1016/j.celrep.2026.117085

SUMMARY

Tendon injuries are common and heal poorly, whereas developing tendons repair with minimal scarring; how this capacity declines with age remains poorly understood. Here, we combine histology, single-nucleus, single-cell, and spatial transcriptomic profiling of human Achilles and quadriceps tendons across embryonic, fetal, and adult stages, including ruptured adult tendons. We identify seven embryonic progenitor states that are predicted to contribute to three tendon-associated lineages—fibrillar, connective tissue, and chondrogenic—which diversify over development, occupy discrete spatial niches, and appear to acquire specialized roles in matrix synthesis, remodeling, and mechanical adaptation. While non-fibroblast populations remain transcriptionally stable with age, fibroblasts undergo marked reprogramming, shifting to homeostatic or injury-responsive states. In ruptured adult tendons, a subset of fibroblasts partially reactivates developmental programs yet remains transcriptionally distinct from developmental states that exhibit scarless healing. These findings define the cellular architecture of human tendon development and aging and reveal lineage-specific targets for therapeutic repair.

INTRODUCTION

Tendons are essential connective tissues that transmit force between muscles and bones, enabling movement and stabilizing joints. Despite their remarkable strength and resilience, tendons are vulnerable to injuries and degenerative conditions (often termed tendinopathies), which cause pain, swelling, and impaired function, severely affecting quality of life.[1–4]

The capacity for tendon repair varies drastically across the lifespan. Whereas fetal and neonatal tendons can regenerate with minimal scarring, adult tendons typically heal through fibrosis or ectopic ossification, resulting in disorganized extracellular matrix (ECM) and compromised mechanical properties.[5–10] These contrasting outcomes appear driven by intrinsic differences in tendon-resident cell behavior.[6,8,11–17] That said, scarless healing in fetal and early postnatal tendons co-occurs with a distinct developmental context (immature ECM architecture, growth-related forces, a different immune milieu, etc.) and with mechanical demands that are not yet equivalent to sustained postnatal loading. Experimental studies show that mechanical stimulation is essential for proper tendon formation and maturation: for example, chick and mouse embryos lacking muscle activity develop poorly formed or absent tendons and show impaired joint and musculoskeletal development.[18,19] At the same time, the capacity for scarless repair declines around the period when mechanical loading increases postnatally, coinciding with decreased tendon cell proliferation and the onset of expansive collagen fibril growth.[9,20,21] Therefore, mechanical load is indispensable for tendon development, maturation, and adult healing.

Recent spatial and single-cell (scRNA)/nucleus (snRNA) RNA-sequencing studies have begun to uncover the cellular heterogeneity underlying these differences, though findings vary across species and anatomical locations.[13,22–30] Beyond the well-characterized *COL1A1*$^+$*TNMD*$^+$*MKX*$^+$ intrafascicular tenocytes responsible for ECM production and fiber alignment,[31–34] tendons also harbor fibroblasts with elevated *COL3A1* expression,[24–30,35] *PTPRC*/*CD45*$^+$ immune cells,[13,23–26,28–30] *PECAM1*/*CD31*$^+$ endothelial cells,[13,23–30] *MCAM*/CD146$^+$ pericytes,[23,30] *NOTCH3*$^+$ mural or *ACTA2*$^+$ smooth muscle cells,[24,25,28–30] and, in some studies, muscle cells,[25,28,30] neural cells,[23,28,30] adipocytes,[28,30] and fibro-adipogenic progenitors.[13,24] Despite this

© 2026 The Authors. Published by Elsevier Inc.
This is an open access article under the CC BY license (http://creativecommons.org/licenses/by/4.0/).

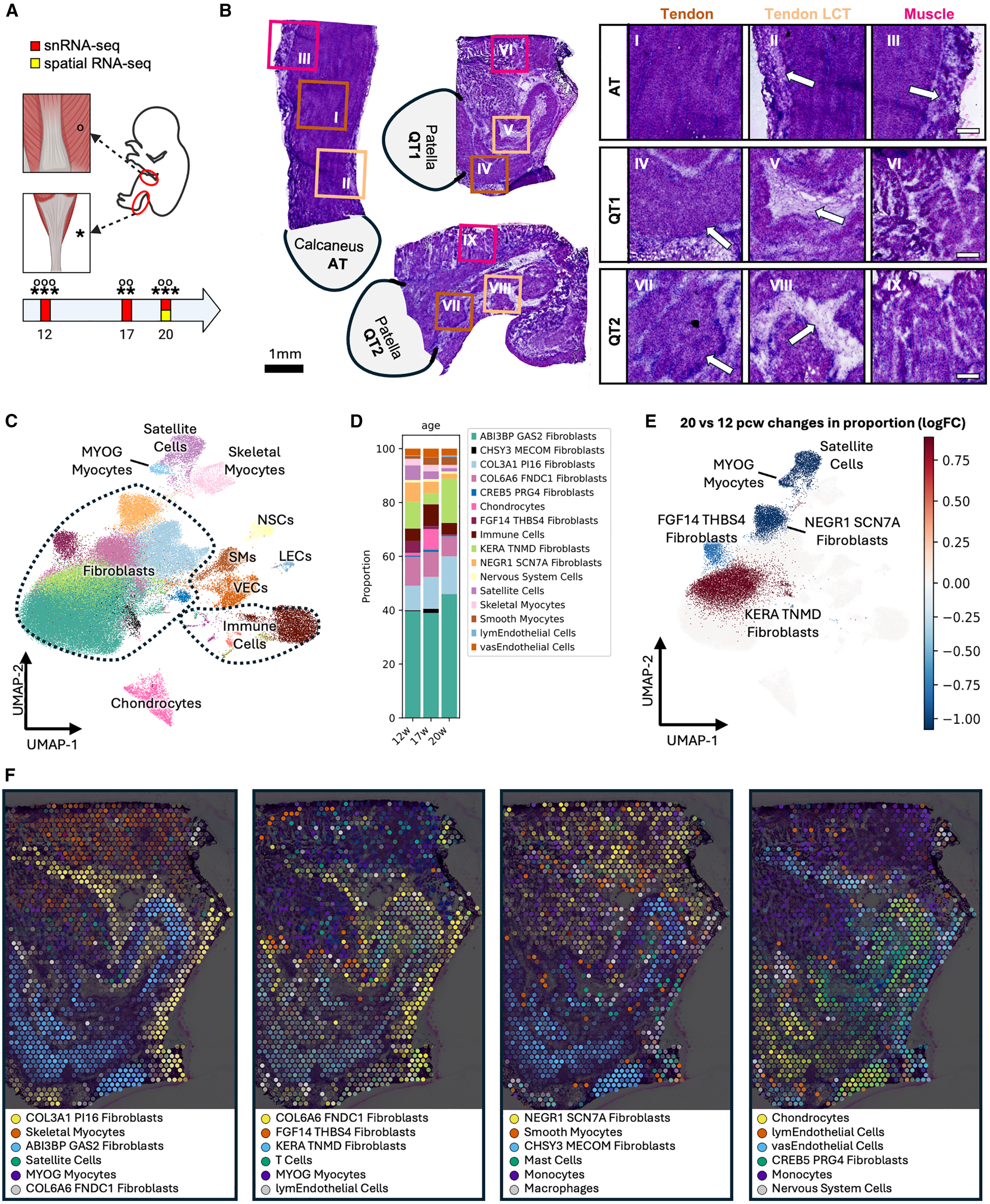

(legend on next page)

emerging complexity, consistent identification of canonical tendon stem/progenitor cells (TSPCs)–marked by *STRO-1*, *MCAM/CD146*, *ENG/CD105*, *THY1/CD90*, *CD44*, *SCX*, *TNMD*, *COMP*, and *TNC*[36,37]–remains elusive. Instead, recent studies describe injury-responsive, sheath-derived progenitors expressing *SCX*$^{-}$*TPPP3*$^{+}$*PDGFRA*$^{+}$ [13,38] or *AXIN2*$^{+}$[12,17] signatures in adult tendons.

Evidence from murine models highlights that tendon healing is age- and context-dependent. Neonatal tendons exhibit robust regenerative capacity driven by intrinsic *Scx*$^{+}$ cells,[9] while adult tendons predominantly heal through fibrotic mechanisms, involving extrinsic *Scx*$^{-}$*Acta2*$^{+}$[9] or *Sca1*$^{+}$[39] cells. Mechanical load and TGF-β signaling further modulate *Scx* expression and ECM organization, shaping whether repair proceeds via regenerative or fibrotic pathways.[9,12,20,39–48] Yet, major gaps remain regarding how tendon cell identity, plasticity, and niche-specific behaviors evolve over time from development into adulthood, particularly following injury. Moreover, whether developmental tendon lineages are retained, reactivated, or replaced after rupture in adult humans is not yet understood.

Here, we map the transcriptional landscapes of human tendons across development and aging. Using snRNA-, scRNA-, and spatial RNA sequencing, we profile cell populations within embryonic (6–9 post-conception weeks [pcw]), fetal (12–20 pcw), and adult (25–76 years) human tendons. We further characterize how rupture remodels the cellular composition and cell state in adult quadriceps tendons. This study identifies conserved and divergent fibroblast populations across life stages, revealing signatures of developing fibroblasts and defining how aging and injury reshape tendon cell fate and function.

RESULTS

To characterize cell populations in intrinsically (re)generative human tendons, we performed snRNA-seq on Achilles (*N* = 8 samples) and quadriceps tendons (*N* = 7 samples) from nine human fetal donors aged 12, 17, and 20 pcw (Figure 1A; Table S1). In parallel, spatial transcriptomics was conducted on Achilles and quadriceps tendons (*N* = 1 and *N* = 2 samples, respectively) from a single 20 pcw donor (Figure 1B). Following data processing and quality control, scVI-integrated[49] snRNA-seq data from 91,859 nuclei were clustered and annotated, identifying distinct fibroblasts, chondrocytes, immune, endothelial, muscle, and nervous system-associated cells (Figure 1C) across the ages (Figure 1D). Cell2location[50] analysis mapped these snRNA-seq cell type signatures to their likely spatial locations within the tissues (Figure 1F).

snRNA- and spatial RNA-sequencing reveal heterogeneous compartmentalized fibroblasts within second trimester fetal tendons

Fibroblasts dominated the cellular landscape of fetal tendons, forming at least eight transcriptional cell states across a few closely related types (Figure 2A). Three populations–termed ABI3BP GAS2, KERA TNMD, and CHSY3 MECOM Fibroblasts–shared transcriptional similarities, indicating a common lineage with functional divergence marked by key gene expression profiles (Figure 2B). The largest, ABI3BP GAS2 Fibroblasts, were defined by high levels of *COL11A1*[51] alongside ECM- and cytoskeletal regulators *ABI3BP*, *GAS2*, *SOX5*, *EXT1*, and *PLEKHH2*, consistent with roles in matrix assembly and cellular adhesion (Figures 2B and 2C). Transcriptionally similar to these, KERA TNMD Fibroblasts were enriched for collagens (*COL1A1*, *COL1A2*, *COL6A3*, *COL6A1*, and *COL12A1*) and matrix-associated genes (*SPARC*, *POSTN*, *FMOD*, and *KERA*), with comparatively lower *MKX* and higher *TNMD* expression (Figures 2B and 2C) than in other fetal fibroblast states, consistent with a more matrix-producing differentiated fibroblast phenotype.[52–58] CHSY3 MECOM Fibroblasts, in contrast, expressed ECM-hydration regulator *CHSY3*, transcriptional regulators *MECOM* and *FOXP2*, and mineralization-associated *COL24A1,*[59] *SMOC1*,[60] and *ENPP1*,[61] as well as chondrogenic and enthesis markers *SOX6*[30] and *COL27A1*[31,62,63] (Figure 2B), respectively, suggesting a role in tendon-bone interface remodeling and mechanoadaptation.

Spatial transcriptomic analysis localized these three transcriptionally related fibroblast populations within the main fascicular bodies of 20 pcw Achilles and quadriceps tendons (Figure 1F; 2E-Factor 0). High expression of canonical TSPC markers *ENG*, *THY1*, *CD44*, and *NES*[36] was specifically enriched within spatially resolved KERA TNMD Fibroblasts (Data S1).

Temporal pseudobulk differential gene expression (DGE) analysis comparing 20 and 12 pcw tendons, followed by Gene Ontology Biological Process (GO:BP) enrichment, revealed that by 20 pcw, ABI3BP GAS2, KERA TNMD, and CHSY3 MECOM fibroblasts upregulate genes involved in cell growth, metabolism, pattern formation, Wnt signaling regulation, histone methylation, and stem cell differentiation (Table S2). These populations also showed increased expression of genes linked to immune system modulation while downregulating cell division and contractile programs, including those related to muscle

Figure 1. Experimental design, histological and spatial profiling, and compositional shifts in developing human tendons
(A) Overview of experimental design. Stars and circles indicate the numbers of Achilles and quadriceps tendons analyzed per timepoint, respectively. Tendon and fetal icons were created with BioRender.com.
(B) H&E-stained cryosections (10 μm) of Achilles tendon (AT) and two quadriceps tendons (QT1 and QT2) used for 10X Visium spatial RNA seq. Tissue orientation is annotated by adjacent bone structures (calcaneus and patella). Black scale bar, 1 mm. Insets show zoomed regions (white boxes) with anatomical features annotated: tendon body, loose connective tissue (LCT), and muscle. White scale bars, 250 μm.
(C) UMAP of scANVI-integrated 12–20 post-conception week (pcw) fetal tendon snRNA-seq data, showing annotated cell types. VECs: vascular endothelial cells; LECs: lymphatic endothelial cells; SMs: smooth myocytes; NSCs: nervous system-associated cells.
(D) Bar plot of cell type proportions by fetal age.
(E) UMAP showing scCODA-inferred compositional changes from 12 to 20 pcw. Color denotes $\log_2$ fold-change (effect size) for cell type proportions, with red corresponding to increases and blue to decreases in proportions. Only statistically significant changes (FDR < 0.05) are shown.
(F) Cell2location spatial mapping of 20 pcw quadriceps tendon sections, showing relative abundance of each cell type.

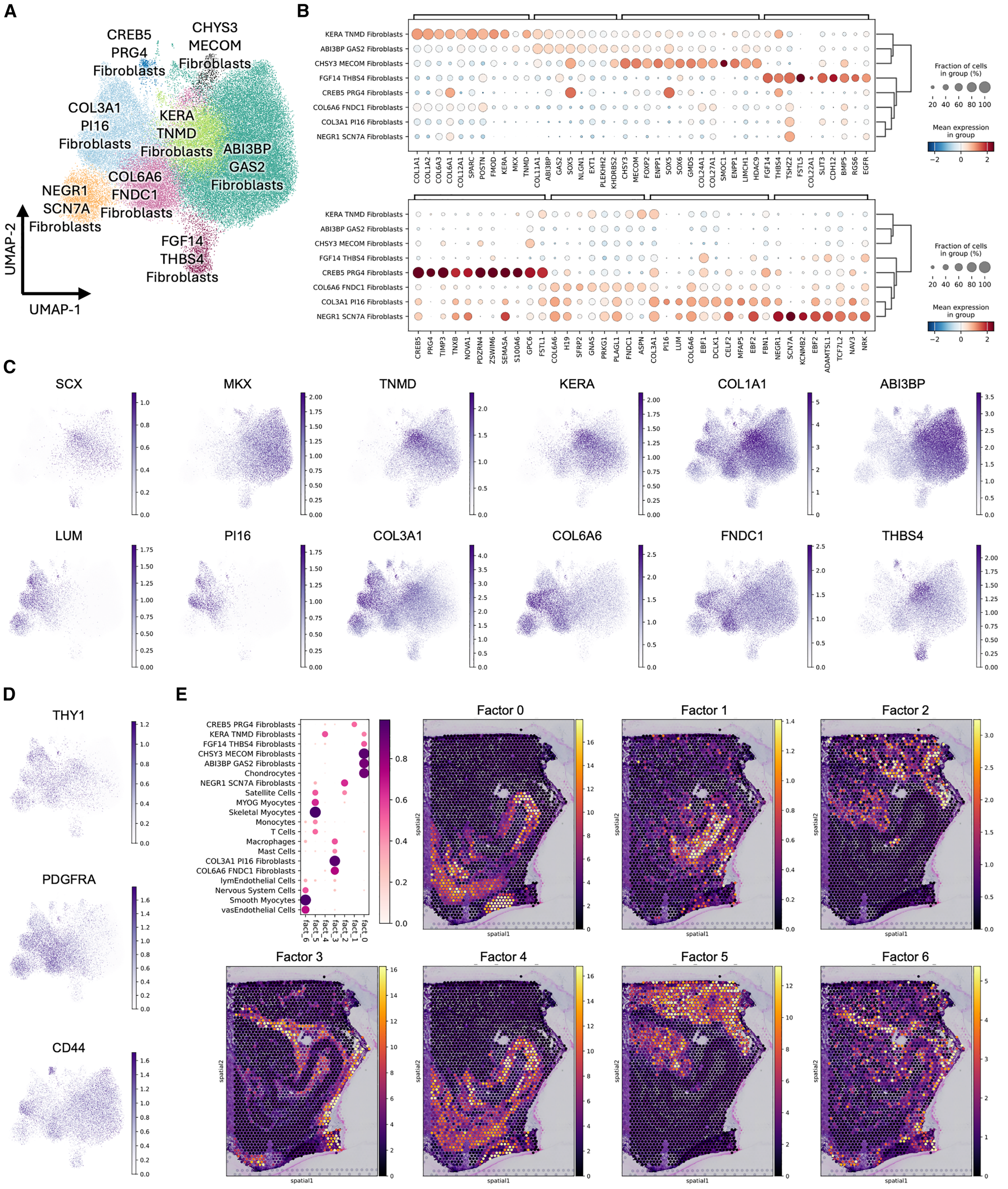

Figure 2. Transcriptional diversity, marker expression, and spatial localization of fibroblast subtypes in fetal human tendons

(A) UMAP of annotated fibroblast subtypes from 12 to 20 post-conception week (pcw) fetal tendon snRNA-seq data.

(B) Dot plots of log1pPF-normalized and scaled gene expression showing hierarchically clustered, differentially expressed genes across fibroblast subtypes. Dot size indicates cell type abundance.

(legend continued on next page)

contraction and myofibril assembly. These findings suggest that these fetal fibroblasts not only promote tendon matrix formation but also help modulate the local immune environment and suppress contractile differentiation during tendon development.

A second major group of transcriptionally related fibroblast states was characterized by high expression of *COL3A1*, *COL6A6*, *PDGFRA*, *DCLK1*, *TSHZ2*, *PLAGL1*, and *VCAN* and comprised three dominant subtypes: COL3A1 PI16, COL6A6 FNDC1, and NEGR SCN7A Fibroblasts (Figure 2B). The most abundant, COL3A1 PI16 Fibroblasts, exhibited high expression of *COL3A1*, *PI16*, *EBF1*, *DCLK1*, *LUM*, *MFAP5*, and *FBN1* (Figures 2B and 2C) and localized to the loose connective tissue (LCT) regions of Achilles and quadriceps tendons (Figures 1F and 2E). These cells also expressed sheath TSPC markers *TPPP3* and *PDGFRA*[13,38] (Data S1). From 12 to 20 pcw, they upregulated pathways related to Hippo signaling, chondrocyte and epithelial development, and suppression of vascular smooth muscle proliferation while downregulating programs associated with muscle development, stress responses, apoptosis, and epigenetic modification (Table S2). These features suggest a role in early ECM organization and structural maintenance within the tendon LCT.

A transcriptionally similar population, COL6A6 FNDC1 Fibroblasts, was distinguished by elevated expression of *COL6A6*, *FNDC1*, and regulators of chondrogenesis and mineralization *SFRP2*,[64] *GNAS*,[65] and *ASPN*[66–68] (Figure 2B). Like COL3A1 PI16 Fibroblasts, they resided within the LCT regions (Figures 1F and 2E). Between 12 and 20 pcw, they upregulated pathways involved in tissue repair while downregulating processes linked to cell adhesion, miRNA regulation, synaptic function, metabolism, and vascular development (Table S2). These signatures point to a role in maintaining tendon structural integrity and modulating immune responses during growth.

NEGR1 SCN7A Fibroblasts were characterized by high expression of *NEGR1*, *SCN7A*, *EBF2*, *NRK*, and *KCNMB2*, suggesting involvement in fibroblast mechanosensitivity. They also expressed *NAV3* and *ADAMTSL1*, linked to cytoskeletal and ECM organization, respectively (Figure 2B). Spatial mapping located these cells between skeletal myocytes within tendon-adjacent muscle LCT regions (Figures 1F, 2E, and 3E), implicating them in myotendinous junction (MTJ) organization, mechanotransduction, and matrix remodeling.

Another distinct fibroblast population, FGF14 THBS4 Fibroblasts, occupied the tendon-muscle boundaries (Figure 1F) and expressed MTJ-associated genes *COL22A1*[69,70] and *THBS4*, alongside high levels of *FGF14*, *SLIT3*, *TSHZ2*, *CDH12*, *BMP5*, *RGS6*, *EGFR*, and *FSTL5* (Figures 2B and 2C). From 12 to 20 pcw, these cells upregulated pathways involved in ECM synthesis, angiogenesis, immune interactions, and neurogenesis, while downregulating BMP signaling, ossification, and skeletal muscle proliferation pathways (Table S2), supporting a role in maintaining a specialized MTJ-supporting fibroblast identity.

Finally, a small CREB5 PRG4 Fibroblast population localized within LCT regions (Figures 1F and 2E) expressed synovium- and cartilage-associated *CREB5*,[71,72] alongside lubricating *PRG4*.[73–78] These cells also showed high levels of *TIMP3*, *TNXB*, and *NOVA1* (Figure 2B), suggesting roles in ECM maintenance, collagen organization, and tendon lubrication, particularly in regions subject to mechanical stress.

Vascular, immune, and neural niches emerge alongside fibroblasts to structure developing human tendons

Non-fibroblast populations identified in fetal tendons included vascular and lymphatic endothelial cells, smooth muscle cells, nervous system-associated cells, immune cells, and myocytes (Figure 3A). Vascular endothelial cells expressed *PECAM1*, *CD34*, and *VWF*, while lymphatic endothelial cells were marked by *LYVE1*, *PROX1*, and *FLT4* (Figures 3B and 3C). Smooth muscle cells showed high expression of *ACTA2*, *MYH11*, *CALD1*, *NOTCH3*, and *PDGFRB* (Figure 3B). Nervous system-associated cells were distinguished by *NRXN1*, *NCAM2*, and *SOX10* expression (Figure 3B). All these cell types co-localized within the tendon LCT regions (Figures 1F, 2E, and 3E).

Immune cells broadly expressed *PTPRC*, *CD44*, *IKZF1*, *RUNX1*, *DOCK2*, and *INPP5D*. The predominant subset expressed *F13A1*, *CD163*, *MRC1* (*CD206*), *CSF1R*, *CD36*, and *LGMN*, consistent with macrophage identity (Figure 3B). Additional immune subsets included monocytes (*FCN1*, *LYZ*, and *IRAK3*), mast cells (*KIT*, *CPA3*, and *GATA2*), and T cells (*SKAP1*, *CD247*, *CD96*, *CD38*; Figure 3B). While monocytes and T cells were predominantly co-localized with myocytes in muscle regions, mast cells and macrophages were enriched within the tendon LCT, co-localizing with COL3A1 PI16 and COL6A6 FNDC1 fibroblasts (Figure 2E-Factors 3,5).

Three myocyte clusters were identified within muscle tissue adjoining the tendon MTJ (Figures 1F, 2E, and 3E). One cluster expressed *PAX7*, marking satellite cells, while a second expressed markers of differentiated skeletal myocytes including *DES*, *NES*, *TNNT3*, *TTN*, *MYH3*, *COL22A1*, and *TNNC1* (Figures 3B–3D). A third small cluster, expressing *MYOG*, *FNDC5*, *EMC10*, *MEGF10*, and overlapping markers from both satellite and skeletal myocytes, likely represented a transitional state (Figures 3B–3D).

Finally, in one 17pcw quadriceps tendon sample, a discrete population expressing *COL2A1*, *COL9A1*, *ACAN*, *COMP*, *MATN1*, and *HAPLN1* was detected, consistent with a chondrocyte identity (Figure 3B). These cells co-localized with the fascicular fibroblasts within the main fibrillar tendon body (Figure 1F, 2E-Factor0), likely reflecting minor contamination from adjacent patellar cartilage.

(C) UMAPs showing normalized expression of selected fibroblast marker genes across annotated subtypes.
(D) UMAPs showing expression of selected tendon stem/progenitor cell (TSPC) markers: THY1 (CD90), PDGFRA, and CD44.
(E) Unsupervised non-negative matrix factorization (NMF) analysis of spatial transcriptomics cell2location output across three 20 pcw tendon samples. Dot plot displays NMF factors derived from mean normalized UMI counts per factor (dot size and color reflect gene expression and loading strength). Gene loadings indicate the contribution of individual genes to each colocalized fibroblast factor. Spatial scatterplots show cell densities (mean normalized UMI counts) per NMF factor from one representative 20 pcw quadriceps tendon section.

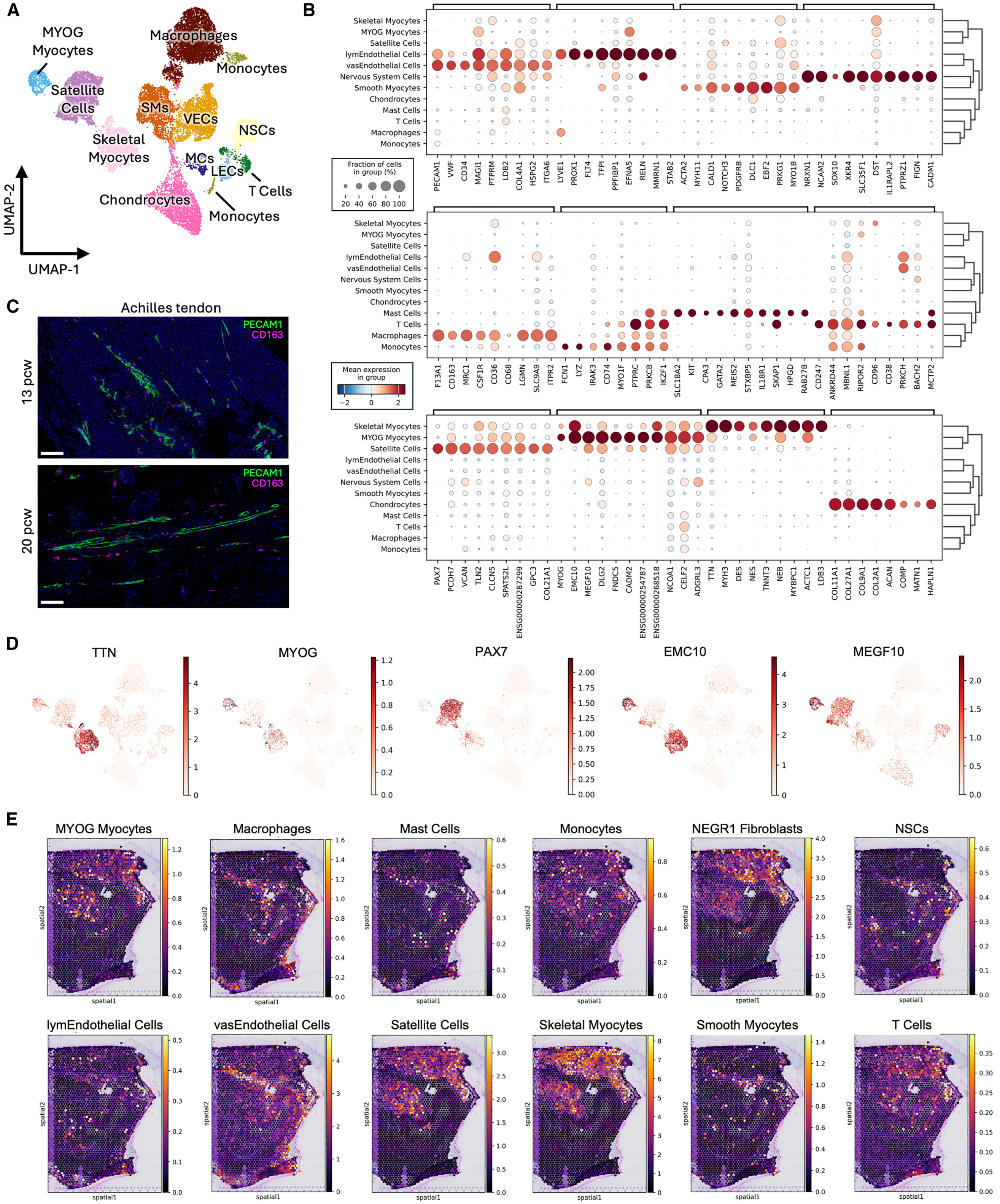

(legend on next page)

Second trimester fetal tendon populations shift from MTJ- and muscle-associated to tendon-building cells

To quantify changes in cell type proportions over time, we applied single-cell compositional data analysis (scCODA),[79] a Bayesian modeling approach that accounts for sampling biases inherent to single-nucleus sequencing. Nervous system-associated cells (NSCs) were automatically designated as the reference group due to their relatively low abundance and stable detection across 12–20 pcw donors. This choice was supported by comparable results when using other low-abundance cell types (e.g., lymphatic endothelial cells and smooth myocytes) as references. At 20 pcw, compositional analysis revealed a significant decrease in MTJ- and muscle-associated populations, including FGF14 THBS4 Fibroblasts, MYOG Myocytes, Satellite Cells, and NEGR1 SCN7A Fibroblasts (Figure 1D). In contrast, KERA TNMD Fibroblasts–resembling differentiated tenocytes and expressing markers of fascicular TSPCs–showed a substantial proportional expansion during this period (Figure 1D).

Single-cell and spatial transcriptomics of embryonic limbs reveal candidate progenitors for fetal tendon fibroblast lineages

The initiation of cell lineage decisions during early embryogenesis is crucial for establishing the cellular diversity required for tendon development. To investigate the origins of fetal tendon populations, we analyzed published spatial and scRNA-sequencing datasets from 6 to 9 pcw human embryonic limbs.[80] Tendon regions were manually delineated within an 8 pcw H&E-stained spatial RNA-seq sample (Figure S1), and gene expression profiles were extracted as a reference for cell identification in the corresponding scRNA-seq dataset. A random forest classifier trained on these profiles identified 4,318 tendon-like cells out of 108,617 total cells (Figure S2). After filtering and batch correction, seven cell state clusters were annotated across 3,092 cells from six donors aged 6.5–9.3 pcw (Figure 4A).

Mesenchymal stem cells (MSCs) were first examined as the likely progenitors of tendon fibroblasts. The earliest state, MSC Precursors, was identified at 6.5–7.2 pcw and lacked classical MSC markers but expressed a combination of non-myogenic (*HMGA2*, *FOXP2*, and *RUNX1T1*)[81,82] and myogenic (*SIX1*)[81,83,84] connective tissue fate regulators. These cells also expressed genes involved in cell structure, adhesion, metabolism, growth, and signaling, alongside low levels of *TNMD*, *TSHZ2*, *GAS2*, and *VCAN* (Figure S3).

By 8.4 pcw, a distinct COL6A6 Progenitor population emerged, expressing canonical MSC markers (*CD73/NT5E*, *CD90/THY1*, *CD44*, and *TWIST2*), along with tendon-associated matrisomal genes (*FSTL1*, *DCLK1*, *COL1A1*, *COL3A1*, *COL5A1*, *COL6A1*, *COL12A1*, *ELN*, *DCN*, *ASPN*, *OGN*, *FNDC1*, *LUM*, *VCAN*, *EMILIN2*, *MFAP5*, *TNXB*, and *POSTN*; Figure 4B; Data S2). This population also expressed LCT-associated transcription factors *OSR1* and *OSR2*,[85,86] suggesting transition toward the *COL6A6*-expressing LCT fibroblast phenotypes observed in second-trimester fetal tendons.

In parallel, three chondrogenic clusters were identified at different stages of differentiation. A multipotent *RUNX2*-expressing population exhibited a hybrid transcriptomic profile, co-expressing collagens linked to tendon (*COL1A1* and *COL3A1*), cartilage (*COL2A1* and *COL9A1–COL9A3*), and endothelial basement membranes (*COL8A1*; Figure 4B). Two more differentiated clusters expressed classic cartilage markers (*SOX5*, *SOX6*, *SOX9*, *ACAN*, *CILP2*, *HAPLN1*, *COMP*, *COL2A1*, and *COL9A1–COL9A3*; Figure 4B; Data S2). The less mature SOX5 Progenitors additionally expressed *CREB5* and *PRG4*, while the more differentiated Chondrocyte cluster was enriched for *MATN1*, *MATN3*, and *MATN4* (Figure 4B).

Finally, two distinct *SCX*-expressing progenitor clusters were identified. SCX Progenitors were characterized by high expression of *FGF14*, *THBS4*, *FSTL5*, *CHODL*, *TSHZ2*, *CDH12*, *KERA*, *POSTN*, *DCN*, *OGN*, and a broad array of fibrillar (*COL1A1*, *COL3A1*, *COL5A1*, *COL6A1*, *COL12A1*, and *COL14A1*), basement membrane (*COL18A1*, *COL4A1*, and *COL4A2*), and MTJ-associated (*COL22A1*) collagens (Figure 4B; Data S2). MKX Progenitors exhibited even higher expression of matrisomal genes, along with *LOX*, *MKX*, *TNMD*, *ABI3BP*, and *GAS2*, marking a more differentiated tenogenic state (Figure 4B; Data S2).

Trajectory and regulatory network analyses reveal emergence of three distinct tendon-associated lineages during human development

To reconstruct the differentiation pathways underpinning tendon development, we applied RNA velocity[87] and pseudotime analysis using Palantir[88] and CellRank[89–91] across embryonic progenitor populations. To link embryonic trajectories to fetal tendon states, we integrated datasets using scGen[92] with batch correction by moscot,[93] enabling continuous reconstruction of lineage emergence from early embryogenesis through the second trimester. Gene regulatory networks were then inferred using SCENIC to identify transcription factors and regulatory programs associated with lineage specification.

RNA velocity analysis indicated that MSC Precursors were predicted to diverge toward four distinct fates: MKX Progenitors, RUNX2 Progenitors, SOX5 Progenitors, and MSC-like COL6A6 Progenitors (Figure 4C). SCX Progenitors also displayed a bias

Figure 3. Characterization and spatial mapping of non-fibroblast cell populations in developing human tendons

(A) UMAP of annotated non-fibroblast cell types from 12 to 20 post-conception week (pcw) fetal tendon snRNA-seq data. VECs: vascular endothelial cells; LECs: lymphatic endothelial cells; SMs: smooth myocytes; NSCs: nervous system-associated cells; MCs: mural cells.

(B) Dot plots of log1pPF-normalized and scaled gene expression showing hierarchically clustered, differentially expressed genes across non-fibroblast cell types. Dot size indicates cell type abundance.

(C) Immunofluorescence images of 20 pcw Achilles tendon sections stained for PECAM1 (green, endothelial cells) and CD163 (magenta, macrophages). White scale bars, 100 μm.

(D) UMAPs showing normalized expression of selected myocyte marker genes.

(E) Cell2location spatial mapping of cell types in 20 pcw quadriceps tendon sections, with color intensity indicating relative abundance at each location.

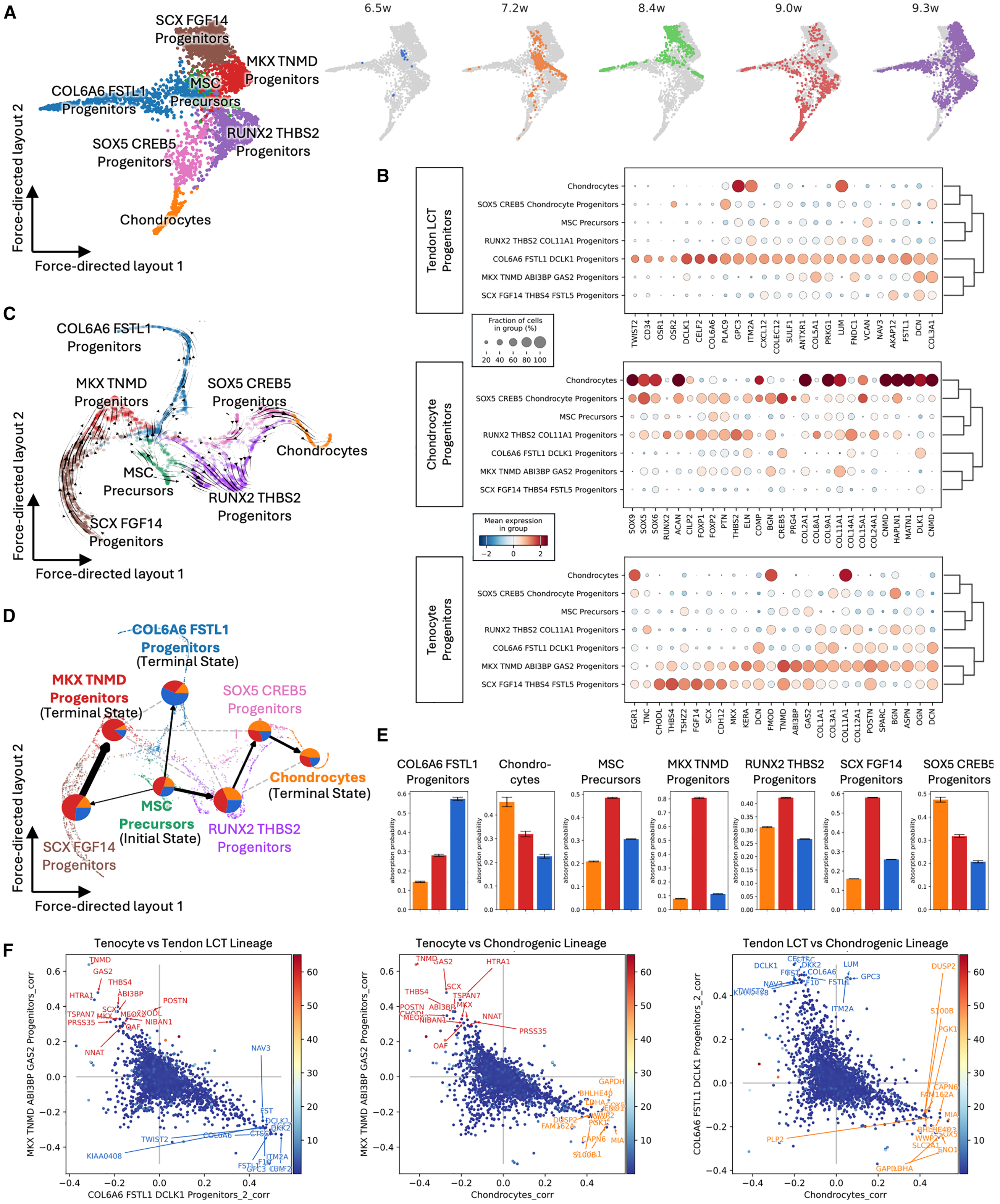

Figure 4. Embryonic progenitor states and lineage trajectories reveal early tendon cell fate specification

(A) Harmony-integrated force-directed layout showing the distribution of embryonic tendon cells annotated by cell type. Right: the same layout colored by developmental stage (6.5–9.3 post-conception weeks), illustrating temporal dynamics of lineage emergence.

(legend continued on next page)

toward MKX Progenitors, consistent with their early tenogenic identity characterized by high *SCX* but lower *MKX* and *TNMD* expression levels.

To further resolve these differentiation pathways, Palantir pseudotime analysis was applied. Using extreme values of multiscale diffusion components, MSC Precursors were defined as the initiation state, while Chondrocytes (chondrogenic lineage), COL6A6 Progenitors (LCT lineage), and MKX Progenitors (tenocyte lineage) served as terminal states (Figure S4A). Consistent with RNA velocity, Palantir-CellRank analysis mapped a trajectory from MSC Precursors to MKX Progenitors via an intermediate SCX Progenitor stage, recapitulating known tenocyte differentiation hierarchies (Figures 4D and S4B–S4D). Chondrogenic differentiation proceeded from RUNX2 Progenitors to SOX5 Progenitors, ultimately forming embryonic Chondrocytes. Fate probability visualization confirmed these transitions across donor ages while also indicating that most progenitor populations retained some degree of plasticity at later stages (Figures 4D and 4E).

Analysis of lineage-driving genes revealed strong anti-correlation patterns between competing lineages (Figure 4F). In the tenocyte lineage, genes such as *SCX*, *MKX*, *TNMD*, *ABI3BP*, and *GAS2* were negatively correlated with LCT- and chondrocyte-associated markers, suggesting roles in reinforcing tenogenic identity. Conversely, *TWIST2*, *DCLK1*, and *CELF2* were anti-correlated with tenogenic markers, supporting maintenance of LCT fate. The anti-correlation between LCT and chondrogenic lineages was less pronounced, indicating closer transcriptional relationships between these cell types. Genes like *LUM* and *GPC3* selectively anti-correlated with tenocyte but not chondrocyte differentiation, highlighting lineage-specific regulatory mechanisms.

Integration of embryonic and fetal datasets supported these inferred trajectories (Figure 5A). MSC Precursors first differentiated into SCX and COL6A6 Progenitors (6.5–8.4 pcw), followed by RUNX2 and SOX5 Progenitors (8.4–9.0 pcw). By 9.3–12 pcw, SCX Progenitors gave rise to FGF14 THBS4 and ABI3BP GAS2 Fibroblasts, while MKX Progenitors contributed specifically to ABI3BP GAS2 Fibroblasts (Figure 5B). In parallel, COL6A6 Progenitors differentiated into COL3A1 PI16, COL6A6 FNDC1, and NEGR1 SCN7A Fibroblasts, reflecting their broader differentiation potential.

Finally, SCENIC regulatory network analysis of 20 pcw and earlier fetal tendon populations revealed an absence of definitive fibroblast-specific regulons (Figure S5), indicating that tendon fibroblast transcriptional identities remained heterogeneous and incompletely matured at this developmental stage.

Developing tendons exhibit histological features typically associated with adult tendon pathology

To initiate comparisons between developing and aged tissues, histological and histochemical analyses were performed on fetal and adult Achilles and quadriceps tendons. Fetal tendons exhibited distinct structural characteristics compared to adult tendons, including high cellularity, increased vascularity, and abundant ground substance rich in acidic polysaccharides—features typically associated with tendon pathology in adulthood.[94–96]

Although fetal collagen fibers appeared well organized, their arrangement was less uniform than in mature tendons. Picrosirius red staining under polarized light revealed progressive collagen maturation: early fetal tendons (11–12 pcw) displayed loosely arranged, thin fibers with low birefringence (consistent with type III collagen), while 16–20 pcw tendons showed thicker fibers with a transition from blue/green to red birefringence, indicative of type I collagen (Figure S6). Collagen crimp patterns also evolved over time, becoming more elongated and approaching the adult tendon architecture by 20 pcw.

Quantitative image analysis confirmed a progressive decline in nuclear density from 11 pcw through adulthood (Figure S7). Nuclear areas decreased between 11 and 15 pcw before returning to 11 pcw baseline levels by 20 pcw. In adult samples, nuclear size was variable, with some nuclei smaller and others comparable to those in late-stage fetal tendons. Nuclear morphology transitioned from elongated to rounded and back to elongated during fetal development, whereas adult tendons exhibited regional variability in nuclear shape associated more with microanatomical site differences than chronological age (Figure S8). Given limited donors per time point and regional variability, we cannot exclude donor or microanatomical sampling effects. We therefore interpret this as putative transient changes consistent with bursts in fetal matrix remodeling while acknowledging alternative explanations (e.g., shifts in cell-cycle composition or paracrine signaling). Prospective validation in larger, well-controlled cohorts will be important to confirm this pattern.

Adult tendon fibroblasts diverge transcriptionally from developmental lineages, unlike conserved non-fibroblast populations

Integration of embryonic (6–9 pcw), fetal (12–20 pcw), and adult (25–76 years) Achilles and quadriceps tendon datasets using scANVI showed that developing and adult fibroblast populations remained largely distinct (Figures 6A and S9). However, fibroblasts from ruptured adult quadriceps tendons (referred here as COL3A1hi Fibroblasts and named ADAM12hi Fibroblasts in a recent publication by Mimpen et al.[30]) showed higher expression of top differentially expressed genes (DEGs) from fetal

(B) Dot plots of scaled, log1p-normalized gene expression showing differentially expressed genes across embryonic tendon cell types. Lineages are grouped by putative fate: fascicular tendon, loose connective tissue (LCT), or chondrocyte. Dot size reflects cell type abundance.

(C) RNA velocity analysis overlaid on a force-directed layout derived from t-SNE embeddings calculated using multiscale diffusion components. Arrows indicate the predicted future transcriptional state and directionality of gene expression changes among progenitor populations.

(D) CellRank-directed partition-based graph abstraction map showing lineage trajectories from MSC Precursors toward three terminal fates: fibrillar tendon (red), loose connective tissue fibroblast (blue), and chondrocyte (orange). Arrow thickness reflects transition probability; dashed lines indicate weaker transitions. Pie charts show fate likelihoods per cell type, and bar plots in (E) quantify absorption probabilities.

(F) Correlation plot of CellRank-derived lineage driver genes. Each axis represents gene correlation with terminal cell fates. Top 15 drivers are annotated for each lineage.

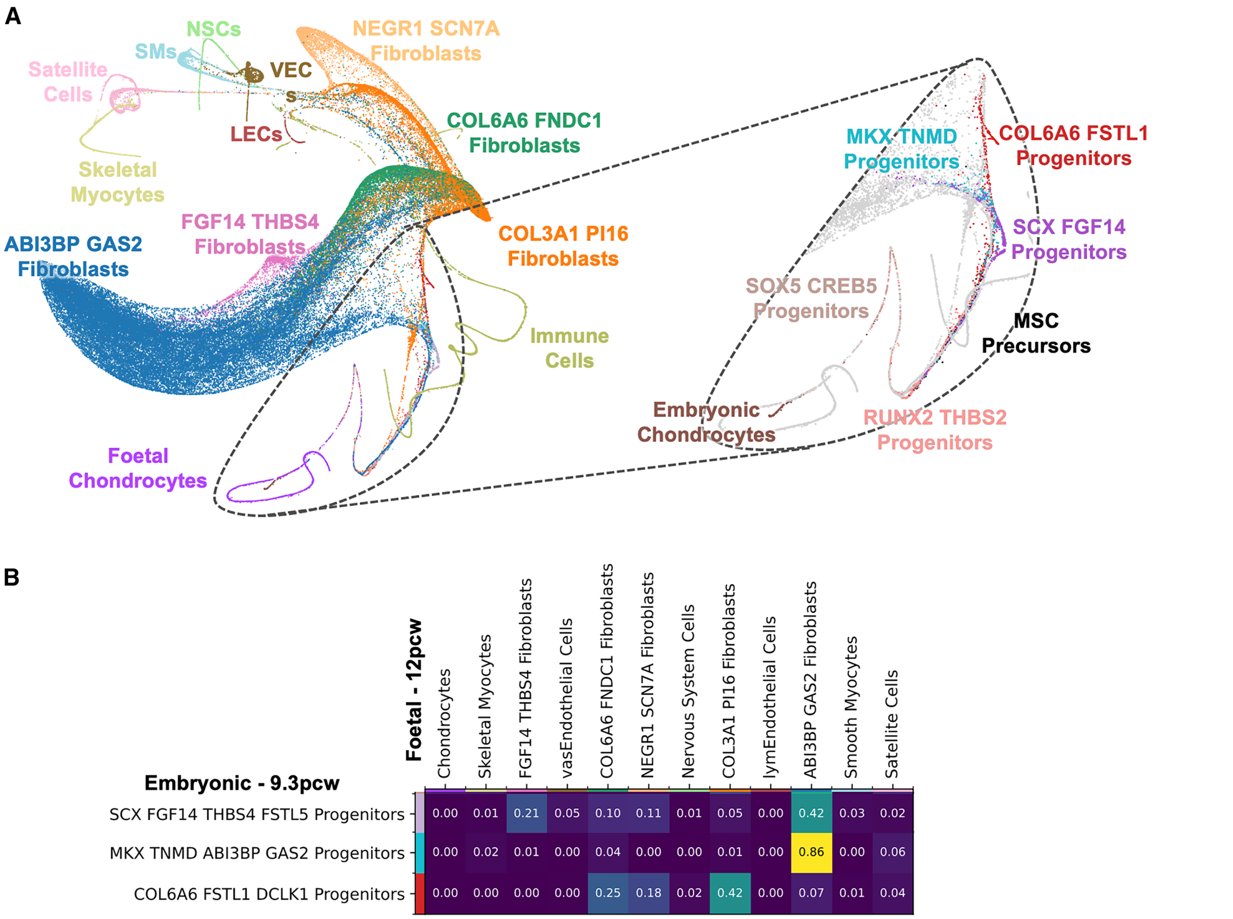

Figure 5. Integration of embryonic and fetal datasets reveals continuity and divergence in fibroblast lineage trajectories

(A) Force-directed layout of scGen-integrated embryonic (6–9 pcw) single-cell RNA-seq and fetal (12–20 pcw) single-nucleus RNA-seq datasets. Inset highlights embryonic tendon cells, which remain largely segregated, reflecting transcriptional divergence likely due to developmental stage differences and platform-specific biases.

(B) Transition matrix from moscot analysis showing the inferred descendants of 9.2 pcw fibroblast-lineage progenitor populations within 12 pcw fetal samples. Values represent the proportion of cells predicted to transition into each differentiated fibroblast subtype.

ABI3BP GAS2 and COL6A6 FNDC1 Fibroblasts compared to healthy adult tendons, suggesting a shift toward a developmental-like state potentially linked to repair processes (Figures 6B and S10). In contrast, fetal DEGs overlapped significantly with non-fibroblast adult populations, including immune, endothelial, muscle, and nervous system-associated cells (Figures 6A and 6B).

Cell-type harmonization using CellHint refined annotations across datasets, confirming cross-stage consistency and revealing developmental relationships between fetal and adult tendon populations (Figure S11). For instance, fetal immune cells aligned to adult *MERTK*-expressing Macrophages, and fetal Skeletal Myocytes mapped to Transitional and Fast-twitch Skeletal Muscle Cells in adult Achilles tendons. Adult tendons also exhibited increased cellular diversity, including Adipocytes and NR4A1hi Fibroblasts. While fetal and adult Achilles tendon nervous system-associated cells showed continuity, adult quadriceps tendon nervous system-associated cells appeared developmentally distinct, lacking a clear fetal counterpart.

This analysis also supported previous trajectory findings. Embryonic MKX Progenitors were linked to fetal ABI3BP GAS2 Fibroblasts; SCX Progenitors to fetal FGF14 THBS4 Fibroblasts; COL6A6 Progenitors to fetal COL3A1 PI16 Fibroblasts; and SOX5 Progenitors, RUNX2 Progenitors, and embryonic Chondrocytes to fetal Chondrocytes (Figure S11). While fetal ABI3BP GAS2 Fibroblasts were traced to adult ITGA10hi and FBLN1hi Fibroblasts in healthy Achilles and quadriceps tendons, respectively, fetal COL3A1 PI16 and NEGR1 SCN7A Fibroblasts were linked to NEGR1hi and ABCA10hi Fibroblasts in adult tendons. Notably, embryonic RUNX2 Progenitors, fetal Chondrocytes, and injury-responsive TSPC marker-expressing COL6A6 FNDC1 Fibroblasts were traced to ruptured quadriceps COL3A1hi Fibroblasts. In contrast, MTJ-associated fetal FGF14 THBS4 Fibroblasts had no clear adult counterpart, indicating a likely transient developmental role.

DGE analysis between fetal and adult quadriceps tendon fibroblasts revealed substantial transcriptional shifts. In adults, 262 pathways were downregulated (Figure 7A), encompassing

CellPress
OPEN ACCESS

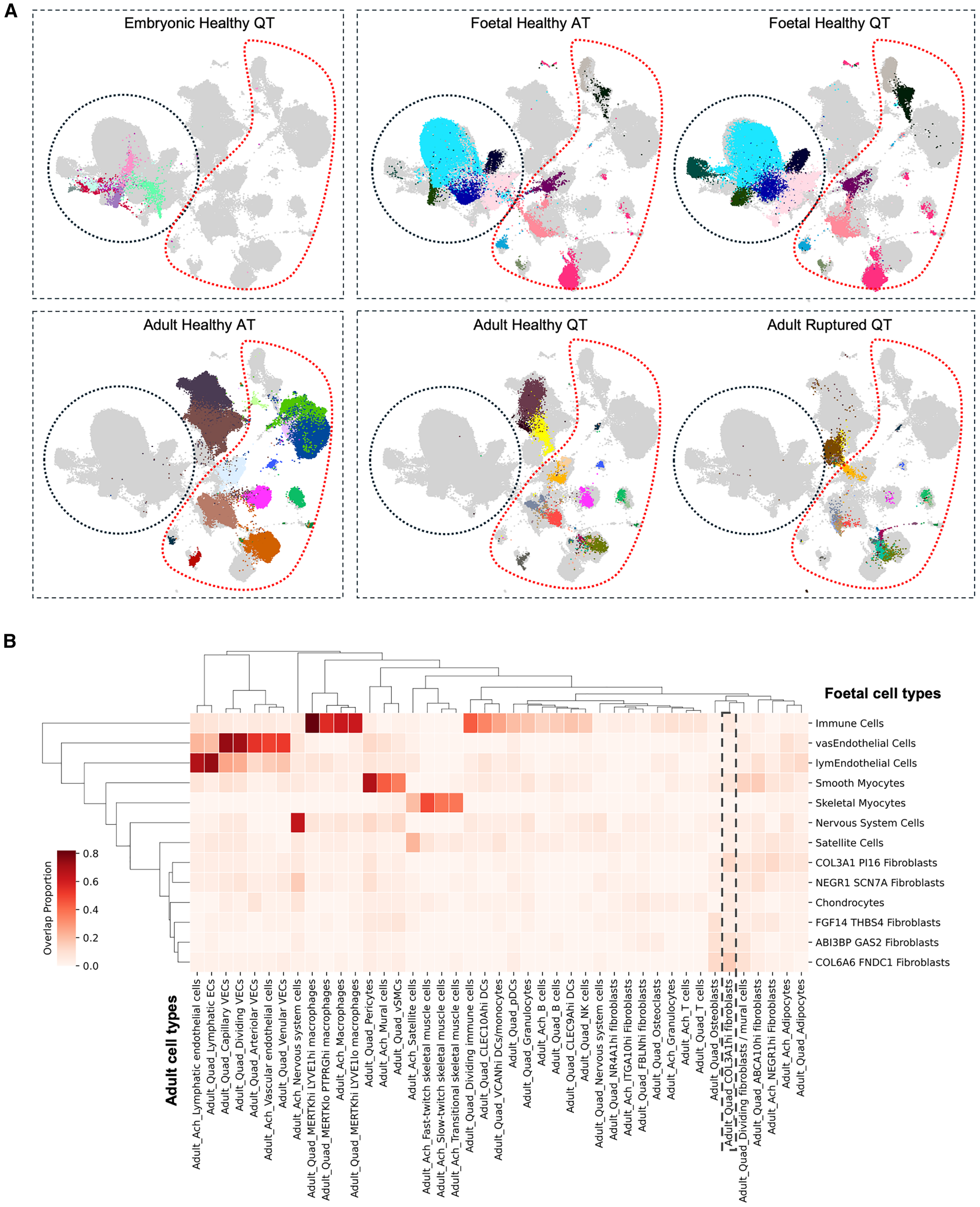

Figure 6. Cross-stage and cross-dataset harmonization reveals conserved and divergent tendon cell identities
(A) ScANVI-integrated embryonic, fetal, and adult tendon datasets. All embryonic and fetal fibroblast populations are outlined in blue. All non-fibroblast populations are outlined in red. The populations that are not outlined are exclusively adult fibroblasts. Annotated adult datasets were provided by Dr Carla Cohen (Achilles tendon) and Dr Jolet Mimpen (quadriceps tendon); annotations can be found, in full, in Figure S9.

(legend continued on next page)

energy metabolism, nucleotide and protein biosynthesis, apoptosis, cell-cycle regulation, telomere maintenance, and stress response processes (Table S3). In contrast, 35 pathways were upregulated (Figure 7A; Table S3), including cellular signaling, structural organization, cell migration, and nervous system development. Adult fibroblasts significantly upregulated *ECM2* while downregulating genes associated with cartilage (*COL2A1*, *COL9A1*, and *COL9A3*), early fibrillogenesis (*LUM*), growth factor signaling (*IGFBP2*, *IGFBP4*, and *IGFBP5*), elastic fiber formation (*EFEMP1*, *EFEMP2*, *EMILIN1*, *FBLN1*, *MFAP2*, and *MFAP4*), cell adhesion (T*GFBI* and *VWA1*), collagen deposition (*CTHRC1* and *PCOLCE*), and calcification inhibition (*MGP*; Figures S12, S13, S14, and S15). These findings suggest a transition from the highly metabolic and biosynthetically active fetal state to a more maintenance-oriented adult fibroblast phenotype.

Finally, SCENIC analysis of aged human tendons revealed strong cell type-specific regulons, with limited regulon conservation across development and adulthood. Notably, the RARG regulon remained active in embryonic RUNX2 Progenitors, fetal Chondrocytes, and adult ruptured tendon COL3A1hi Fibroblasts (Figure 7B), regulating 19 genes (e.g., *ARHGEF10L*, *PIK3R1*, *BOC*, *PCDH9*, *ZFHX3*, *PDGFD*, and *DCLK1*) linked to epithelial-to-mesenchymal transition (EMT) and wound healing.[97–100] The EMT regulators controlled by SCX—*TWIST1* and *SNAI1*[101]—were highly activated in ruptured COL3A1hi Fibroblasts compared to healthy fibroblasts. While the TWIST1 regulon was associated with genes regulating ECM remodeling and fibroblast function (e.g., *BCL7A*, *BICC1*, *CACNB2*, *COL1A2*, *HMCN1*, *HTRA1*, and *PLEKHA5*), the SNAI1 regulon was linked to genes involved in angiogenesis, cytoskeletal organization, and fibrosis (e.g., *COL4A1*, *EFNB2*, *KDR*, *MIR31HG*, *PALLD*, and *SLC45A4*). Additionally, both ruptured and healthy adult fibroblasts exhibited high activation of the TBX15 regulon, which encompassed 615 associated genes linked to growth, differentiation, ECM organization, and tissue-specific developmental pathways, including Wnt and Notch signaling.

DISCUSSION

Tendon development and homeostasis rely on complex transcriptional programs that guide progenitor differentiation, ECM organization, and tissue specialization. By integrating single-cell, single-nucleus, and spatial transcriptomic datasets spanning embryonic, fetal, and adult human tendons, we mapped the emergence and diversification of fibroblast populations across developmental stages and following tendon injury. Our findings reveal substantial transcriptional and functional reprogramming of tendon fibroblasts from early development to adulthood, with the preservation of certain regulatory features—particularly in the context of repair.

Differentiation trajectories and lineage commitments in embryonic and fetal tendon development

We show that during early development (6.5–8.4 pcw), multipotent MSC Precursors are predicted to diverge into distinct tendon, LCT, and chondrocyte lineages. *SCX*-expressing tendon progenitors appear to transition into MKX Progenitors, committing to fibrillar ECM production and forming the core tendon fibroblast lineage. In parallel, MSC-like COL6A6 Progenitors likely give rise to LCT fibroblasts, while RUNX2 and SOX5 Progenitors mark early chondrogenic differentiation, progressing toward mature embryonic Chondrocytes through coordinated activity of *SOX5*, *SOX6*, *SOX9*, and *RUNX3*.

By the second trimester (12–20 pcw), tendon fibroblasts diversify into spatially and transcriptionally distinct subpopulations. Within the fibrillar core, ABI3BP GAS2 Fibroblasts and more differentiated KERA TNMD Fibroblasts contribute to matrix organization, remodeling, and tenocyte differentiation, with the latter population becoming more prevalent over time. A small CHSY3 MECOM Fibroblast population may contribute to ECM hydration, mineralization, and tendon-bone interface remodeling. Within the LCT, CREB5 PRG4 Fibroblasts likely facilitate lubrication and ECM maintenance under mechanical load, COL3A1 PI16 Fibroblasts support tissue remodeling and matrix adaptation through cell-ECM interactions, and COL6A6 FNDC1 Fibroblasts show early injury-responsive and fibrillogenesis-associated features, suggesting a developmental link to adult repair processes. At the tendon-muscle junction, FGF14 THBS4 Fibroblasts contribute to ECM synthesis, vascularization, and immune interactions but decline in abundance by 20 pcw, indicating a transient role in MTJ development. Similarly, NEGR1 SCN7A Fibroblasts—localized to the muscle endo- and perimysium—appear to support tendon-muscle integration but diminish with maturation.

A key insight from this study is the identification of a previously uncharacterized *SCX*-negative progenitor population expressing *COL6A6* and *FSTL1*, which branches from MSC Precursors into both LCT and muscle-associated fibroblasts. These progenitors are defined by regulatory programs involving *TWIST2*, *OSR1*, *OSR2*, *RUNX1T1*, *DCLK1*, and *CELF2* and exhibit strong anti-correlation with the SCX-MKX-TNMD-ABI3BP-GAS2 tenogenic program. Their fetal descendants express injury-responsive TSPC markers *TPPP3* and *PDGFRA*, implicating this axis as a potential contributor to both tendon development and regeneration.[13,38]

While our data confirm the presence of *SCX*-expressing progenitors from early embryogenesis through the second trimester, further work is needed to clarify the relationship between the *SCX*-negative COL6A6 lineage at 8.4 pcw and earlier *SCX*-expressing FGF14 Progenitors identified at 7.2 pcw. Existing studies have primarily focused on *SCX*-expressing lineages, with limited exploration of LCT origins. This gap likely reflects both a historical lack of definitive LCT markers and technical challenges in imaging early-stage tendons,

(B) Clustermap shows normalized overlap of the top 50 differentially expressed genes in fetal cell types (rows) with those identified in adult cell types (columns), computed using the Wilcoxon rank-sum test. Overlap is expressed as a proportion of the fetal marker gene set, with darker shades indicating higher marker overlap. Adult quadriceps COL3A1hi population (outlined in black) is the unique fibroblast population specific to ruptured adult tendon.
Ach/AT: Achilles tendon; Quad/QT: quadriceps tendon.

CellPress
OPEN ACCESS

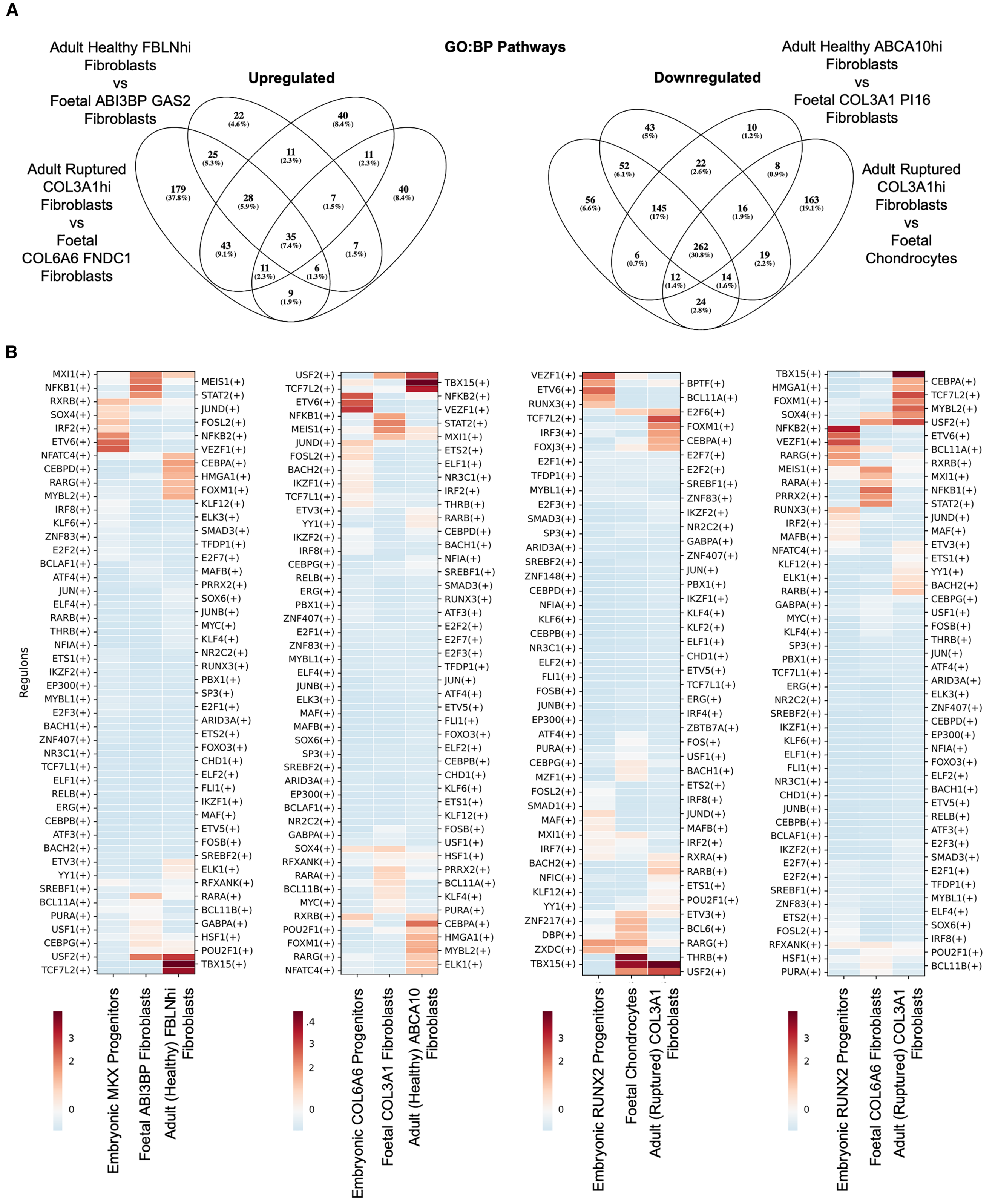

(legend on next page)

where *SCX*-negative domains may be difficult to resolve. For example, murine studies using Scx-GFP reporters suggest uniform Scx expression across tendons until late fetal development,[102,103] potentially overlooking early LCT heterogeneity.

Fibroblast plasticity contrasts with conserved non-fibroblast programs across tendon development and aging

Beyond fibroblasts, several non-fibroblast populations play key roles in tendon development and homeostasis. *PECAM1*+ vascular endothelial cells, *PROX1*+ lymphatic endothelial cells, and *ACTA2*+ smooth myocytes are present during development. Cells expressing neural markers such as *NRXN1*, *NCAM2*, and *SOX10* suggest a role in neural development and tendon innervation. Furthermore, *PAX7*-expressing satellite cells and myocytes demonstrate gene expression patterns linked to muscle differentiation and function, underscoring the interconnectedness of tendon and muscle development. Immune cells, predominantly macrophages expressing *F13A1*, *MRC1*, and *CD163*, are also abundant, consistent with their known roles in tissue patterning and immune regulation.[43,104–107]

Notably, these populations persist into adult tendons, maintaining conserved transcriptional programs across developmental stages. This suggests they serve fundamental, stable roles in tendon physiology, extending beyond development to support tissue maintenance, innervation, and vascularization across the lifespan. In contrast, fibroblasts exhibit significant age- and health-specific transcriptional divergence. Dimensional reduction embeddings show clear separation between developmental and adult fibroblast populations, reflecting adaptive transcriptional programs tuned to tissue state and biomechanical demands. While certain populations—such as ABI3BP GAS2 Fibroblasts—persist across time and align with adult ITGA10hi and FBLN1hi subsets, others, like FGF14 THBS4 Fibroblasts at the MTJ, appear transient and lack clear adult counterparts. Furthermore, some cell populations, including adipocytes and neural cell types, are exclusive to adult tendons.

A particularly striking observation is the link between adult rupture-specific COL3A1hi Fibroblasts and embryonic RUNX2 Progenitors, fetal LCT COL6A6 FNDC1 Fibroblasts, and Chondrocytes. These adult fibroblasts express genes typically active during fetal development, suggesting a partial reactivation of fetal programs following injury—a mechanism potentially beneficial for initiating repair.[108–110] However, these reactivated cells lack TSPC markers and do not recapitulate full regenerative programs observed in fetal tendons.[6,8] Their precise origin—whether reprogrammed fascicular or LCT fibroblasts—remains unclear.

As tendons age, the synthetic and metabolic activity of fibroblasts declines, correlating with increased matrix stiffness and reduced elasticity.[111–116] Our DGE analysis supports this shift, revealing a global transition from biosynthetic and proliferative programs to maintenance-focused gene expression in adult fibroblasts. Upregulated pathways include cell signaling, structural regulation, and nervous system development, while genes involved in cartilage and elastic fiber formation are downregulated. Notably, adult fibroblasts also exhibit reduced expression of proteoglycans and early fibrillogenic collagens, aligning with diminished ECM turnover after tendon maturation.[117–120]

Regulatory network analysis further underscores this divergence. Few regulons are shared between developmental and adult tendon fibroblasts, highlighting transcriptional reprogramming across maturation. For example, adult rupture-specific fibroblasts activate EMT-related regulons associated with migratory and invasive phenotypes commonly seen in wound healing and fibrosis,[121] suggesting that this process may be involved in adult tendon injury repair.

These findings diverge from murine studies of tendon repair. Our human quadriceps tendon samples were collected 8–9 days post-rupture, corresponding to the fibroblastic proliferative phase characterized by collagen III-rich granulation tissue, prior to collagen I-mediated remodeling.[14,122–124] In contrast, mouse models at this stage show active infiltration of migrating LCT cells expressing *Acta2*, *Sca1*, *Axin2*, or *Glast*.[9,12,39,40,42,45,46] We did not observe expression of these markers in rupture-specific COL3A1hi or other adult fibroblasts in our dataset. A likely factor is the advanced age of the human donors (67–75 years), in contrast to young adult mice (2.5–6 months) used in tendon injury experimental models.[9,12,39,40,42,45,46] To bridge this gap, future work should directly compare human tendon populations across developmental and post-injury stages with murine and zebrafish models, enabling more accurate mapping of tendon repair processes across ages and species.

Finally, research to date suggests that tendon healing is most effective before significant mechanical loading and fibril expansion occur, with regenerated tendons typically displaying smaller, more immature collagen fibrils compared to uninjured tendons.[5,9] In this study, ruptured adult COL3A1hi Fibroblasts downregulated key genes involved in elastic fiber production (*EMILIN1*, *FBLN1*, and *MFAP2*) and collagen fibril initiation, deposition, and processing (*COL11A1*, *CTHRC1*, *PCOLCE*, and *MGP*), potentially impairing structural repair. Alongside reduced metabolic and biosynthetic activity, these observations align with previous findings that fetal tendon fibroblasts are more proliferative and metabolically active[20,21] and that ECM turnover in adult tendons is minimal after late adolescence.[119,120] Together, our findings suggest that adult tendon fibroblasts

Figure 7. Conserved and divergent pathways and gene regulatory programs in fibroblasts across tendon development and aging

(A) Venn diagrams of intersecting upregulated or downregulated gene ontology biological process (GO:BP) pathways in CellHint-paired fetal and adult cell types. Only significant (<0.01 BH-FDR) pathways with term sizes of 20–500 were used.

(B) Hierarchical clustermaps showing *Z* score normalized SCENIC regulon activity in selected fibroblast subtypes from embryonic, fetal, and adult tendon datasets. Regulons are clustered using average linkage and Euclidean distance; cell types remain in original order. Color scale indicates relative regulon activity (blue: below mean, white: mean, red: above mean). Only regulons shared across all datasets are shown. Regulon names are alternately labeled on the left and right axes for clarity.

CellPress
OPEN ACCESS

primarily maintain tissue homeostasis rather than help regenerate it, a limitation that may underlie the poor repair outcomes observed clinically.

In summary, by integrating snRNA- and spatial RNA-sequencing approaches, we mapped the cellular and transcriptional architecture of developing and adult human tendons. We defined distinct cell types within fetal Achilles and quadriceps tendons and reconstructed the developmental trajectories of seven embryonic progenitors contributing to the formation of fibrillar core fibroblasts, loose connective tissue fibroblasts, and chondrocytes. Comparative analyses between embryonic, fetal, and aged adult tendons revealed profound shifts in cell composition, density, and ECM organization: while vascular, immune, neural, and muscle-associated non-fibroblast populations remained transcriptionally conserved across the lifespan, fibroblasts showed striking age- and health-specific divergence, transitioning from metabolically active, matrix-synthesizing phenotypes in development to homeostatic maintenance states in maturity. Notably, rupture-associated adult fibroblasts partially reactivated developmental gene programs but failed to express canonical TSPC markers or fully recapitulate regenerative trajectories, highlighting limitations in the adult tendon's capacity for repair.

These findings provide a comprehensive atlas of tendon cell diversity across the human lifespan. They underscore the need to characterize fibroblast plasticity and immune-stromal interactions in greater detail and point to specific cellular targets and regulatory pathways that may be leveraged to enhance repair. Future work translating these insights into therapeutic strategies—particularly those that emulate or reintroduce regenerative developmental programs—could help address the persistent clinical challenges of tendon injury, degeneration, and aging.

Limitations of the study

This work is primarily descriptive and relies on multi-omic and computational analyses (snRNA-seq, spatial transcriptomics, RNA velocity, pseudotime inference, and regulon analysis). No perturbation, lineage-tracing, mechanobiology, or protein-level validation assays were performed; all mechanistic statements are therefore hypothesis-generating, and causal roles of specific regulators or fibroblast states remain to be established. In particular, orthogonal validation and colocalization of marker expression (e.g., by multiplexed immunostaining or RNA *in situ* hybridization) are not provided, so cell-type annotations and spatial assignments remain putative, and precise cell identities cannot be definitively established.

This study has several important limitations. First, the study cohort and tissue sampling impose several constraints. The adult injury cohort represents a narrow clinical context, comprising primarily acute complete quadriceps tendon ruptures sampled approximately 1 week post-injury. Consequently, the transcriptional states we describe may not generalize to chronic tendinopathies, partial tears, other tendon types, or alternative healing environments. Although our adult dataset spans 25–76 years, current sample sizes do not support robust discrimination between younger homeostatic and older aging adult phases; larger cohorts will be required for more granular life-stage analyses. Tissue availability limited histological validation, preventing fully balanced groups of healthy and diseased tendons encompassing all microanatomical regions and resulting in substantial inter- and intra-donor variability. In addition, the absence of pediatric samples and the limited extent of imaging-based validation constrain our ability to directly connect developmental and adult fibroblast phenotypes and to generalize their likely functions.

Second, the spatial transcriptomic data are subject to important technical limitations. Visium spatial RNA-sequencing is intrinsically restricted by spot resolution, capturing signal from a mean of ∼17 cells per spot in our samples. This precludes precise delineation of microscale cellular niches, signaling gradients, and local cell-cell or cell-matrix interactions within developing tendon, and limits our ability to assign transcriptional states to exact anatomical structures. Unsupervised decomposition of deconvolved abundance maps using non-negative matrix factorization provides partial validation by revealing independent spatial modules corresponding to specific fibroblast states, but higher-resolution spatial multi-omics will be required to more accurately resolve tendon microenvironments.

Third, our computational framework is affected by several methodological and integration challenges stemming from complex sample metadata, multiple experimental batches, and distinct sample processing and sequencing protocols. Because embryonic tendons are extremely small, we inferred tendon signatures at 8 pcw from a single spatial RNA-seq section rather than from dedicated single-cell or single-nucleus profiles. This transfer of information from lower- to higher-resolution data may have led to omission or mis-assignment of rare cell types and could contribute to the apparent absence of canonical undifferentiated mesenchymal progenitors or typical $SOX9^{+}SCX^{+}$ attachment-unit cells.[125,126] Cross-modality integration of embryonic scRNA-seq with fetal/adult snRNA-seq introduces additional, known biases. snRNA-seq enriches for nuclear/pre-mRNA (higher intronic content) and often yields lower detection of cytoplasmic and secreted transcripts (e.g., some cytokines, senescence-associated secretory phenotype (SASP) transcripts), whereas scRNA-seq captures mature cytoplasmic mRNA and may detect stress-response and secretory programs more readily. These modality differences likely contribute, alongside true developmental divergence, to the residual separation of embryonic versus fetal/adult fibroblasts in integrated embeddings (Figure 5A). Accordingly, our differential analyses compare within modality where possible, and cross-modality contrasts are interpreted with caution.

Finally, there are important limitations in the interpretation of fibroblast states and their roles. Our delineation of discrete fibroblast populations and their proposed functions in tendon homeostasis, loading adaptation, and repair is based primarily on transcriptional profiles. These putative states and their functions require direct experimental confirmation. High-resolution RNA *in situ* hybridization (e.g., RNAscope, merFISH) and multiplexed immunofluorescence could be used to validate the existence, spatial organization, and marker expression of the identified fibroblast subsets *in situ*. In parallel, functional studies followed by assays of stemness, responsiveness to biological cues, wound closure, collagen-gel contraction, and biomechanical properties will be needed to test their contributions to matrix

remodeling and tissue repair. Gene knockdown or overexpression studies targeting priority regulators, with subsequent evaluation in appropriate *in vivo* models of tendon injury or disease, will be critical to establish causality and to identify actionable therapeutic targets.

RESOURCE AVAILABILITY

Lead contact

Further information and requests for resources and reagents should be directed to and will be fulfilled by the lead contact, Sarah Snelling (sarah.snelling@ndorms.ox.ac.uk).

Materials availability

This study did not generate new unique reagents.

Data and code availability

- Processed and annotated fetal tendon single-nucleus RNA-sequencing data are available through CellxGene: https://cellxgene.cziscience.com/collections/7b9ae565-a781-433d-98d4-430394e7802a.
- Raw fetal tendon single-nucleus RNA-seq data have been deposited at the Gene Expression Omnibus (GEO: GSE317327) and are publicly available.
- Visium fetal spatial data have been deposited at Zenodo: http://www.doi.org/10.5281/zenodo.18403632 and are publicly available.
- All codes used for the analysis are available at https://github.com/AlinaKurjan/DPhilCode, deposited at Zenodo: http://www.doi.org/10.5281/zenodo.18379150.
- Any additional information required to reanalyze the data reported in this paper is available from the lead contact upon request.

ACKNOWLEDGMENTS

We are grateful to the staff of the Human Developmental Biology Resource (UCL Institute of Child Health, London) for their efforts in obtaining the human fetal material, as well as the donors who consented to the use of the material. In addition, we thank the staff at the Histology Service (Kennedy Institute of Rheumatology, Oxford) for their help with histology and histochemistry. We also thank Dr Peng He, Dr John E. G. Lawrence, and Prof Sarah A. Teichmann for processing the embryonic single-cell and spatial RNA-sequencing data of whole embryonic limbs to meet the requirements of this work and for aid with analysis and interpretation of results; Dr Claudia Paul for providing stained sections of adult supraspinatus tendons for comparisons; Chinemerem T. Ikwuanusi for assistance with tissue processing and immunofluorescence staining; and Naomi Gray for assistance with logistics. This work was supported by funding from the Balliol College Snell Scholarship together with the Oxford-Medical Research Council Doctoral Training Partnership (https://www.medsci.ox.ac.uk/study/graduateschool/mrcdtp/) (A.K.); the National Institute for Health Research (NIHR; https://www.nihr.ac.uk/); the NIHR Oxford Biomedical Research Centre (BRC; https://oxfordhealthbrc.nihr.ac.uk/) (NIHR203311; M.J.B. and S.J.B.S.); and the Chan Zuckerberg Initiative (https://chanzuckerberg.com/) (2019–002426; J.Y.M., L.R.-M., M.J.B., A.P.C., and S.J.B.S.). The views expressed are those of the authors and not necessarily those of the NHS, the NIHR, or the Department of Health. The funders had no role in study design, data collection and analysis, decision to publish, or preparation of the manuscript.

AUTHOR CONTRIBUTIONS

S.J.B.S., M.J.B., C.D.B., A.P.C., and A.K. conceived and designed the study. A.K., M.J.B., J.Y.M., L.R.-M., and A.C.A. collected samples. A.K., J.Y.M., L.R.-M., and A.C.A carried out the experiments. A.K. performed the bioinformatics analyses and analyzed and interpreted data. J.Y.M. provided processed and annotated data for adult quadriceps tendons. C.J.C. provided processed and annotated data for adult Achilles tendons. M.N. carried out quantitative image analysis. A.K., S.J.B.S., and M.J.B. wrote the manuscript. All authors provided critical feedback and approved the final version of the manuscript.

DECLARATION OF INTERESTS

A.K. is a full-time employee of Caeruleus Genomics Ltd (Entelo Bio). A.P.C. is a co-founder of Caeruleus Genomics Ltd (Entelo Bio) and is an inventor on several patents related to sequencing technologies filed by Oxford University Innovations.

STAR★METHODS

Detailed methods are provided in the online version of this paper and include the following:

- KEY RESOURCES TABLE
- EXPERIMENTAL MODEL AND STUDY PARTICIPANT DETAILS
  - Human fetal tissue samples
  - Human adult tissue samples
- METHOD DETAILS
  - Fetal and adult tendon tissue processing
  - Fetal and adult tendon nuclei isolation
  - Fetal and adult tendon single-nucleus RNA-sequencing
  - Adult tendon snRNA-seq data processing
  - Fetal tendon snRNA-seq data processing
  - 10X visium spatial RNA-sequencing of fetal samples
  - Fetal spatial RNA-seq data processing
  - Fetal cell type mapping to spatial coordinates
  - Immunofluorescence staining and imaging of fetal tendons
  - Histochemistry of fetal and adult tendon tissues
  - Embryonic whole limb data processing
  - Embryonic tendon RNA velocity analysis
  - Embryonic and fetal tendon trajectory and fate analysis
  - Embryonic and fetal data integration and trajectory inference
  - Embryonic, fetal and adult data integration
  - Gene regulatory network analysis
- QUANTIFICATION AND STATISTICAL ANALYSIS
  - Quantification of fetal cell type compositional changes
  - Quantifying metrics from tendon histology
  - Fetal tendon pseudobulk differential gene expression analysis
  - Fetal and adult tendon pseudobulk differential gene expression analysis

SUPPLEMENTAL INFORMATION

Supplemental information can be found online at https://doi.org/10.1016/j.celrep.2023.113061.

Received: June 20, 2025
Revised: December 2, 2025
Accepted: February 12, 2026
Published: March 20, 2026

REFERENCES

1. Magnusson, S.P., Langberg, H., and Kjaer, M. (2010). The pathogenesis of tendinopathy: balancing the response to loading. Nat. Rev. Rheumatol. *6*, 262–268.
2. Malliaras, P., Barton, C.J., Reeves, N.D., and Langberg, H. (2013). Achilles and Patellar Tendinopathy Loading Programmes. Sports Med. *43*, 267–286.
3. Simpson, M., Rio, E., and Cook, J. (2016). At What Age Do Children and Adolescents Develop Lower Limb Tendon Pathology or Tendinopathy? A Systematic Review and Meta-analysis. Sports Med. *46*, 545–557.
4. Millar, N.L., Silbernagel, K.G., Thorborg, K., Kirwan, P.D., Galatz, L.M., Abrams, G.D., Murrell, G.A.C., McInnes, I.B., and Rodeo, S.A. (2021).

Tendinopathy. Nat. Rev. Dis. Primers *7*, 1–21. https://doi.org/10.1038/s41572-020-00234-1.

5. Ansorge, H.L., Adams, S., Birk, D.E., and Soslowsky, L.J. (2011). Mechanical, Compositional, and Structural Properties of the Post-natal Mouse Achilles Tendon. Ann. Biomed. Eng. *39*, 1904–1913.
6. Beredjiklian, P.K., Favata, M., Cartmell, J.S., Flanagan, C.L., Crombleholme, T.M., and Soslowsky, L.J. (2003). Regenerative versus reparative healing in tendon: A study of biomechanical and histological properties in fetal sheep. Ann. Biomed. Eng. *31*, 1143–1152.
7. Chan, B.P., Fu, S.C., Qin, L., Rolf, C., and Chan, K.M. (1998). Pyridinoline in relation to ultimate stress of the patellar tendon during healing: An animal study. J. Orthop. Res. *16*, 597–603.
8. Favata, M., Beredjiklian, P.K., Zgonis, M.H., Beason, D.P., Crombleholme, T.M., Jawad, A.F., and Soslowsky, L.J. (2006). Regenerative properties of fetal sheep tendon are not adversely affected by transplantation into an adult environment. J. Orthop. Res. *24*, 2124–2132.
9. Howell, K., Chien, C., Bell, R., Laudier, D., Tufa, S.F., Keene, D.R., Andarawis-Puri, N., and Huang, A.H. (2017). Novel Model of Tendon Regeneration Reveals Distinct Cell Mechanisms Underlying Regenerative and Fibrotic Tendon Healing. Sci. Rep. *7*, 45238.
10. Lui, P.P.Y., Cheuk, Y.C., Lee, Y.W., and Chan, K.M. (2012). Ectopic chondro-ossification and erroneous extracellular matrix deposition in a tendon window injury model. J. Orthop. Res. *30*, 37–46.
11. Garner, W.L., McDonald, J.A., Koo, M., Kuhn, C., and Weeks, P.M. (1989). Identification of the collagen-producing cells in healing flexor tendons. Plast. Reconstr. Surg. *83*, 875–879.
12. Grinstein, M., Tsai, S.L., Montoro, D., Dingwall, H.L., Zou, K., Sade-Feldman, M., Tanaka, M.J., Capellini, T.D., Rajagopal, J., and Galloway, J.L. (2022). A quiescent resident progenitor pool is the central organizer of tendon healing. bioRxiv. https://doi.org/10.1101/2022.02.02.478533.
13. Harvey, T., Flamenco, S., and Fan, C.M. (2019). A Tppp3+Pdgfra+ tendon stem cell population contributes to regeneration and reveals a shared role for PDGF signaling in regeneration and fibrosis. Nat. Cell Biol. *21*, 1490–1503.
14. Pakshir, P., and Hinz, B. (2018). The big five in fibrosis: Macrophages, myofibroblasts, matrix, mechanics, and miscommunication. Matrix Biol. *68–69*, 81–93.
15. Snedeker, J.G., and Foolen, J. (2017). Tendon injury and repair – A perspective on the basic mechanisms of tendon disease and future clinical therapy. Acta Biomater. *63*, 18–36.
16. Voleti, P.B., Buckley, M.R., and Soslowsky, L.J. (2012). Tendon Healing: Repair and Regeneration. Annu. Rev. Biomed. Eng. *14*, 47–71.
17. Walia, B., Li, T.M., Crosio, G., Montero, A.M., and Huang, A.H. (2022). Axin2-lineage cells contribute to neonatal tendon regeneration. Connect. Tissue Res. *63*, 530–543. https://doi.org/10.1080/03008207.2022.2036732.
18. Galloway, M.T., Lalley, A.L., and Shearn, J.T. (2013). The Role of Mechanical Loading in Tendon Development, Maintenance, Injury, and Repair. JBJS *95*, 1620–1628.
19. Peterson, B.E., Rolfe, R.A., Kunselman, A., Murphy, P., and Szczesny, S.E. (2021). Mechanical Stimulation via Muscle Activity Is Necessary for the Maturation of Tendon Multiscale Mechanics During Embryonic Development. Front. Cell Dev. Biol. *9*, 725563.
20. Ansorge, H.L., Hsu, J.E., Edelstein, L., Adams, S., Birk, D.E., and Soslowsky, L.J. (2012). Recapitulation of the Achilles tendon mechanical properties during neonatal development: a study of differential healing during two stages of development in a mouse model. J. Orthop. Res. *30*, 448–456.
21. Grinstein, M., Dingwall, H.L., O'Connor, L.D., Zou, K., Capellini, T.D., and Galloway, J.L. (2019). A distinct transition from cell growth to physiological homeostasis in the tendon. eLife *8*, e48689.
22. Yin, Z., Hu, J.J., Yang, L., Zheng, Z.F., An, C.R., Wu, B.B., Zhang, C., Shen, W.L., Liu, H.H., Chen, J.L., et al. (2016). Single-cell analysis reveals a nestin+ tendon stem/progenitor cell population with strong tenogenic potentiality. Sci. Adv. *2*, 1600874.
23. De Micheli, A.J., Swanson, J.B., Disser, N.P., Martinez, L.M., Walker, N.R., Oliver, D.J., Cosgrove, B.D., and Mendias, C.L. (2020). Single-cell transcriptomic analysis identifies extensive heterogeneity in the cellular composition of mouse Achilles tendons. Am. J. Physiol. Cell Physiol. *319*, C885–C894.
24. Kendal, A.R., Layton, T., Al-Mossawi, H., Appleton, L., Dakin, S., Brown, R., Loizou, C., Rogers, M., Sharp, R., and Carr, A. (2020). Multi-omic single cell analysis resolves novel stromal cell populations in healthy and diseased human tendon. Sci. Rep. *10*, 13939.
25. Akbar, M., MacDonald, L., Crowe, L.A.N., Carlberg, K., Kurowska-Stolarska, M., Ståhl, P.L., Snelling, S.J.B., McInnes, I.B., and Millar, N.L. (2021). Single cell and spatial transcriptomics in human tendon disease indicate dysregulated immune homeostasis. Ann. Rheum. Dis. *80*, 1494–1497.
26. Steffen, D., Mienaltowski, M., and Baar, K. (2023). Spatial gene expression in the adult rat patellar tendon. Matrix Biol. *19–20*, 100138.
27. Ackerman, J.E., Best, K.T., Muscat, S.N., Pritchett, E.M., Nichols, A.E.C., Wu, C.L., and Loiselle, A.E. (2022). Defining the spatial-molecular map of fibrotic tendon healing and the drivers of Scleraxis-lineage cell fate and function. Cell Rep. *41*, 111706.
28. Mimpen, J.Y., Ramos-Mucci, L., Paul, C., Kurjan, A., Hulley, P.A., Ikwuanusi, C.T., Cohen, C.J., Gwilym, S.E., Baldwin, M.J., Cribbs, A.P., et al. (2024). Single nucleus and spatial transcriptomic profiling of healthy human hamstring tendon. FASEB J. *38*, e23629. https://doi.org/10.1096/fj.202300601rrr.
29. Fu, W., Yang, R., and Li, J. (2023). Single-cell and spatial transcriptomics reveal changes in cell heterogeneity during progression of human tendinopathy. BMC Biol. *21*, 132.
30. Mimpen, J.Y., Baldwin, M.J., Paul, C., Ramos-Mucci, L., Kurjan, A., Cohen, C.J., Sharma, S., Florquin, M.S.N.C., Hulley, P.A., McMaster, J., et al. (2025). Exploring cellular changes in ruptured human quadriceps tendons at single-cell resolution. J. Physiol. *603*, 4535–4554. https://doi.org/10.1113/jp287812.
31. McNeilly, C.M., Banes, A.J., Benjamin, M., and Ralphs, J.R. (1996). Tendon cells in vivo form a three dimensional network of cell processes linked by gap junctions. J. Anat. *189*, 593–600.
32. Kannus, P. (2000). Structure of the tendon connective tissue. Scand. J. Med. Sci. Sports *10*, 312–320.
33. Franchi, M., Trirè, A., Quaranta, M., Orsini, E., and Ottani, V. (2007). Collagen Structure of Tendon Relates to Function. Sci. World J. *7*, 404–420.
34. Kalson, N.S., Lu, Y., Taylor, S.H., Starborg, T., Holmes, D.F., and Kadler, K.E. (2015). A structure-based extracellular matrix expansion mechanism of fibrous tissue growth. eLife *4*, e05958.
35. Steffen, D., Avey, A., Mienaltowski, M.J., and Baar, K. (2023). The rat Achilles and patellar tendons have similar increases in mechanical properties but become transcriptionally divergent during postnatal development. J. Physiol. *601*, 3869–3884.
36. Bi, Y., Ehirchiou, D., Kilts, T.M., Inkson, C.A., Embree, M.C., Sonoyama, W., Li, L., Leet, A.I., Seo, B.M., Zhang, L., et al. (2007). Identification of tendon stem/progenitor cells and the role of the extracellular matrix in their niche. Nat. Med. *13*, 1219–1227.
37. Lui, P.P.Y., and Chan, K.M. (2011). Tendon-Derived Stem Cells (TDSCs): From Basic Science to Potential Roles in Tendon Pathology and Tissue Engineering Applications. Stem Cell Rev. Rep. *7*, 883–897.
38. Staverosky, J.A., Pryce, B.A., Watson, S.S., and Schweitzer, R. (2009). Tubulin polymerization-promoting protein family member 3, Tppp3, is a specific marker of the differentiating tendon sheath and synovial joints. Dev. Dyn. *238*, 685–692.
39. Sakabe, T., Sakai, K., Maeda, T., Sunaga, A., Furuta, N., Schweitzer, R., Sasaki, T., and Sakai, T. (2018). Transcription factor scleraxis vitally

contributes to progenitor lineage direction in wound healing of adult tendon in mice. J. Biol. Chem. *293*, 5766–5780.

40. Dyment, N.A., Liu, C.F., Kazemi, N., Aschbacher-Smith, L.E., Kenter, K., Breidenbach, A.P., Shearn, J.T., Wylie, C., Rowe, D.W., and Butler, D.L. (2013). The Paratenon Contributes to Scleraxis-Expressing Cells during Patellar Tendon Healing. PLOS ONE *8*, e59944.

41. Maeda, T., Sakabe, T., Sunaga, A., Sakai, K., Rivera, A.L., Keene, D.R., Sasaki, T., Stavnezer, E., Iannotti, J., Schweitzer, R., et al. (2011). Conversion of Mechanical Force into TGF-β-Mediated Biochemical Signals. Curr. Biol. *21*, 933–941.

42. Nichols, A.E.C., Wagner, N.W., Ketonis, C., and Loiselle, A.E. (2023). Epitenon-derived cells comprise a distinct progenitor population that contributes to both tendon fibrosis and regeneration following acute injury. bioRxiv. https://doi.org/10.1101/2023.01.30.526242.

43. Howell, K.L., Kaji, D.A., Li, T.M., Montero, A., Yeoh, K., Nasser, P., and Huang, A.H. (2021). Macrophage depletion impairs neonatal tendon regeneration. FASEB J. *35*, e21618.

44. Vinestock, R.C., Felsenthal, N., Assaraf, E., Katz, E., Rubin, S., Heinemann-Yerushalmi, L., Krief, S., Dezorella, N., Levin-Zaidman, S., Tsoory, M., et al. (2022). Neonatal Enthesis Healing Involves Noninflammatory Acellular Scar Formation through Extracellular Matrix Secretion by Resident Cells. Am. J. Pathol. *192*, 1122–1135.

45. Moser, H.L., Doe, A.P., Meier, K., Garnier, S., Laudier, D., Akiyama, H., Zumstein, M.A., Galatz, L.M., and Huang, A.H. (2018). Genetic Lineage Tracing of Targeted Cell Populations During Enthesis Healing. J. Orthop. Res. *36*, 3275–3284.

46. Moser, H.L., Abraham, A.C., Howell, K., Laudier, D., Zumstein, M.A., Galatz, L.M., and Huang, A.H. (2021). Cell lineage tracing and functional assessment of supraspinatus tendon healing in an acute repair murine model. J. Orthop. Res. *39*, 1789–1799.

47. Chen, C.H., Cao, Y., Wu, Y.F., Bais, A.J., Gao, J.S., and Tang, J.B. (2008). Tendon Healing In Vivo: Gene Expression and Production of Multiple Growth Factors in Early Tendon Healing Period. J. Hand Surg. *33*, 1834–1842.

48. Kaji, D.A., Howell, K.L., Balic, Z., Hubmacher, D., and Huang, A.H. (2020). Tgfβ signaling is required for tenocyte recruitment and functional neonatal tendon regeneration. eLife *9*, e51779.

49. Lopez, R., Regier, J., Cole, M.B., Jordan, M.I., and Yosef, N. (2018). Deep generative modeling for single-cell transcriptomics. Nat. Methods *15*, 1053–1058.

50. Kleshchevnikov, V., Shmatko, A., Dann, E., Aivazidis, A., King, H.W., Li, T., Elmentaite, R., Lomakin, A., Kedlian, V., Gayoso, A., et al. (2022). Cell2location maps fine-grained cell types in spatial transcriptomics. Nat. Biotechnol. *40*, 661–671.

51. Birk, D.E., and Brückner, P. (2011). Collagens, Suprastructures, and Collagen Fibril Assembly. In The Extracellular Matrix: An Overview, R.P. Mecham, ed. (Berlin, Heidelberg: Springer), pp. 77–115. https://doi.org/10.1007/978-3-642-16555-9_3.

52. Docheva, D., Hunziker, E.B., Fässler, R., and Brandau, O. (2005). Tenomodulin Is Necessary for Tenocyte Proliferation and Tendon Maturation. Mol. Cell Biol. *25*, 699–705.

53. Dex, S., Lin, D., Shukunami, C., and Docheva, D. (2016). TENOgenic MODULating INsider factor: systematic assessment on the functions of tenomodulin gene. Gene *587*, 1–17.

54. Shukunami, C., Takimoto, A., Nishizaki, Y., Yoshimoto, Y., Tanaka, S., Miura, S., Watanabe, H., Sakuma, T., Yamamoto, T., Kondoh, G., and Hiraki, Y. (2018). Scleraxis is a transcriptional activator that regulates the expression of Tenomodulin, a marker of mature tenocytes and ligamentocytes. Sci. Rep. *8*, 3155.

55. Shukunami, C., Takimoto, A., Oro, M., and Hiraki, Y. (2006). Scleraxis positively regulates the expression of tenomodulin, a differentiation marker of tenocytes. Dev. Biol. *298*, 234–247.

56. Anderson, D.M., Arredondo, J., Hahn, K., Valente, G., Martin, J.F., Wilson-Rawls, J., and Rawls, A. (2006). Mohawk is a novel homeobox gene expressed in the developing mouse embryo. Dev. Dyn. *235*, 792–801.

57. Ito, Y., Toriuchi, N., Yoshitaka, T., Ueno-Kudoh, H., Sato, T., Yokoyama, S., Nishida, K., Akimoto, T., Takahashi, M., Miyaki, S., and Asahara, H. (2010). The Mohawk homeobox gene is a critical regulator of tendon differentiation. Proc. Natl. Acad. Sci. USA *107*, 10538–10542.

58. Liu, W., Watson, S.S., Lan, Y., Keene, D.R., Ovitt, C.E., Liu, H., Schweitzer, R., and Jiang, R. (2010). The Atypical Homeodomain Transcription Factor Mohawk Controls Tendon Morphogenesis. Mol. Cell Biol. *30*, 4797–4807.

59. Wang, W., Olson, D., Liang, G., Franceschi, R.T., Li, C., Wang, B., Wang, S.S., and Yang, S. (2012). Collagen XXIV (Col24α1) Promotes Osteoblastic Differentiation and Mineralization through TGF-β/Smads Signaling Pathway. Int. J. Biol. Sci. *8*, 1310–1322.

60. Takahata, Y., Hagino, H., Kimura, A., Urushizaki, M., Kobayashi, S., Wakamori, K., Fujiwara, C., Nakamura, E., Yu, K., Kiyonari, H., et al. (2021). Smoc1 and Smoc2 regulate bone formation as downstream molecules of Runx2. Commun. Biol. *4*, 1199.

61. He, Z., Zhu, Z., Tang, T., Wang, F., Guo, P., Li, J., Tung, N.T.C., Liang, Q., Liu, S., Gao, M., et al. (2024). Enpp1 mutations promote upregulation of hedgehog signaling in heterotopic ossification with aging. J. Bone Miner. Metab. *42*, 681–698.

62. Jenkins, E., Moss, J.B., Pace, J.M., and Bridgewater, L.C. (2005). The new collagen gene COL27A1 contains SOX9-responsive enhancer elements. Matrix Biol. *24*, 177–184.

63. Alcaide-Ruggiero, L., Molina-Hernández, V., Granados, M.M., and Domínguez, J.M. (2021). Main and Minor Types of Collagens in the Articular Cartilage: The Role of Collagens in Repair Tissue Evaluation in Chondral Defects. Int. J. Mol. Sci. *22*, 13329.

64. de Castro, L.F., Sworder, B.J., Mui, B., Futrega, K., Berendsen, A., Phillips, M.D., Burbach, N.J., Cherman, N., Kuznetsov, S., Gabet, Y., et al. (2021). Secreted frizzled related-protein 2 (Sfrp2) deficiency decreases adult skeletal stem cell function in mice. Bone Res. *9*, 49.

65. Bastepe, M. (2018). GNAS mutations and heterotopic ossification. Bone *109*, 80–85.

66. Kalamajski, S., Aspberg, A., Lindblom, K., Heinegård, D., and Oldberg, Å. (2009). Asporin competes with decorin for collagen binding, binds calcium and promotes osteoblast collagen mineralization. Biochem. J. *423*, 53–59.

67. Fan, R., Yan, X., and Zhang, W. (2022). Relationship between asporin and extracellular matrix behavior: A literature review. Medicine (Baltim.) *101*, e32490.

68. Maccarana, M., Svensson, R.B., Knutsson, A., Giannopoulos, A., Pelkonen, M., Weis, M., Eyre, D., Warman, M., and Kalamajski, S. (2017). Asporin-deficient mice have tougher skin and altered skin glycosaminoglycan content and structure. PLOS ONE *12*, e0184028.

69. Charvet, B., Guiraud, A., Malbouyres, M., Zwolanek, D., Guillon, E., Bretaud, S., Monnot, C., Schulze, J., Bader, H.L., Allard, B., et al. (2013). Knockdown of col22a1 gene in zebrafish induces a muscular dystrophy by disruption of the myotendinous junction. Development *140*, 4602–4613.

70. Koch, M., Schulze, J., Hansen, U., Ashwodt, T., Keene, D.R., Brunken, W.J., Burgeson, R.E., Bruckner, P., and Bruckner-Tuderman, L. (2004). A novel marker of tissue junctions, collagen XXII. J. Biol. Chem. *279*, 22514–22521.

71. Zhang, C.-H., Gao, Y., Hung, H.H., Zhuo, Z., Grodzinsky, A.J., and Lassar, A.B. (2022). Creb5 coordinates synovial joint formation with the genesis of articular cartilage. Nat. Commun. *13*, 7295.

72. Zhang, C.-H., Gao, Y., Jadhav, U., Hung, H.H., Holton, K.M., Grodzinsky, A.J., Shivdasani, R.A., and Lassar, A.B. (2021). Creb5 establishes the

CellPress
OPEN ACCESS

competence for Prg4 expression in articular cartilage. Commun. Biol. *4*, 332.

73. Hayashi, M., Zhao, C., Thoreson, A.R., Chikenji, T., Jay, G.D., An, K.N., and Amadio, P.C. (2013). The Effect of Lubricin on the Gliding Resistance of Mouse Intrasynovial Tendon. PLOS ONE *8*, e83836.

74. Thornton, G.M., Lemmex, D.B., Ono, Y., Beach, C.J., Reno, C.R., Hart, D.A., and Lo, I.K.Y. (2015). Aging affects mechanical properties and lubricin/PRG4 gene expression in normal ligaments. J. Biomech. *48*, 3306–3311.

75. Kohrs, R.T., Zhao, C., Sun, Y.L., Jay, G.D., Zhang, L., Warman, M.L., An, K.N., and Amadio, P.C. (2011). Tendon fascicle gliding in wild type, heterozygous, and lubricin knockout mice. J. Orthop. Res. *29*, 384–389.

76. Sun, Y.-L., Wei, Z., Zhao, C., Jay, G.D., Schmid, T.M., Amadio, P.C., and An, K.N. (2015). Lubricin in human achilles tendon: The evidence of intratendinous sliding motion and shear force in achilles tendon. J. Orthop. Res. *33*, 932–937.

77. Sun, Y., Berger, E.J., Zhao, C., Jay, G.D., An, K.N., and Amadio, P.C. (2006). Expression and mapping of lubricin in canine flexor tendon. J. Orthop. Res. *24*, 1861–1868.

78. Reuvers, J., Thoreson, A.R., Zhao, C., Zhang, L., Jay, G.D., An, K.N., Warman, M.L., and Amadio, P.C. (2011). The mechanical properties of tail tendon fascicles from lubricin knockout, wild type and heterozygous mice. J. Struct. Biol. *176*, 41–45.

79. Ostner, J., Carcy, S., and Müller, C.L. (2021). tascCODA: Bayesian Tree-Aggregated Analysis of Compositional Amplicon and Single-Cell Data. Front. Genet. *12*, 766405. https://doi.org/10.3389/fgene.2021.766405.

80. Zhang, B., He, P., Lawrence, J.E.G., Wang, S., Tuck, E., Williams, B.A., Roberts, K., Kleshchevnikov, V., Mamanova, L., Bolt, L., et al. (2024). A human embryonic limb cell atlas resolved in space and time. Nature *635*, 668–678. https://doi.org/10.1038/s41586-023-06806-x.

81. Grimaldi, A., Comai, G., Mella, S., and Tajbakhsh, S. (2022). Identification of bipotent progenitors that give rise to myogenic and connective tissues in mouse. eLife *11*, e70235.

82. Yamamoto, S., Uchida, Y., Ohtani, T., Nozaki, E., Yin, C., Gotoh, Y., Yakushiji-Kaminatsui, N., Higashiyama, T., Suzuki, T., Takemoto, T., et al. (2019). Hoxa13 regulates expression of common Hox target genes involved in cartilage development to coordinate the expansion of the autopodal anlage. Dev. Growth Differ. *61*, 228–251.

83. Bonnin, M.-A., Laclef, C., Blaise, R., Eloy-Trinquet, S., Relaix, F., Maire, P., and Duprez, D. (2005). Six1 is not involved in limb tendon development, but is expressed in limb connective tissue under Shh regulation. Mech. Dev. *122*, 573–585.

84. Wu, W., Huang, R., Wu, Q., Li, P., Chen, J., Li, B., and Liu, H. (2014). The Role of Six1 in the Genesis of Muscle Cell and Skeletal Muscle Development. Int. J. Biol. Sci. *10*, 983–989.

85. Nassari, S., Blavet, C., Bonnin, M.A., Stricker, S., Duprez, D., and Fournier-Thibault, C. (2017). The chemokines CXCL12 and CXCL14 differentially regulate connective tissue markers during limb development. Sci. Rep. *7*, 17279.

86. Stricker, S., Mathia, S., Haupt, J., Seemann, P., Meier, J., and Mundlos, S. (2012). Odd-skipped related genes regulate differentiation of embryonic limb mesenchyme and bone marrow mesenchymal stromal cells. Stem Cells Dev. *21*, 623–633.

87. La Manno, G., Soldatov, R., Zeisel, A., Braun, E., Hochgerner, H., Petukhov, V., Lidschreiber, K., Kastriti, M.E., Lönnerberg, P., Furlan, A., et al. (2018). RNA velocity of single cells. Nature *560*, 494–498.

88. Setty, M., Kiseliovas, V., Levine, J., Gayoso, A., Mazutis, L., and Pe'er, D. (2019). Characterization of cell fate probabilities in single-cell data with Palantir. Nat. Biotechnol. *37*, 451–460.

89. Lange, M., Bergen, V., Klein, M., Setty, M., Reuter, B., Bakhti, M., Lickert, H., Ansari, M., Schniering, J., Schiller, H.B., et al. (2022). CellRank for directed single-cell fate mapping. Nat. Methods *19*, 159–170.

90. Reuter, B., Klein, M., and Lange, M. (2022). pyGPCCA - python GPCCA: Generalized Perron Cluster Cluster Analysis package to coarse-grain reversible and non-reversible Markov state models. Zenodo. https://doi.org/10.5281/zenodo.6914001.

91. Weiler, P., Lange, M., Klein, M., Pe'er, D., and Theis, F. (2024). CellRank 2: unified fate mapping in multiview single-cell data. Nat. Methods *21*, 1196–1205.

92. Lotfollahi, M., Wolf, F.A., and Theis, F.J. (2019). scGen predicts single-cell perturbation responses. Nat. Methods *16*, 715–721.

93. Klein, D., Palla, G., Lange, M., Klein, M., Piran, Z., Gander, M., Meng-Papaxanthos, L., Sterr, M., Saber, L., Jing, C., et al. (2025). Mapping cells through time and space with moscot. Nature *638*, 1065–1075.

94. Fearon, A., Dahlstrom, J.E., Twin, J., Cook, J., and Scott, A. (2014). The Bonar score revisited: Region of evaluation significantly influences the standardized assessment of tendon degeneration. J. Sci. Med. Sport *17*, 346–350.

95. Khan, K.M., Cook, J.L., Bonar, F., Harcourt, P., and Åstrom, M. (1999). Histopathology of Common Tendinopathies. Sports Med. *27*, 393–408.

96. Maffulli, N., Longo, U.G., Franceschi, F., Rabitti, C., and Denaro, V. (2008). Movin and Bonar Scores Assess the Same Characteristics of Tendon Histology. Clin. Orthop. *466*, 1605–1611.

97. Broner, E.C., Trujillo, J.A., Korzinkin, M., Subbannayya, T., Agrawal, N., Ozerov, I.V., Zhavoronkov, A., Rooper, L., Kotlov, N., Shen, L., et al. (2021). Doublecortin-Like Kinase 1 (DCLK1) Is a Novel NOTCH Pathway Signaling Regulator in Head and Neck Squamous Cell Carcinoma. Front. Oncol. *11*, 677051.

98. Vijai, M., Baba, M., Ramalingam, S., and Thiyagaraj, A. (2021). DCLK1 and its interaction partners: An effective therapeutic target for colorectal cancer. Oncol. Lett. *22*, 850.

99. Kong, D., Wang, Z., Sarkar, S.H., Li, Y., Banerjee, S., Saliganan, A., Kim, H.R.C., Cher, M.L., and Sarkar, F.H. (2008). Platelet-Derived Growth Factor-D Overexpression Contributes to Epithelial-Mesenchymal Transition of PC3 Prostate Cancer Cells. Stem Cell. *26*, 1425–1435.

100. Folestad, E., Kunath, A., and Wågsäter, D. (2018). PDGF-C and PDGF-D signaling in vascular diseases and animal models. Mol. Aspects Med. *62*, 1–11.

101. Al-Hattab, D.S., Safi, H.A., Nagalingam, R.S., Bagchi, R.A., Stecy, M.T., and Czubryt, M.P. (2018). Scleraxis regulates Twist1 and Snai1 expression in the epithelial-to-mesenchymal transition. Am. J. Physiol. Heart Circ. Physiol. *315*, H658–H668.

102. Pryce, B.A., Brent, A.E., Murchison, N.D., Tabin, C.J., and Schweitzer, R. (2007). Generation of transgenic tendon reporters, ScxGFP and ScxAP, using regulatory elements of the scleraxis gene. Dev. Dyn. *236*, 1677–1682.

103. Schweitzer, R., Chyung, J.H., Murtaugh, L.C., Brent, A.E., Rosen, V., Olson, E.N., Lassar, A., and Tabin, C.J. (2001). Analysis of the tendon cell fate using Scleraxis, a specific marker for tendons and ligaments. Dev. Camb. Engl. *128*, 3855–3866.

104. Mezu-Ndubuisi, O.J., and Maheshwari, A. (2021). Role of macrophages in fetal development and perinatal disorders. Pediatr. Res. *90*, 513–523.

105. Epelman, S., Lavine, K.J., and Randolph, G.J. (2014). Origin and Functions of Tissue Macrophages. Immunity *41*, 21–35.

106. Muscat, S., Nichols, A.E.C., Gira, E., and Loiselle, A.E. (2022). CCR2 is expressed by tendon resident macrophage and T cells, while CCR2 deficiency impairs tendon healing via blunted involvement of tendon-resident and circulating monocytes/macrophages. FASEB J. *36*, e22607.

107. Wynn, T.A., Chawla, A., and Pollard, J.W. (2013). Origins and Hallmarks of Macrophages: Development, Homeostasis, and Disease. Nature *496*, 445–455.

108. Bielefeld, K.A., Amini-Nik, S., and Alman, B.A. (2013). Cutaneous wound healing: recruiting developmental pathways for regeneration. Cell. Mol. Life Sci. *70*, 2059–2081.

109. Little, M.H., and Kairath, P. (2017). Does Renal Repair Recapitulate Kidney Development? J. Am. Soc. Nephrol. *28*, 34–46.

110. Matsubayashi, Y., and Millard, T.H. (2013). Developmental Models for Wound Healing. In eLS (John Wiley & Sons, Ltd). https://doi.org/10.1002/9780470015902.a0021306.

111. Almekinders, L.C., and Deol, G. (1999). The effects of aging, antiinflammatory drugs, and ultrasound on the in vitro response of tendon tissue. Am. J. Sports Med. *27*, 417–421.

112. Delabastita, T., Bogaerts, S., and Vanwanseele, B. (2018). Age-related changes in achilles tendon stiffness and impact on functional activities: A systematic review and meta-analysis. J. Aging Phys. Act. *27*, 1–12.

113. Ippolito, E., Natali, P.G., Postacchini, F., Accinni, L., and De Martino, C. (1980). Morphological, immunochemical, and biochemical study of rabbit Achilles tendon at various ages. J. Bone Joint Surg. Am. *62*, 583–598.

114. Kannus, P., Paavola, M., and Józsa, L. (2005). Aging and Degeneration of Tendons. In Tendon Injuries: Basic Science and Clinical Medicine, N. Maffulli, P. Renström, and W.B. Leadbetter, eds. (Springer), p. 25. https://doi.org/10.1007/1-84628-050-8_4.

115. Nakagawa, Y., Majima, T., and Nagashima, K. (1994). Effect of ageing on ultrastructure of slow and fast skeletal muscle tendon in rabbit Achilles tendons. Acta Physiol. Scand. *152*, 307–313.

116. Strocchi, R., De Pasquale, V., Guizzardi, S., Govoni, P., Facchini, A., Raspanti, M., Girolami, M., and Giannini, S. (1991). Human achilles tendon: Morphological and morphometric variations as a function of age. Foot Ankle *12*, 100–104.

117. Birk, D.E., and Trelstad, R.L. (1986). Extracellular compartments in tendon morphogenesis: collagen fibril, bundle, and macroaggregate formation. J. Cell Biol. *103*, 231–240.

118. Humphries, S.M., Lu, Y., Canty, E.G., and Kadler, K.E. (2008). Active Negative Control of Collagen Fibrillogenesis in Vivo: INTRACELLULAR CLEAVAGE OF THE TYPE I PROCOLLAGEN PROPEPTIDES IN TENDON FIBROBLASTS WITHOUT INTRACELLULAR FIBRILS. J. Biol. Chem. *283*, 12129–12135.

119. Heinemeier, K.M., Schjerling, P., Heinemeier, J., Magnusson, S.P., and Kjaer, M. (2013). Lack of tissue renewal in human adult Achilles tendon is revealed by nuclear bomb 14C. FASEB J. *27*, 2074–2079.

120. Heinemeier, K.M., Schjerling, P., Øhlenschlæger, T.F., Eismark, C., Olsen, J., and Kjær, M. (2018). Carbon-14 bomb pulse dating shows that tendinopathy is preceded by years of abnormally high collagen turnover. FASEB J. *32*, 4763–4775.

121. Kalluri, R., and Weinberg, R.A. (2009). The basics of epithelial-mesenchymal transition. J. Clin. Investig. *119*, 1420–1428.

122. Alhajj, M., and Goyal, A. (2025). Physiology, Granulation Tissue. 2022 Oct 24. In StatPearls [Internet]. Treasure Island (FL) (StatPearls Publishing).

123. Hope, M., and Saxby, T.S. (2007). Tendon Healing. Foot Ankle Clin. *12*, 553–567.

124. Bruns, J., Kampen, J., Kahrs, J., and Plitz, W. (2000). Achilles tendon rupture: Experimental results on spontaneous repair in a sheep-model. Knee Surg. Sports Traumatol. Arthrosc. *8*, 364–369.

125. Sugimoto, Y., Takimoto, A., Akiyama, H., Kist, R., Scherer, G., Nakamura, T., Hiraki, Y., and Shukunami, C. (2013). Scx+/Sox9+ progenitors contribute to the establishment of the junction between cartilage and tendon/ligament. Dev. Camb. Engl. *140*, 2280–2288.

126. Blitz, E., Sharir, A., Akiyama, H., and Zelzer, E. (2013). Tendon-bone attachment unit is formed modularly by a distinct pool of Scx- and Sox9-positive progenitors. Development *140*, 2680–2690.

127. Zheng, G.X.Y., Terry, J.M., Belgrader, P., Ryvkin, P., Bent, Z.W., Wilson, R., Ziraldo, S.B., Wheeler, T.D., McDermott, G.P., Zhu, J., et al. (2017). Massively parallel digital transcriptional profiling of single cells. Nat. Commun. *8*, 14049.

128. Fleming, S.J., Chaffin, M.D., Arduini, A., Akkad, A.D., Banks, E., Marioni, J.C., Philippakis, A.A., Ellinor, P.T., and Babadi, M. (2023). Unsupervised removal of systematic background noise from droplet-based single-cell experiments using CellBender. Nat. Methods *20*, 1323–1335.

129. Aibar, S., González-Blas, C.B., Moerman, T., Huynh-Thu, V.A., Imrichova, H., Hulselmans, G., Rambow, F., Marine, J.C., Geurts, P., Aerts, J., et al. (2017). SCENIC: single-cell regulatory network inference and clustering. Nat. Methods *14*, 1083–1086.

130. Germain, P.-L., Lun, A., Meixide, C.G., Macnair, W., and Robinson, M.D. (2022). Doublet identification in single-cell sequencing data using *scDblFinder*. F1000Res. https://doi.org/10.12688/f1000research.73600.2.

131. Luecken, M.D., Büttner, M., Chaichoompu, K., Danese, A., Interlandi, M., Mueller, M.F., Strobl, D.C., Zappia, L., Dugas, M., Colomé-Tatché, M., and Theis, F.J. (2022). Benchmarking atlas-level data integration in single-cell genomics. Nat. Methods *19*, 41–50.

132. Gayoso, A., Lopez, R., Xing, G., Boyeau, P., Valiollah Pour Amiri, V., Hong, J., Wu, K., Jayasuriya, M., Mehlman, E., Langevin, M., et al. (2022). A Python library for probabilistic analysis of single-cell omics data. Nat. Biotechnol. *40*, 163–166.

133. Xu, C., Lopez, R., Mehlman, E., Regier, J., Jordan, M.I., and Yosef, N. (2021). Probabilistic harmonization and annotation of single-cell transcriptomics data with deep generative models. Mol. Syst. Biol. *17*, e9620.

134. Wolf, F.A., Angerer, P., and Theis, F.J. (2018). SCANPY: large-scale single-cell gene expression data analysis. Genome Biol. *19*, 15.

135. Palla, G., Spitzer, H., Klein, M., Fischer, D., Schaar, A.C., Kuemmerle, L.B., Rybakov, S., Ibarra, I.L., Holmberg, O., Virshup, I., et al. (2022). Squidpy: a scalable framework for spatial omics analysis. Nat. Methods *19*, 171–178.

136. Nowotschin, S., Setty, M., Kuo, Y.Y., Liu, V., Garg, V., Sharma, R., Simon, C.S., Saiz, N., Gardner, R., Boutet, S.C., et al. (2019). The emergent landscape of the mouse gut endoderm at single-cell resolution. Nature *569*, 361–367.

137. Raudvere, U., Kolberg, L., Kuzmin, I., Arak, T., Adler, P., Peterson, H., and Vilo, J. (2019). g:Profiler: a web server for functional enrichment analysis and conversions of gene lists (2019 update). Nucleic Acids Res. *47*, W191–W198.

138. Bankhead, P., Loughrey, M.B., Fernández, J.A., Dombrowski, Y., McArt, D.G., Dunne, P.D., McQuaid, S., Gray, R.T., Murray, L.J., Coleman, H.G., et al. (2017). QuPath: Open source software for digital pathology image analysis. Sci. Rep. *7*, 16878.

139. Schindelin, J., Arganda-Carreras, I., Frise, E., Kaynig, V., Longair, M., Pietzsch, T., Preibisch, S., Rueden, C., Saalfeld, S., Schmid, B., et al. (2012). Fiji: an open-source platform for biological-image analysis. Nat. Methods *9*, 676–682.

140. Schmidt, U., Weigert, M., Broaddus, C., and Myers, G. (2018). Cell Detection with Star-convex Polygons *11071*, 265–273. https://arxiv.org/abs/1806.03535.

141. Virtanen, P., Gommers, R., Oliphant, T.E., Haberland, M., Reddy, T., Cournapeau, D., Burovski, E., Peterson, P., Weckesser, W., Bright, J., et al. (2020). SciPy 1.0: fundamental algorithms for scientific computing in Python. Nat. Methods *17*, 261–272.

142. Seabold, S., and Perktold, J. (2010). Statsmodels: Econometric and Statistical Modeling with Python. scipy, 92–96. https://doi.org/10.25080/Majora-92bf1922-011.

143. Terpilowski, M. (2019). scikit-posthocs: Pairwise multiple comparison tests in Python. J. Open Source Softw. *4*, 1169.

144. Love, M.I., Huber, W., and Anders, S. (2014). Moderated estimation of fold change and dispersion for RNA-seq data with DESeq2. Genome Biol. *15*, 550.

145. Oliveros, J.C.V. (2015). An Interactive Tool for Comparing Lists with Venn's Diagrams (BioinfoGP Service).

146. Cribbs, A.P. (2024). cribbslab/scflow. cribbslab. https://github.com/cribbslab/cribbslab.

147. Sullivan, D.K., Min, K.H.J., Hjörleifsson, K.E., Luebbert, L., Holley, G., Moses, L., Gustafsson, J., Bray, N.L., Pimentel, H., Booeshaghi, A.S., et al. (2025). kallisto, bustools and kb-python for quantifying bulk, single-cell and single-nucleus RNA-seq. Nat. Protoc. *20*, 587–607.

148. Amezquita, R.A., Lun, A.T.L., Becht, E., Carey, V.J., Carpp, L.N., Geistlinger, L., Marini, F., Rue-Albrecht, K., Risso, D., Soneson, C., et al. (2020). Orchestrating single-cell analysis with Bioconductor. Nat. Methods *17*, 137–145.

149. Hao, Y., Hao, S., Andersen-Nissen, E., Mauck, W.M., 3rd, Zheng, S., Butler, A., Lee, M.J., Wilk, A.J., Darby, C., Zager, M., et al. (2021). Integrated analysis of multimodal single-cell data. Cell *184*, 3573–3587.e29.

150. McCarthy, D.J., Campbell, K.R., Lun, A.T.L., and Wills, Q.F. (2017). Scater: pre-processing, quality control, normalization and visualization of single-cell RNA-seq data in R. Bioinformatics *33*, 1179–1186.

151. Campbell, J., Yang, S., Wang, Z., Corbett, S. & Koga, Y. celda: CEllular Latent Dirichlet. https://doi.org/10.18129/B9.bioc.celda (2025).

152. Young, M.D., and Behjati, S. (2020). SoupX removes ambient RNA contamination from droplet-based single-cell RNA sequencing data. GigaScience *9*, giaa151.

153. Korsunsky, I., Millard, N., Fan, J., Slowikowski, K., Zhang, F., Wei, K., Baglaenko, Y., Brenner, M., Loh, P.R., and Raychaudhuri, S. (2019). Fast, sensitive and accurate integration of single-cell data with Harmony. Nat. Methods *16*, 1289–1296.

154. Büttner, M., Ostner, J., Müller, C.L., Theis, F.J., and Schubert, B. (2021). scCODA is a Bayesian model for compositional single-cell data analysis. Nat. Commun. *12*, 6876.

155. Pedregosa, F., et al. (2011). Scikit-learn: Machine Learning in Python. J. Mach. Learn. Res. *12*, 2825–2830.

156. Lemaître, G., Nogueira, F., and Aridas, C.K. (2017). Imbalanced-learn: A Python Toolbox to Tackle the Curse of Imbalanced Datasets in Machine Learning. J. Mach. Learn. Res. *18*, 1–5.

157. Xu, C., Prete, M., Webb, S., Jardine, L., Stewart, B.J., Hoo, R., He, P., Meyer, K.B., and Teichmann, S.A. (2023). Automatic cell-type harmonization and integration across Human Cell Atlas datasets. Cell *186*, 5876–5891.e20.

158. Szklarczyk, D., Kirsch, R., Koutrouli, M., Nastou, K., Mehryary, F., Hachilif, R., Gable, A.L., Fang, T., Doncheva, N.T., Pyysalo, S., et al. (2023). The STRING database in 2023: protein–protein association networks and functional enrichment analyses for any sequenced genome of interest. Nucleic Acids Res. *51*, D638–D646.

159. Mimpen, J., Paul, C., Network, T. S., Cribbs, A. & Snelling, S. Nuclei isolation from snap-frozen tendon tissue for single nucleus RNA Sequencing. https://www.protocols.io/view/nuclei-isolation-from-snap-frozen-tendon-tissue-fo-bc6xizfn (2021).

160. Baldwin, M.J., Cribbs, A.P., Guilak, F., and Snelling, S.J.B. (2021). Mapping the musculoskeletal system one cell at a time. Nat. Rev. Rheumatol. *17*, 247–248.

161. Baldwin, M., Buckley, C.D., Guilak, F., Hulley, P., Cribbs, A.P., and Snelling, S. (2023). A roadmap for delivering a human musculoskeletal cell atlas. Nat. Rev. Rheumatol. *19*, 738–752.

162. Heumos, L., Schaar, A.C., Lance, C., Litinetskaya, A., Drost, F., Zappia, L., Lücken, M.D., Strobl, D.C., Henao, J., Curion, F., et al. (2023). Best practices for single-cell analysis across modalities. Nat. Rev. Genet. *24*, 550–572.

163. Bergen, V., Lange, M., Peidli, S., Wolf, F.A., and Theis, F.J. (2020). Generalizing RNA velocity to transient cell states through dynamical modeling. Nat. Biotechnol. *38*, 1408–1414.

164. Abdulla, S., Aevermann, B., Assis, P., Badajoz, S., Bell, S.M., Bezzi, E., Cakir, B., Chaffer, J., Chambers, S., et al.; CZI Single-Cell Biology Program (2023). CZ CELL×GENE Discover: A single-cell data platform for scalable exploration, analysis and modeling of aggregated data. bioRxiv. https://doi.org/10.1101/2023.10.30.563174.

165. Mao, S., Zhang, Y., Seelig, G., and Kannan, S. (2022). CellMeSH: probabilistic cell-type identification using indexed literature. Bioinformatics *38*, 1393–1402.

166. Ahlmann-Eltze, C., and Huber, W. (2023). Comparison of transformations for single-cell RNA-seq data. Nat. Methods *20*, 665–672.

STAR★METHODS

KEY RESOURCES TABLE

REAGENT or RESOURCE	SOURCE	IDENTIFIER
Antibodies		
PECAM1 (C31.3; AF647)	Novus Biologicals, Bio-techne	Cat#NBP2-33154; RRID: AB_3283994
CD163 (EdHu-1; AF647)	Novus Biologicals, Bio-techne	Cat# NB110-40686; RRID: AB_714951
Biological samples		
Human fetal tissues	MRC/Wellcome Trust Human Developmental Biology Resource (HDBR)	MTA license R60786/CN008, REC 18/LO/0822; grant #MR/R006237/1; www.hdbr.org
Human adult tendon tissues	Oxford Musculoskeletal Biobank	Ethics approval 19/SC/0134; https://www.hra.nhs.uk/planning-and-improving-research/application-summaries/research-summaries/oxford-musculoskeletal-biobank/
Chemicals, peptides, and recombinant proteins		
Water, RNase and DNase free	Invitrogen	Cat#10977-035
D-PBS (phosphate-buffered saline)	Sigma	Cat#D8537
2M NaCl	Merck	Cat#S9888
1M Tris-HCL pH 7.5	ThermoFisher Scientific	Cat#1013203
1M CaCl2	Merck	Cat#383147
1M MgCl2	Merck	Cat#M8266
CHAPS hydrate	Sigma	Cat#C3023
BSA (bovine serum albumin)	Sigma	Cat#A7030
cOmplete tablets	Roche	Cat#5892791001
RNaseIn Plus	Promega	Cat#N2615
SUPERase In	Invitrogen	Cat#AM2696
OCT mounting medium	VWR	Cat#00411243
DAPI	ThermoFisher Scientific	Cat#62248
Picrosirius Red	Abcam	Cat#ab246832
Alcian blue	Atom Scientific	Cat#RRSK300-1000
Massons Trichrome	Cell Path	Cat#RBK-0601-00B
Haematoxylin	Leica Microsystems	Cat#3801560E
Eosin Y 1% aqueous	Cell Path	Cat#RBC-0100-00A
Critical commercial assays		
Visium Gene Expression Slide and Reagent Kit	10x Genomics	Cat#1000187
Chromium Next GEM Single Cell 3′ Kit v3.1	10x Genomics	Cat#1000269
Chromium Next GEM Chip G Single Cell Kit	10x Genomics	Cat#1000120
Dual Index Kit TT Set A (for Visium ST)	10x Genomics	Cat#1000215
Single Index Kit T Set A (for snRNA-seq)	10x Genomics	Cat#1000213
High Sensitivity D1000 DNA ScreenTape	Agilent	Cat#5067-5582
High Sensitivity D1000 DNA ScreenTape Reagents	Agilent	Cat#5067-5583
High Sensitivity D5000 DNA ScreenTape	Agilent	Cat#5067-5588
High Sensitivity D5000 DNA ScreenTape Reagents	Agilent	Cat#5067-5589

(*Continued on next page*)

Continued

REAGENT or RESOURCE	SOURCE	IDENTIFIER
Deposited data		
Human embryonic whole limb single-cell RNA-sequencing data	Zhang et al.[80]	[ArrayExpress]: [E-MTAB-8813] [https://www.ebi.ac.uk/biostudies/arrayexpress/studies/E-MTAB-8813]
Human embryonic whole limb spatial RNA-sequencing data	Zhang et al.[80]	[ArrayExpress]: [E-MTAB-10367] [https://www.ebi.ac.uk/biostudies/arrayexpress/studies/E-MTAB-10367]
Human fetal tendon single-nucleus RNA-sequencing data	This study	Processed data: [CellxGene] : [https://cellxgene.cziscience.com/collections/7b9ae565-a781-433d-98d4-430394e7802a]<br><br>Raw data: [GEO]: [GSE317327]
Human fetal tendon spatial RNA-sequencing data	This study	[Zenodo]: [http://www.doi.org/10.5281/zenodo.18403632]
Human adult Achilles tendon single-nucleus RNA-sequencing data	Cohen et al. (in press)	Available upon request following publication.
Human adult quadriceps tendon single-nucleus RNA-seq data	Mimpen et al.[30]	Processed data: [CellxGene]: [https://cellxgene.cziscience.com/collections/579203e2-182f-47bc-8230-7aa47247e2a4]
Software and algorithms		
10x Genomics CellRanger v7.0	Zheng et al.[127]	https://www.10xgenomics.com/support/software/cell-ranger/latest
10x Genomics CellBender v0.2.2	Fleming et al.[128]	https://github.com/broadinstitute/CellBender
10x Genomics SpaceRanger v1.3.1	10X Genomics	https://www.10xgenomics.com/support/software/space-ranger/latest
Loupe Browser v6.4.1	10X Genomics	https://www.10xgenomics.com/support/software/loupe-browser/latest
pySCENIC v0.12.1	Aibar et al.[129]	https://scenic.aertslab.org
velocyto v0.17.17	La Manno et al.[87]	https://velocyto.org
scDblFinder v1.4.0	Pierre-Luc Germain et al.[130]	https://github.com/plger/scDblFinder
scVelo v0.2.5	Weiler et al.[91]	https://scvelo.readthedocs.io/en/stable/
scIB v1.1.3	Luecken et al.[131]	https://github.com/theislab/scib
scvi-tools v0.16.1	Gayoso et al.[132]	https://scvi-tools.org
scVI (part of scvi-tools)	Lopez et al.[49]	https://github.com/scverse/scvi-tools
scANVI (part of scvi-tools)	Xu et al.[133]	https://docs.scvi-tools.org/en/1.3.0/user_guide/models/scanvi.html
scanpy<br>v1.7.2 (fetal snRNA-seq data)<br>v1.9.5 (fetal spatial RNA-seq data)	Wolf, Angerer and Theis[134]	https://scanpy.readthedocs.io/en/stable/
squidpy v1.2.3	Palla et al.[135]	https://squidpy.readthedocs.io/en/stable/
cell2location v0.1.4	Kleshchevnikov et al.[50]	https://cell2location.readthedocs.io/en/latest/
Harmony v0.1.4	Nowotschin et al.[136]	https://github.com/dpeerlab/Harmony
Palantir v1.3.1	Setty et al.[88]	https://github.com/dpeerlab/Palantir
CellRank v1.5.1 (fetal data)<br>v2.0.2 (embryonic and fetal combined)	Lange et al.[89];<br>Weiler et al.[91]	https://cellrank.readthedocs.io/en/latest/
gprofiler g:GOSt	Raudvere et al.[137]	https://biit.cs.ut.ee/gprofiler/gost
QuPath v0.4.3 (image quantification)<br>v0.5.1 (immunofluorescence image analysis)	Bankhead et al.[138]	https://qupath.github.io
Fiji ImageJ v1.53t	Schindelin et al.[139]	https://imagej.net/software/fiji/
StarDist ImageJ Plugin v0.3.0	Schmidt et al.[140]	https://imagej.net/plugins/stardist
scipy v1.11.1	Virtanen et al.[141]	https://scipy.org

(*Continued on next page*)

Continued		
REAGENT or RESOURCE	SOURCE	IDENTIFIER
statsmodels v0.14.0	Seabold and Perktold[142]	https://www.statsmodels.org/stable/
Scikit-posthocs v0.9.0	Terpilowski[143]	scikit-posthocs.rtfd.io
DESeq2 v1.40.2	Love, Huber and Anders[144]	https://www.bioconductor.org/packages/release/bioc/html/DESeq2.html
Venny v2.1.0	Oliveros[145]	https://bioinfogp.cnb.csic.es/tools/venny/
scflow (custom development version)	Cribbs et al.[146] (unpublished)	https://github.com/cribbslab/scflow
kallisto bustools v0.27.3	Sullivan et al.[147]	https://www.kallistobus.tools/kb_usage/-kb_count/
SingleCellExperiment v1.22.0	Amezquita et al.[148]	https://www.bioconductor.org/packages/release/bioc/html/SingleCellExperiment.html
Seurat v4.0.3.1	Hao et al.[149]	https://satijalab.org/seurat/
scuttle v1.10.1	McCarthy et al.[150]	https://doi.org/10.1093/bioinformatics/btw777
celda v1.14.0	Campbell et al.[151]	https://bioconductor.org/packages/celda
SoupX v1.6.2	Young and Behjati[152]	https://github.com/constantAmateur/SoupX
Harmony v0.1	Korsunsky et al.[153]	https://portals.broadinstitute.org/harmony/articles/quickstart.html
R v4.3.1	R Core Team (2021). R: A Language and Environment for Statistical Computing, Vienna, Austria.	http://www.R-project.org/
RStudio v2023.03.1	RStudio Team (2020). RStudio: Integrated Development for R. RStudio, PBC, Boston, MA.	http://www.rstudio.com/
scCODA v0.1.9	Büttner et al.[154]	https://github.com/theislab/scCODA
scikit-learn v1.3.0	Pedregosa et al.[155]	http://jmlr.org/papers/v12/pedregosa11a.html
Imbalanced-learn v0.12.0	Lemaître et al.[156]	http://jmlr.org/papers/v18/16-365.html
scGen v2.1.1	Lotfollahi, Wolf and Theis[92]	https://github.com/theislab/scgen
moscot v0.3.3	Klein et al.[93]	https://moscot-tools.org
CellHint v1.0.0	Xu et al.[157]	https://github.com/Teichlab/cellhint
stringdb v.12.0	Szklarczyk et al.[158]	https://string-db.org
Other		
Analysis code	This paper	https://github.com/AlinaKurjan/DPhilCode Zenodo DOI: 10.5281/zenodo.18379150
10x Genomics GRCh38-2020-A human reference genome file (refdata-gex-GRCh38-2020-A/genes/genes.gtf)	10X Genomics	https://www.10xgenomics.com/support/software/cell-ranger/downloads#reference-downloads
h38_repeat_mask.gtf	UCSC Genome Browser	https://genome.ucsc.edu/cgi-bin/hgTables?hgsid=611454127_NtvlaW6xBSIRYJEBI0iRDEWisITa&clade=mammal&org=0&db=0&hgta_group=genes&hgta_track=refSeqComposite&hgta_table=ncbiRefSeq&hgta_regionType=genome&position=&hgta_outputType=gff&hgta_outFileName=mm10_rmsk.gtf
allTFs_hg38.txt file	Aertslab SCENICprotocol Github Repository	https://github.com/aertslab/SCENICprotocol/tree/master/example
Genome rankings (.feather) and motif annotation (.tbl) files, v10	Aertslab cisTarget resources	https://resources.aertslab.org/cistarget/

EXPERIMENTAL MODEL AND STUDY PARTICIPANT DETAILS

Human fetal tissue samples

Human fetal material was supplied by the Joint MRC/Wellcome Trust (grant #MR/R006237/1) Human Developmental Biology Resource (HDBR, www.hdbr.org) under the MTA license (R60786/CN008) with REC 18/LO/0822. Fresh fetal lower limbs aged 9–20 post-conception weeks (pcw) were provided by the HDBR London UCL Institute of Child Health. Fetal age was estimated using the independent measurement of the crown rump length (CRL), using the formula PCW (days) = 0.9022 3 CRL (mm) + 27.372 and then rounding up to the full decimal. All samples used in this work were sourced from elective terminations with no abnormalities recorded. Fetal sample ages in post-conception weeks were as follows: DEV16134 – 12, DEV16135 – 12, DEV16171 – 12, DEV16136 – 12, DEV16127 – 17, DEV16569 – 17, DEV15983 – 20, DEV15984 – 20, DEV15985 – 20, DEV16126 – 20. DEV16126 was used for Visium spatial RNA-sequencing, while the remaining samples were used for single-nucleus RNA-sequencing. Sample metadata is summarized in Table S1.

Human adult tissue samples

Ethical approval for the Oxford Musculoskeletal Biobank (OMB, 19/SC/0134) was granted by the Oxford Research Ethics Committee B for all work on human Achilles and quadriceps tendons. Written informed consent according to the Declaration of Helsinki was obtained from all patients. Healthy Achilles and quadriceps tendon tissues were collected from patients undergoing above- or below-the-knee amputations (e.g., OMB0785) or suprapatellar nailing of tibial shaft fracture (e.g., OMB1266). Ruptured quadriceps tendon samples were obtained from patients with acute full quadriceps tendon ruptures, with surgeries performed 8 to 9 days post-rupture. Donors with diabetes or rheumatologic disease were excluded from this study. Patient ages in years were as follows: healthy Achilles tendon (OMB0785 – 74, OMB1556 – 51, OMB1250 – 45, OMB1691 – 58, OMB1687 – 76); healthy quadriceps tendon (OMB0792 – 29, OMB1266 – 25, OMB1248 – 44); ruptured quadriceps tendon (OMB0778 – 67, OMB0793 – 69, OMB0779 – 75). Sample metadata is summarized in Table S1.

METHOD DETAILS

Fetal and adult tendon tissue processing

The tissues were washed in PBS (phosphate-buffered saline) and dissected to retain the regions of interest – whole tendons for fetal tissues, including muscle and bone attachment sites; enthesis, midbody, and MTJ regions (∼1cm pieces) for adult tissues – using anatomical landmarks. Adult tissue cuts were photographed to retain topographical reference.

The tissues were then either formalin-fixed for staining or snap-frozen in liquid nitrogen for sequencing as soon as possible after collection. Following formalin-fixation (with different lengths of time depending on the size of the samples), the samples were resuspended in 70% IMS (industrial methylated spirit) and stored at room temperature. After dehydration, formalin-fixed tissues were paraffin-embedded and sectioned. Snap-frozen tissues were stored at −70°C to −80°C before being used for single-nuclei or spatial RNA-sequencing.

Fetal and adult tendon nuclei isolation

Nuclei isolation for fetal and adult tendon samples outlined in Table S1 was carried out following protocols for large (adult) and small (fetal) tissues developed and published by Mimpen and colleagues from the Tendon Seed Network.[159] Briefly, tendons were cut into ∼1 mm pieces on dry ice and dissociated in CST buffer (292 mM NaCl, 20 mM Tris-HCL 7.5 pH, 2 mM CaCl2, 42 mM MgCl2, 0.5% CHAPS, 0.01% BSA, RNase and protease inhibitors) on a rotor for 2 or 10 min at 4°C. After addition of PBS with 2% or 1% BSA (bovine serum albumin) the suspensions were strained through 20 or 40 μm strainers (Greiner Bio-one), with former numbers used for fetal and latter for adult tissues. The nuclei-containing suspensions were then centrifuged at 500 g for 5 min at 4°C. Following centrifugation, the supernatant was discarded and the nuclei within the pellet were stained with DAPI and counted manually using a haemocytometer (NanoEntek DHC-N01) and fluorescence microscopy.

Fetal and adult tendon single-nucleus RNA-sequencing

Nuclear suspensions, diluted in PBS with 1% BSA to a concentration of 200–1000 nuclei/μL, were loaded onto a Chromium Next GEM Chip G (10x Genomics) with the aim of recovering 1,000–10,000 nuclei per sample. The samples were then processed using the Chromium Controller (10x Genomics) and prepared into libraries using the Chromium Next GEM Single Cell 3′ Reagent Kits v3.1 (10x Genomics) following the manufacturer's instructions. Libraries were indexed with the Single Index Kit T Set A (10x Genomics). Quality control assessments for cDNA and final libraries were conducted using D1000 or D5000 High Sensitivity ScreenTape (Agilent) assays on a 4150 TapeStation System (Agilent). The final libraries were pooled together and sequenced using a NovaSeq 6000 (Illumina) by Genewiz (UK) with a minimum sequencing depth of approximately 20,000 read pairs per expected nucleus.

Adult tendon snRNA-seq data processing

Human adult tendon single-nuclei RNA-sequencing datasets (N = 26 libraries from 12 donors) were processed, integrated and annotated by Dr Carla Cohen (Achilles tendon) and Dr Jolet Y. Mimpen (quadriceps tendon; published in [30]) within the framework of the

Tendon Seed Network[160,161] (Oxford, UK). Briefly, raw sequencing files were processed using scflow[146] (custom development version; pipeline *scflow quantnuclei*), with reads mapped to the human Ensembl GRCh38 transcriptome (release 106) using kallisto bustools[147] (v0.27.3). Single-nucleus RNA-seq analysis and annotation was performed in R (v4.3.1) and RStudio Server (v2023.03.1, build 446) using SingleCellExperiment[148] (v1.22.0) and Seurat[149] (v4.3.0.1) packages. The counts were log-normalized using Seurat's default functions. QC metrics were calculated with scuttle[150] (v1.10.1). Filtering thresholds for number of cells, number of features and mitochondrial ratios were set manually for each sample to remove poor-quality cells. Doublets were detected and removed with scDblFinder[130] (v1.12.0) using default settings. Ambient RNA was detected using *decontX()* from celda[151] (v1.14.0). Further ambient RNA detection was performed using SoupX [134] (v1.6.2), and the soupX-adjusted count matrix was used for downstream analysis. Integration of samples was performed using Harmony[162] (v0.1), with a combined sample donor and tissue type variable specified for batch correction. Clusters were defined with Seurat's *FindClusters()*, and a cluster comprising nuclei with high decontX scores was removed. Annotation was performed by assessing cluster-specific expression of manually curated gene sets.

Fetal tendon snRNA-seq data processing

Fetal tendon raw sequencing files were aligned with CellRanger[155] (v7.0) using 10x Genomics' pre-built GRCh38-2020-A reference (compiled from ENSEMBL's 98th release of the human reference genome) with default settings. Ambient RNA was removed using CellBender[128] (v0.2.2) with custom values for expected-cells, total-droplets-included, epochs and low-count-threshold parameters, which were selected based on the properties of individual samples and what was expected. The command was rerun until optimal conditions (training and test loss converging, expected number of cells selected, no error or warning messages in log files) were achieved for each sample. To obtain spliced and unspliced count matrices, velocyto[87] (v0.17.17) command line function *velocyto run10x* was used with the aforementioned reference genome file and a repeat sequences masked gtf file (h38_repeat_mask.gtf) downloaded from UCSC Genome Browser. The resulting loom output files were compared with CellBender output files, then merged using scVelo[163] (v0.2.5) function *scvelo.utils.merge()* to retain overlapping barcodes.

Data processing and analysis were carried out using Python and R packages. Briefly, merged files were filtered to remove: 1) genes that were detected in fewer than 20 cells (using Scanpy[157] (v1.7.2) function *scanpy.pp.filter_genes(adata, min_cell=20))*, 2) genes with 0 UMI counts, and 3) cells with fewer than 200 UMI counts. Following this basic filtering, low quality reads were removed using permissive automatic thresholding based on median absolute deviations (MAD), as described in single-cell best practices guidelines.[129] Cells were marked as outliers and filtered out if they differed by 5 MADs in their logarithmical total counts, genes-by-counts, and percentage counts in top 20 genes. Additionally, cells with more than 10% mitochondrial counts and 3 MADs were also removed. Doublets were removed individually for each sample using scDblFinder[156] (v1.4.0) with default settings. Finally, QC plots were analyzed to determine the necessity of additional filtering thresholds for each individual sample. The cells were then filtered by manually defined minimal genes-by-counts thresholds that ranged from 200 to 500.

For normalisation, the filtered and concatenated anndata object was split into separate adult-only and developmental-only sample objects. Each was normalized using a shifted logarithm approach based on the delta method (referred to as log1pPF) involving *scanpy.pp.normalize_total(target_sum=None)* followed by *scanpy.pp.log1p()*, as recommended by the recent comprehensive data transformation benchmark study.[158] Top 3,500 highly variable genes were selected for adult-only and developmental-only objects using scIB[154] (v1.1.3) package's *scib.preprocessing.hvg_batch()* function, with *flavor* and *batch_key* parameters set to 'cell_ranger' and 'sampletype', respectively. Additional filtering was done to remove 51 genes detected in less than 5 counts and remove counts with fewer than 200 genes expressed. Cell cycle phase was determined using *scib.preprocessing.score_cell_cycle()*. The data were then scaled using a custom function *split_and_scale()* that split the concatenated objects by 'sampletype' and applied *scanpy.pp.scale()* individually for each sample. Dimensionality reduction in the form of principal component analysis (PCA) was then applied to scaled highly variable genes, and neighbors (*n_neighbors=30, npcs=15*) were calculated to produce uniform manifold approximation (UMAP) plots to assess the quality control steps.

Fetal sample data were integrated and batch-corrected using scvi-tools[138] (v0.16.1) package's single-cell variational inference (scVI)[49] modeling on all unnormalized gene counts, with the main batch effects of interest specified to be 'sampletype' and 'libbatch', corresponding to different donor and tissue type (e.g., DonorID1_AchillesTendon and DonorID1_QuadTendon) and library preparation batches, respectively. Model hyperparameters were optimised using manual runs with different parameters as well as using scVI's autotune functionality, which determined the best fitting parameters to be 'n_latent': 30, 'n_layers': 2, 'dropout_rate': 0.1, 'gene_likelihood': 'zinb', 'dispersion': 'gene-batch'. The model was trained using 398 epochs until model training and validation sets were stably converged.

Data clustering was carried out using Scanpy's Leiden algorithm, identifying a total of 19 clusters (0–18) at 0.6 resolution. Normalized and log-transformed cluster gene counts were ranked using a Wilcoxon rank-sum test with *scanpy.tl.rank_genes_groups().* The clusters were manually annotated by checking known cell type markers and by querying top 350–550 Leiden cluster DEGs with CellMESH[140] and gProfiler's g:GOSt functional profiling[159] tools. Heatmaps and hierarchical clustering dendrograms were also consulted in the process to make the best guesses for the previously undefined cell types. Following scVI integration and cell type labeling, scANVI[141] was run with the scVI model as basis for 25 epochs. The resulting latent representation embeddings were used for the computation of a neighborhood graph of observations, producing annotated data UMAPs.

10X visium spatial RNA-sequencing of fetal samples

Fetal Achilles (*N=1* sample) and quadriceps tendons (*N=2* samples) (Table S1) were dissected from both legs of a single 20 pcw fetus and flash frozen in liquid nitrogen. In preparation for spatial transcriptomics (ST), the samples were cut to $\leq$0.65 cm^2 to fit the 10x Genomics Visium ST slide regions. We were able to retain enthesis-to-MTJ as well as adjacent muscle tissue regions for both types of tendons. The samples were embedded in cold OCT mounting medium (VWR) on dry ice and cut longitudinally into 10 μm sections, which were then fixed and stained with H&E to verify tissue morphology and suitability. The sections were prepared for sequencing according to the 10X Genomics recommended protocols using the Visium Gene Expression Slide and Reagent Kit (3′ v1) alongside a Dual Index Kit TT Set A. Libraries were sequenced using Illumina NextSeq500 (paired-end) at a depth of 54,000 (Quad2 tendon), 74,000 (Ach) and 119,000 (Quad1) mean reads per spot. The data and images were processed with SpaceRanger (v1.3.1; 10X Genomics) using default settings and mapped to the GRCh38 reference genome.

Fetal spatial RNA-seq data processing

The data were processed using Scanpy[157] (v1.9.5) and Squidpy[142] (v1.2.3). Briefly, tissue objects were manually filtered to remove: 1) cells with fewer than 500–1,000 counts or more than 10,000–20,000 counts (with exact numbers depending on individual sample properties), 2) genes that were detected in fewer than 10 cells, and 3) any ribosomal and mitochondrial reads. The counts were then normalized to log1pPF, and 2,000 highly variable genes were selected with the “cell_ranger" flavor using *scanpy.pp.highly_variable_genes()*. After normalized count scaling with *scanpy.pp.scale()*, PCA was carried out, and neighbors and UMAPs were calculated for the data using default Scanpy functions. Finally, all tissue objects were subjected to Leiden clustering at 1.0 resolution, identifying 7 clusters for each of the samples. The clusters were examined by analysing the outputs of a Wilcoxon rank-sum test run using *scanpy.tl.rank_genes_groups()*.

Fetal cell type mapping to spatial coordinates

To infer the spatial distribution of cell types within the tissue, processed and annotated fetal snRNA-seq data were integrated with spatial RNA-seq information using cell2location[50] (v0.1.4). Cell2location hyperparameters were specified based on the tissue and experiment considerations in mind as having a) expected cell abundance per Visium spot set to 17 (average from a range of 10–32 nuclei in different regions), and b) regularisation of within-experiment variation in RNA detection sensitivity set to 20. The model was trained until convergence for a total of 16,000 iterations.

H&E images were used as basis for microanatomical tissue region identification and labeling. To further analyze potential tissue microenvironments in an unsupervised manner, non-negative matrix factorization (NMF) using cell2location’s scikit-learn NMF wrapper function was also applied to estimated cell abundances. The model was trained using a range of factors (5–30) for decomposition of cell abundance data, with smaller factors assuming lower numbers of distinct patterns and higher likelihood of cell colocation.

Immunofluorescence staining and imaging of fetal tendons

Snap-frozen tendon samples were embedded in OCT (VWR) and sectioned at 7 μm thickness. All staining procedures followed the Cell DIVE Platform protocol (GE Research, Niskayuna, NY, USA). Tissue sections were post-fixed for 1 min at 4°C in a 1:1 ethanol-acetone solution, then blocked overnight at 4°C in PBS containing 3% BSA and 10% donkey serum (Bio-Rad).

Slides were stained with DAPI (ThermoFisher) and mounted using an antifade medium containing 4% propyl gallate and 50% glycerol (Sigma-Aldrich). Initial imaging at 20X magnification was performed to capture background autofluorescence, which was subtracted from subsequent staining rounds. Following this, coverslips were removed in PBS, and slides were incubated with antibodies overnight at 4°C. After incubation, slides were washed three times in PBS (5 min each with gentle agitation), re-coverslipped, and imaged.

A bleaching step was then performed by decoverslipping and incubating the slides three times for 15 min in 0.5 M $NaHCO_3$ (pH 11.2) containing 3% H_2O_2, with 1-min PBS washes between each bleach. This was followed by three additional PBS washes and a 2-min DAPI recharge. Slides were then re-coverslipped, and a bleached image was acquired for subtraction from the next staining round. Image analysis was conducted using QuPath (v0.5.1).

Histochemistry of fetal and adult tendon tissues

Histochemical staining of fetal and adult sectioned formalin-fixed tissues was performed by the Histology Team at the Kennedy Institute of Rheumatology. The slides were stained with haematoxylin and eosin (H&E), Alcian blue, masson’s trichrome, and picrosirius red. Slides were scanned with Motic EasyScan One. PSR-stained slides were additionally imaged under polarized light using an Olympus BX40.

Embryonic whole limb data processing

Single-cell RNA-sequencing (*N* = 25 libraries from different hindlimb regions of 11 donors) and 10X Visium spatial RNA-sequencing (*N* = 8 libraries from different hindlimb regions of two 6pcw and one 8pcw donors) files were provided by the Teichmann group at the Wellcome Sanger Institute (Cambridge, UK) (Table S1). The tissues were collected, processed and sequenced by the group in accordance with the methods published by Zhang et al.[80] For this analysis, the raw sequencing files were processed and aligned in the

same way as was done for the fetal samples to minimise bias, using same versions of the CellRanger and CellBender packages for the single-cell data and the SpaceRanger package for the spatial data.

Human embryonic spatial RNA-seq data were analyzed using Loupe Browser (v6.4.1; 10X Genomics). Expression of early tenocyte markers (e.g., *SCX*, *MKX*, *FMOD*, *TNMD*, *EGR1* etc.) and H&E-stained sections guided tendon tissue annotation. While tendon regions could not be confidently defined in 6 pcw samples, a merged 8 pcw hindlimb sample enabled identification of developing patellar and quadriceps tendon areas (Figure S1). Of 2,439 Visium spots, 28 were annotated as tendon: 18 patellar and 10 quadriceps (Figure S1).

Single-cell embryonic data samples from 6 to 9 pcw (*N* = 25, total of 108,617 cells) were processed and integrated using scvi-tools' scVI modeling on all unnormalized gene counts, with the main batch effects of interest specified to be 'samplename' (consisting of Sample ID), 'kit', 'seq_protocol', and 'sex' (Figure S2A). As before, model hyperparameters were optimised with scVI autotune and manual runs to 'n_hidden': 256, 'n_latent': 14, 'n_layers': 3, 'dropout_rate': 0.1, 'gene_likelihood': 'nb', and 'dispersion': 'gene-batch'. The model was trained using 394 epochs until training and validation sets were stably converged. The resulting latent representation embeddings were then used as basis for the computation of a neighborhood graph of observations and dimensionality reduction with UMAP.

To identify tendon cell subsets in embryonic whole-limb scRNA-seq data, a random forest classifier was trained using spatial transcriptomics as a reference. Spatial Visium data were first divided into 28 tendon and 2,411 non-tendon spots. To address the small sample size, "pseudodonors" were created by assigning each tendon and an equal number of random non-tendon spots unique identifiers, while the remaining non-tendon spots were pooled into a single 29th pseudodonor. The *aggregate_and_filter()* function from Heumos et al.[143] was adapted to generate 25 pseudoreplicates per donor based on estimated cell counts per spot. These were aggregated into a spatial reference AnnData object containing 1,425 observations and 14,208 genes. Both spatial and scRNA-seq data were log1pPF-normalized and concatenated to identify 5,000 highly variable genes (flavor = "cell_ranger", batch_key = "modality" ("spatial" vs. "single-cell")).

To train the random forest classifier, class imbalances of tendon vs. non-tendon data were addressed using Synthetic Minority Over-sampling Technique (SMOTE) from imbalanced-learn[132] (v0.12.0) package (Lemaître et al., 2017). Spatial data were then split into train and test datasets at a ratio of 80:20. Scikit-learn's[131] (v1.3.0) *GridSearchCV()* was applied to select the best model hyperparameters for classifier training, and *RandomForestClassifier()* was trained with 2,000 decision trees, using parameters such as bootstrap sampling and square root feature selection at each split, achieving high (>0.96) out-of-bag, accuracy, precision, recall, and F1 scores. Trained classifier was then applied to the scRNA-seq dataset, predicting 4,318 tendon cells out of 108,617 total. Predictions were validated against spatial gene score-based annotations using the top 20 tendon-enriched genes (including *SCX*, *MKX*, *TNMD*, *ABI3BP*, *GAS2* etc), computed with *sc.tl.score_genes()* (Figures S2A and S2B).

Embryonic single-cell data were subset to the random forest classifier predictions. Only samples with at least 20 cells in each of the batch effect categories ('samplename', 'kit', 'seq_protocol', 'sex') were retained. Samples sequenced with 5′ v1 kit as well as those sequenced using NovaSeq 6000 were removed due to significant batch effect confounding. Finally, genes detected in fewer than 5 cells were removed, leaving a total of 3,092 cells and 18,119 genes from 6 donors aged 6.5–9.3pcw. The data were re-normalized to log1pPF, and 4,000 highly variable genes were selected with flavor = "cell_ranger". The effects of the cell cycle scores were regressed out to remove those significant sources of uninteresting variation (sc.pp.regress_out(adata, ['S_score', 'G2M_score'])). The regressed counts were scaled, and the PCA was carried out with previously identified highly variable genes. The data were then clustered using Scanpy's Leiden algorithm at 0.3 resolution, identifying 7 gene expression clusters. Those were manually annotated through marker gene exploration using the results from the between-cluster Wilcoxon rank-sum test. Marker sets from CZ CellxGene's Cell Guide library for mesenchymal stem cells (CL:0000134) and chondrocytes (CL:0000138)[144,164] were used for gene scoring to aid annotation.

Embryonic tendon RNA velocity analysis

scVelo (v0.2.5) preprocessing functions were applied to the combined, spliced, and unspliced counts matrices to re-normalize and filter them using the default settings. The neighbors and moments were calculated using regressed PCA embeddings. RNA Velocity analysis was carried out using a dynamical gene expression model by recovering dynamics, calculating velocities, and constructing a velocity graph with default settings. Differential kinetics test was then run using the top 100 dynamical genes, and the velocity was recalculated with those in mind.

Embryonic and fetal tendon trajectory and fate analysis

Embryonic and fetal tendon sample counts matrices were reorganised by sample age in ascending order. Harmony[145] (v0.1.4) function *harmony.core.augmented_affinity_matrix()* was used with top 40 PC loadings (containing over 90% of total variation) and 20 k-nearest neighbors (knn) to construct an augmented affinity matrix that incorporated the developmental age information into the similarity measures between cells. Force-directed layouts based on this matrix are shown in Figure 4A. This matrix was then used to construct diffusion maps with 20 knn using Palantir[88] (v1.3.1). The diffusion map embeddings were used to determine the multi-scale space of the data through 9 eigenvectors identified at the first eigengap. These multiscale space embeddings served as basis for tSNE dimensionality reduction, recalculation of the nearest neighbors (knn = 15), and creation of the force-directed graphs shown in Figure 4C.

Palantir trajectory analysis was run using multiscale space embeddings (knn = 20, num_waypoints = 2000), with differentiation initiation and termination cells selected based on the most extreme values in multiscale diffusion components. CellRank[89] (v1.5.1) Palantir pseudotime kernel was initiated, and the transition matrices were computed using a Generalized Perron Cluster Cluster Analysis (GPCCA) estimator with default settings. By analysing real eigenvalue plots, 3 distinct, stable macrostates representing terminal states were identified. Absorption probabilities were then computed using default settings, calculating lineage drivers for each terminal state. A single initial state was determined using a backward kernel based on the same process. Log1pPF-normalized, non-imputed counts were used to calculate correlations between lineage drivers shown in Figure 4F.

To bridge the gap between initial and terminal states, scVelo's *scvelo.tl.recover_latent_time()* was used to recover latent time, and *scvelo.tl.paga(adata, groups="cell_type", root_key="initial_states_probabilities", end_key="terminal_-states_probabilities", use_time_prior="palantir_pseudotime")* was applied to calculate a directed PAGA incorporating Palantir pseudotime as prior. This was used as basis for plotting cell fate probabilities shown in Figures 4D and 4E.

Embryonic and fetal data integration and trajectory inference

Embryonic scRNA-seq and fetal snRNA-seq datasets were integrated using scGen[92] (v2.1.1), with different sequencing runs specified as batches. The model was trained until convergence using default settings for a total of 28 epochs. Corrected latent space embeddings were used for the generation of a Harmony augmented affinity matrix. This was followed by Palantir multiscale diffusion map calculation and force-directed graph plotting, as described earlier. Following the generation of PAGA graphs, the Immune Cells were removed from further trajectory analysis due to them being disconnected from the rest of the cells even at low thresholds.

To reconstruct developmental trajectories, moscot[93] (v0.3.3) was applied. The genes were re-filtered to retain only those with more than 20 counts. Next, a TemporalProblem object was initiated with scGen-corrected latent embeddings, and the proliferation and apoptosis scores were obtained. The temporal problem was solved using manually optimised parameters (epsilon = 1e-3, tau_a = 0.99, tau_b = 0.999, scale_cost = "mean", batch_size = 1200). Cell transition scores were calculated to determine putative cell ancestors and descendants.

CellRank[91] (v2.0.2) RealTimeKernel was initiated from the TemporalProblem object, and the transition matrices were computed with self_transitions = "all", conn_weight = 0.2, and threshold = "auto_local". As before, GPCCA estimator was used. Overall, 7 macrostates were identified at the largest eigengap, out of which 4 corresponded to the major fetal tendon fibroblast cell types and were manually set to be terminal. The initial states were set manually to the putative tendon fibroblast precursor populations identified at 7.2 and 8.4pcw.

Embryonic, fetal and adult data integration

Processed, filtered, and annotated adult Achilles tendon (*N* = 6 samples, split by microanatomical regions; Table S1) and quadriceps tendon (*N* = 7 samples, healthy or torn midbodies; Table S1) datasets provided by the Tendon Seed Network[135,162] were concatenated with processed and annotated embryonic and fetal tendon datasets. Cells with fewer than 200 genes and genes in fewer than 30 counts were filtered out, yielding 176,691 cells and 32,869 genes. Top 7,000 highly variable genes were selected in a batch-aware manner using *scanpy.pp.highly_variable-_genes(batch_key="sampletype")*. Data integration was performed using scVI modeling on unnormalized, highly variable counts, with library preparation batches ("libbatch"), "sampletype", and the cell cycle phase scores ("G2M_score" and "S_score") specified as main batch effects. The model's hyperparameters were optimised with scVI's autotune to 'n_hidden': 256, 'n_latent': 50, 'n_layers': 1, 'dropout_rate': 0.1, 'gene_likelihood': 'zinb', and 'dispersion': 'gene-batch'. The model was trained for 80 epochs until training and validation set convergence. Next, cell type labels were harmonised using the scANVI model. The resulting embeddings were used for the computation of neighborhood graphs and dimensionality reduction with UMAP.

The data were normalized to log1pPF and subsequently subjected to Wilcoxon rank-sum test differential gene expression analysis (*scanpy.tl.rank_genes_groups()*), which was performed separately for fetal and adult cell populations. For each specified cell type within fetal tendons, the top 50 differentially expressed genes were extracted. Marker gene overlap analysis using *scanpy.tl.marker_-gene_overlap()* was conducted to quantify and visualize transcriptional similarities between the fetal top 50 DEGs and the marker genes derived from the adult differential expression analysis. Normalisation of overlap scores was performed relative to the fetal DEGs, providing a proportion of fetal marker genes detected in each adult cell type (Figure S3B).

To achieve more robust cell type label harmonisation, CellHint[165] (v1.0.0) was applied to the different tendon types across the embryonic, fetal, and adult age groups. Specifically, *cellhint.harmonize()* function was used to calculate euclidean distances between cells within batch-corrected scVI embeddings, constructing a harmonisation graph of cell type annotations. The datasets were specified to be harmonised sequentially, from embryonic to adult stages.

Gene regulatory network analysis

Single-cell regulatory network inference and clustering (SCENIC) analysis was performed for embryonic, fetal, and adult quadriceps tendon datasets using pyscenic[139] (v0.12.1). Gene regulatory networks were inferred using the GRNBoost2 algorithm with default settings. The input data were raw, unnormalized counts. Human transcription factors were predefined using the 'allTFs_hg38.txt' list from the Aertslab GitHub repository. The resulting TF-gene interactions were utilised to infer co-expression modules, identify enriched motifs, and predict regulons with the *pyscenic ctx* command. Genome rankings (.feather) and motif annotation (.tbl) v10 files

were obtained from the Aertslab cistarget resources webpage. A total of 335 embryonic regulons, 285 regulons from 12 pcw fetal samples, 237 regulons from 17 pcw fetal samples, 286 regulons from 20 pcw fetal samples, and 193 adult regulons were identified.

Regulon activity within individual cells was quantified using the *pyscenic aucell* command, with an AUC threshold of 0.1 applied to the embryonic dataset and the default threshold of 0.05 applied to the fetal and adult datasets. Binarization of regulon activity was achieved using a Gaussian mixture model, resulting in matrices indicating active and inactive regulons per cell.

The binarized regulon matrices underwent several transformations to extract and normalize regulon activation patterns across different cell populations. Initially, cells were grouped according to their cell type annotations, allowing the aggregation of active regulons by calculating the sum of active cells per regulon within each group. Subsequently, these summaries were normalized based on the total cell count per group to account for variations in cell numbers. Next, normalized regulon activation frequencies within each group were further *Z* score normalized, facilitating comparative analysis across developmental stages and conditions. The z-scored regulon activity patterns were visualized using custom clustermaps. Individual regulons were explored and summarized using the STRING database (*stringdb,*[137] v12.0). Large regulons were subjected to pathway analysis with gProfiler g:GOSt[159] as previously described.

QUANTIFICATION AND STATISTICAL ANALYSIS

Quantification of fetal cell type compositional changes

Cell type compositional changes between 12 and 20 pcw fetal tendons were assessed using the scCODA[166] (v0.1.9) package. A MuData object was generated from the fetal tendon snRNA-seq dataset, and scCODA was run with modality_key = 'coda'. The cluster of nervous system-associated cells was automatically selected as the reference. The model was trained using 11,000 NUTS (No-U-Turn Sampler) iterations, and statistically credible shifts in cell type proportions were identified. Results are summarized in Figure 1E.

Quantifying metrics from tendon histology

QuPath[160] (v0.4.3) classification functions were used to manually delineate tendon regions within H&E-stained tissue samples. From these regions, 3–6 random 250 × 250 μm tiles were extracted and processed using ImageJ (v1.53t). First, hematoxylin and eosin channels were separated using color deconvolution. Subsequently, the hematoxylin channel was used for nuclei segmentation using the StarDist[146] ImageJ plugin (v0.3.0) (see Figure S8).

Following this segmentation, quantitative analyses were performed to determine the number of nuclei, the mean area of the nuclei, and the maximum distance to the centroid of each nucleus (Figure S7). Statistical analysis was performed using scipy[147] (v1.11.1), statsmodels[148] (v0.14.0), and scikit-posthocs[149] (v0.9.0). The normality of the data distributions for the number of nuclei, their areas, and maximum distances to their centers was assessed using the Shapiro-Wilk test as well as visual inspections of histograms and Q-Q plots. For data that approximated normal distributions – the nucleus area and maximum distance to nucleus centroid variables – analysis of variance (ANOVA) was performed to test for significant differences across different age groups. For the number of nuclei, which exhibited an almost bimodal distribution, the Kruskal-Wallis H-test was used to assess significant differences across age groups. Post-hoc analysis was conducted to identify specific group differences. Tukey's Honest Significant Difference test was used following the ANOVA, and Dunn's test with Bonferroni correction was employed after the Kruskal-Wallis H-test.

Fetal tendon pseudobulk differential gene expression analysis

For differential gene expression (DGE) analysis of fetal cell types across different ages, the raw single-nuclei sequencing counts were converted into "pseudobulk" expression profiles using custom functions inspired by Heumos et al.[143] Briefly, the data were partitioned by distinct cell types, and a donor-filtering criterion was applied to retain only tissue donors with a minimum cell count threshold of 30. Three pseudoreplicates were then generated by randomly subsampling cells within retained donors, and these pseudoreplicates were subsequently transformed into pseudobulk samples by aggregating expression counts. Metadata associated with donors, experimental conditions, and cell identity were preserved.

DGE analysis was carried out individually for each of the cell type pseudobulked counts using DESeq2[150] (v1.40.2). Both LRT and Wald tests were done to assess the differences between different developmental timepoints and tissue types. LRT results with p-adjusted values of <0.01 were clustered to investigate common patterns in gene expression changes over time. The resulting cluster gene lists were subjected to pathway analysis against all expressed genes using the gProfiler g:GOSt tool[159] with Benjamini-Hochberg FDR set to <0.05 and pathway term sizes restricted to between 20 and 500 terms (Table S2). Shared or cell type-specific terms for each cluster were then isolated and manually grouped into common functional categories to reduce data dimensionality and enable comparisons.

Fetal and adult tendon pseudobulk differential gene expression analysis

For DGE analysis across different fetal and adult cell types, the raw single-nuclei sequencing counts were converted into grouped "donor + tissue type + cell type" pseudobulk expression profiles using custom functions inspired by Heumos et al.[143] Differential gene expression analysis was carried out separately for the specified groups containing specific CellHint-paired fetal and adult cell types using DESeq2[150] (v1.40.2). Wald tests were used to identify all genes that were significantly up- or downregulated in adult

cell types compared to the fetal cell types (with *p-adjusted* < 0.01, log_2FC > ±1). These genes were used for pathway analysis against all expressed genes using the gProfiler g:GOSt tool[159] with Benjamini-Hochberg FDR set to <0.01 and term sizes restricted to between 20 and 500 terms (Table S3). The identified up- or downregulated GO:BP terms for each CellHint-aligned pair of fetal and adult fibroblasts were compared against each other using Venn diagrams made with Venny[130] (v2.1.0). The pair-specific pathways were then manually summarized by common functions into broad categories to enable comparisons (Figures S12, S13, S14, and S15).

Cell Reports, Volume *45*

Supplemental information

Cellular and molecular landscapes of human tendons across the lifespan revealed by spatial and single-cell transcriptomics

Alina Kurjan, Jolet Y. Mimpen, Lorenzo Ramos-Mucci, Ali C. Aksu, Carla J. Cohen, Mate Naszai, Christopher D. Buckley, Adam P. Cribbs, Mathew J. Baldwin, and Sarah J.B. Snelling

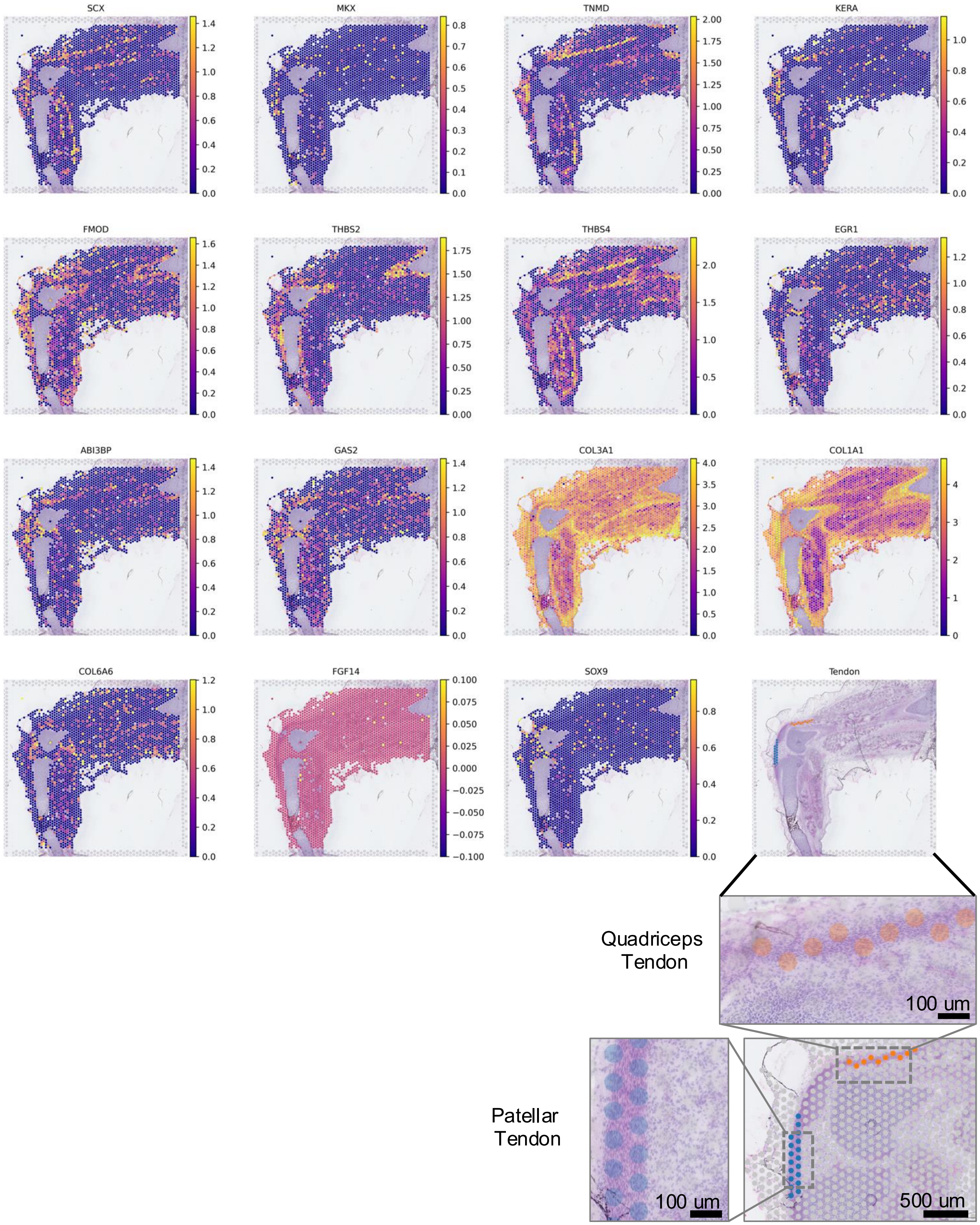

SCX
MKX
TNMD
KERA
FMOD
THBS2
THBS4
EGR1
ABI3BP
GAS2
COL3A1
COL1A1
COL6A6
FGF14
SOX9
Tendon
Quadriceps Tendon
100 um
Patellar Tendon
100 um
500 um

Figure S1. Tendon cell marker expression and tissue demarcation in a whole 8 post-conception week embryonic human leg.

Visium scatterplots show normalised gene expression values for key tendon cell markers, revealing their spatial distribution within the developing leg. The 'Tendon' panel shows manually annotated tendon regions identified from H&E-stained tissue sections. These annotated regions were used to train the random forest classifier, enabling the identification of tendon cell types in embryonic whole limb single-cell data based on this spatial RNA-sequencing data. The inset shows the annotated knee region of the embryonic limb. Orange spots correspond to the quadriceps tendon, while blue spots demarcate the patellar tendon.

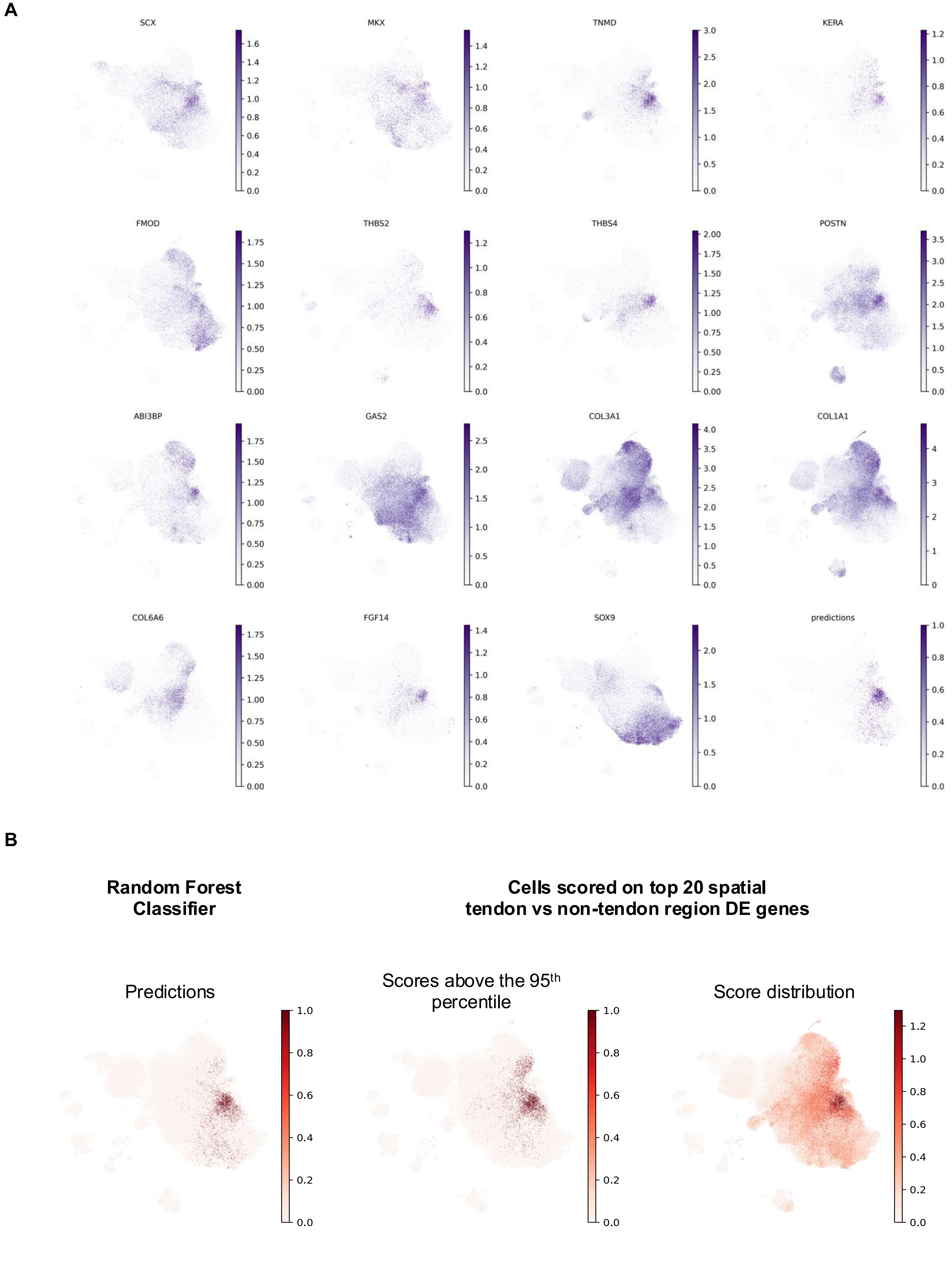
A
SCX
MKX
TNMD
KERA
FMOD
THBS2
THBS4
POSTN
ABI3BP
GAS2
COL3A1
COL1A1
COL6A6
FGF14
SOX9
predictions
B
Random Forest Classifier
Cells scored on top 20 spatial tendon vs non-tendon region DE genes
Predictions
Scores above the 95th percentile
Score distribution

Figure S2. Expression of early tendon markers in human embryonic single-cell RNA-sequencing data from fore- and hindlimbs of 11 human embryonic donors.

(A) UMAPs show normalised gene expression values for early tendon markers relevant to early tendon development. The ‘predictions’ panel shows the likely tendon cell types identified using a random forest classifier, which was trained on spatial RNA-sequencing data from 8 post-conception week embryonic hindlimbs. (B) UMAPs show comparison of random forest classifier vs gene scoring methods for tendon cell identification within whole limb embryonic scRNA-seq data. Random forest classifier was trained on the spatial gene expression profiles of tendon and non-tendon regions. The Predictions UMAP plot (left) highlights all cells identified as tendons by this classifier. Middle and right UMAP plots show tendon cell identification by cell scoring on the top 20 differentially expressed genes between spatial tendon and non-tendon regions. Right plot shows the scores. Middle plot shows the cells with scores above the 95th percentile.

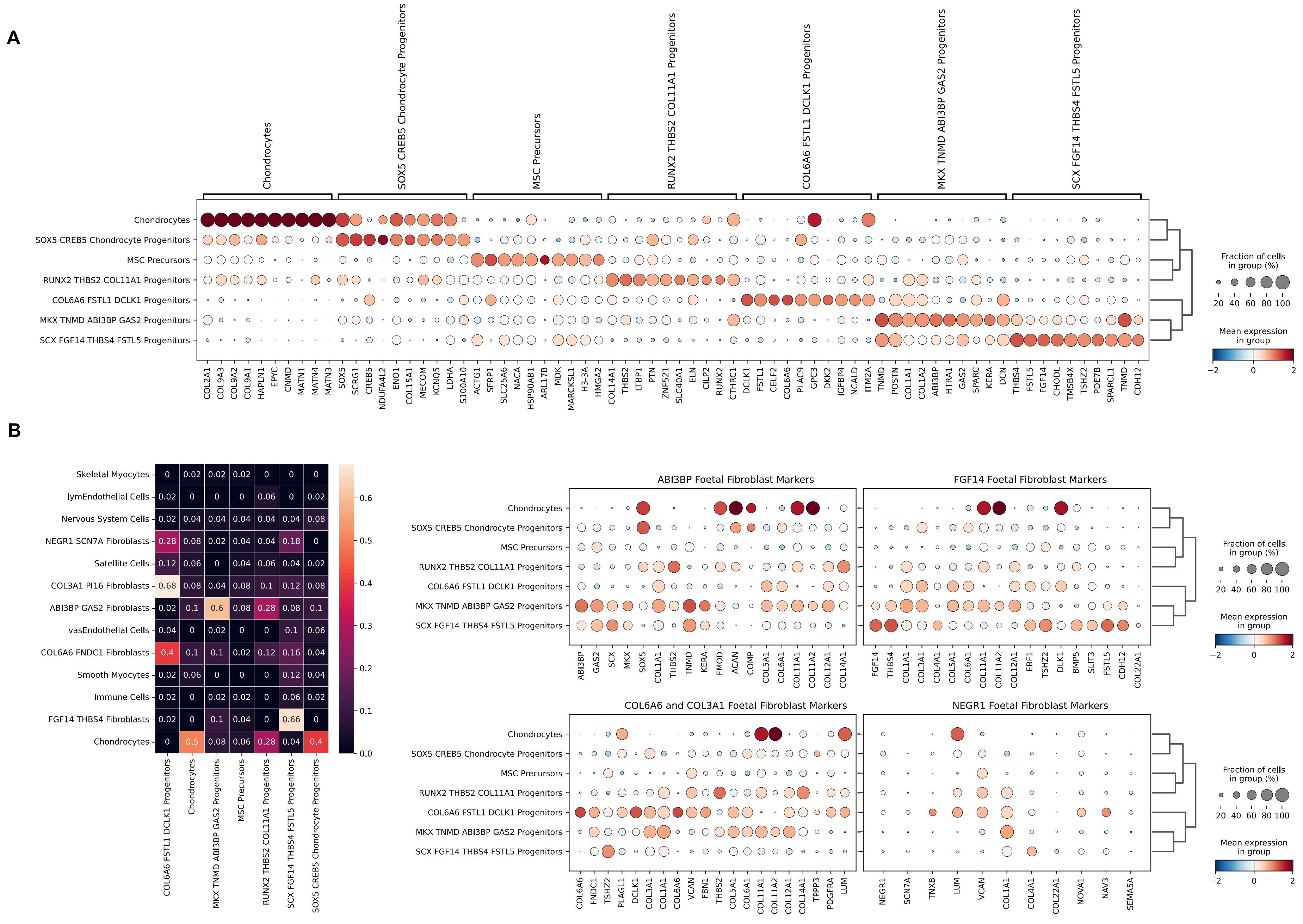
A
Chondrocytes
SOX5 CREB5 Chondrocyte Progenitors
MSC Precursors
RUNX2 THBS2 COL11A1 Progenitors
COL6A6 FSTL1 DCLK1 Progenitors
MKX TNMD ABI3BP GAS2 Progenitors
SCX FGF14 THBS4 FSTL5 Progenitors
COL2A1 COL9A3 COL9A2 COL9A1 HAPLN1 EPYC CNMD MATN1 MATN4 MATN3 SOX5 SCRG1 CREB5 NDUFA4L2 ENO1 COL15A1 MECOM KCNQ5 LDHA S100A10 ACTG1 SFRP1 SLC25A6 NACA HSP90AB1 ARL17B MDK MARCKSL1 H3-3A HMGA2 COL14A1 THBS2 LTBP1 PTN ZNF521 SLC40A1 ELN CILP2 RUNX2 CTHRC1 DCLK1 FSTL1 CELF2 COL6A6 PLAC9 GPC3 DKK2 IGFBP4 NCALD ITM2A TNMD POSTN COL1A1 COL1A2 ABI3BP HTRA1 GAS2 SPARC KERA DCN THBS4 FSTL5 FGF14 CHODL TMSB4X TSHZ2 PDE7B SPARCL1 TNMD CDH12
Fraction of cells in group (%)
20 40 60 80 100
Mean expression in group
−2 0 2
B
Skeletal Myocytes
lymEndothelial Cells
Nervous System Cells
NEGR1 SCN7A Fibroblasts
Satellite Cells
COL3A1 PI16 Fibroblasts
ABI3BP GAS2 Fibroblasts
vasEndothelial Cells
COL6A6 FNDC1 Fibroblasts
Smooth Myocytes
Immune Cells
FGF14 THBS4 Fibroblasts
Chondrocytes
COL6A6 FSTL1 DCLK1 Progenitors
Chondrocytes
MKX TNMD ABI3BP GAS2 Progenitors
MSC Precursors
RUNX2 THBS2 COL11A1 Progenitors
SCX FGF14 THBS4 FSTL5 Progenitors
SOX5 CREB5 Chondrocyte Progenitors
0.0 0.1 0.2 0.3 0.4 0.5 0.6
ABI3BP Foetal Fibroblast Markers
ABI3BP GAS2 SCX MKX SOX5 COL1A1 THBS2 TNMD KERA FMOD ACAN COMP COL5A1 COL6A1 COL11A1 COL11A2 COL12A1 COL14A1
FGF14 Foetal Fibroblast Markers
FGF14 THBS4 COL1A1 COL3A1 COL4A1 COL5A1 COL6A1 COL11A1 COL11A2 COL12A1 EBF1 TSHZ2 DLK1 BMP5 SLIT3 FSTL5 CDH12 COL22A1
COL6A6 and COL3A1 Foetal Fibroblast Markers
COL6A6 FNDC1 TSHZ2 PLAGL1 DCLK1 COL3A1 COL1A1 COL6A6 VCAN FBN1 THBS2 COL5A1 COL6A1 COL11A1 COL11A2 COL12A1 COL14A1 TPPP3 PDGFRA LUM
NEGR1 Foetal Fibroblast Markers
NEGR1 SCN7A TNXB LUM VCAN COL1A1 COL4A1 COL22A1 NOVA1 NAV3 SEMA5A
Fraction of cells in group (%)
20 40 60 80 100
Mean expression in group
−2 0 2

Figure S3. Differentially expressed genes in human 6.5-9.3 post-conception week (pcw) embryonic tendon cell types and comparison with foetal tendon cell signatures.

(A) Dotplot shows clustered log1pPF-normalised and scaled expression of top 10 differentially expressed genes for each embryonic tendon cell type. (B) (Left) Matrixplot shows proportional overlap of the top 50 differentially expressed genes identified in cell types from 12 pcw foetal tendons against an array of embryonic cell types. Each cell in the matrix represents the percentage of overlapping DEGs, calculated as the number of shared genes divided by the total number of genes in the embryonic reference set, multiplied by 100 to express the value as a percentage. (Right) Dotplots show clustered log1pPF-normalised and scaled expression of foetal tendon fibroblast markers in embryonic cell types.

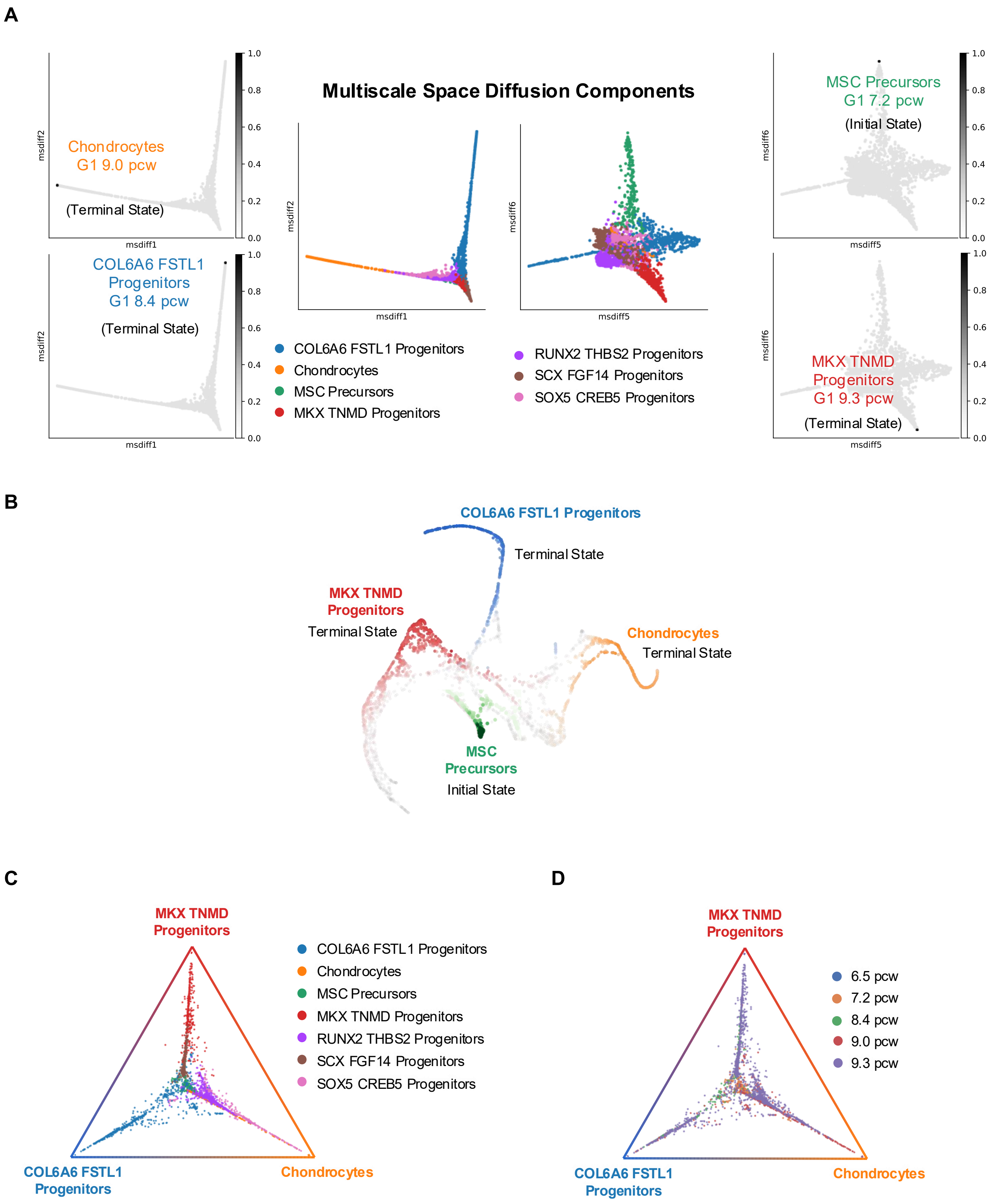

A
Multiscale Space Diffusion Components
Chondrocytes
G1 9.0 pcw
(Terminal State)
COL6A6 FSTL1
Progenitors
G1 8.4 pcw
(Terminal State)
MSC Precursors
G1 7.2 pcw
(Initial State)
MKX TNMD
Progenitors
G1 9.3 pcw
(Terminal State)
msdiff1
msdiff2
msdiff5
msdiff6
1.0
0.8
0.6
0.4
0.2
0.0
COL6A6 FSTL1 Progenitors
Chondrocytes
MSC Precursors
MKX TNMD Progenitors
RUNX2 THBS2 Progenitors
SCX FGF14 Progenitors
SOX5 CREB5 Progenitors
B
COL6A6 FSTL1 Progenitors
Terminal State
MKX TNMD
Progenitors
Terminal State
Chondrocytes
Terminal State
MSC
Precursors
Initial State
C
MKX TNMD
Progenitors
COL6A6 FSTL1
Progenitors
Chondrocytes
COL6A6 FSTL1 Progenitors
Chondrocytes
MSC Precursors
MKX TNMD Progenitors
RUNX2 THBS2 Progenitors
SCX FGF14 Progenitors
SOX5 CREB5 Progenitors
D
MKX TNMD
Progenitors
COL6A6 FSTL1
Progenitors
Chondrocytes
6.5 pcw
7.2 pcw
8.4 pcw
9.0 pcw
9.3 pcw

Figure S4. CellRank fate mapping of embryonic tendon cell differentiation.

(A) Selected initial and terminal states using multiscale space embedding components for Palantir pseudotime calculation. Multiscale space was calculated based on the Palantir diffusion map embeddings. (B) Force-directed graph showing the location of manually selected cells corresponding to the initial and terminal states. (C,D) Circular projections of fate probabilities coloured by (C) cell states or (D) donor ages in weeks post-conception (pcw) showing the trajectory and density of cells transitioning from initial MSC Precursors in the middle towards the specified terminal fates.

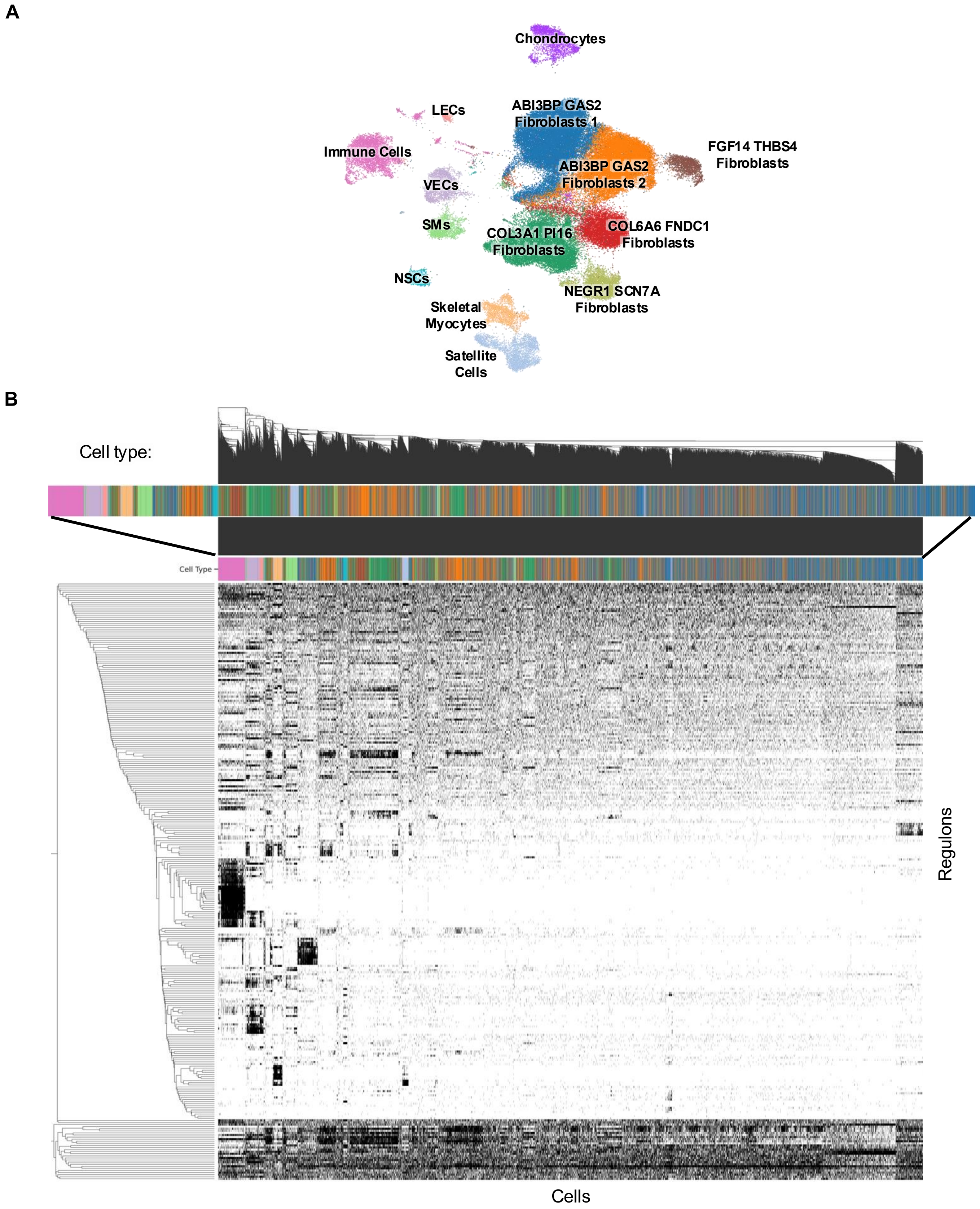
A
Chondrocytes
LECs
ABI3BP GAS2 Fibroblasts 1
Immune Cells
FGF14 THBS4 Fibroblasts
ABI3BP GAS2 Fibroblasts 2
VECs
SMs
COL3A1 PI16 Fibroblasts
COL6A6 FNDC1 Fibroblasts
NSCs
NEGR1 SCN7A Fibroblasts
Skeletal Myocytes
Satellite Cells
B
Cell type:
Cell Type
Regulons
Cells

Figure S5. SCENIC analysis of cell type-specific regulon activity.

(A) UMAP showing annotated 12-20pcw foetal Achilles and quadriceps tendon cell types. (B) Clustermap showing regulon-based clustering in 20 post-conception week (pcw) foetal Achilles and quadriceps tendon cell types. Each row represents a regulon, and each column corresponds to an individual cell. Cell types are indicated by colours in the top bar, synchronised with the UMAP visualisation.

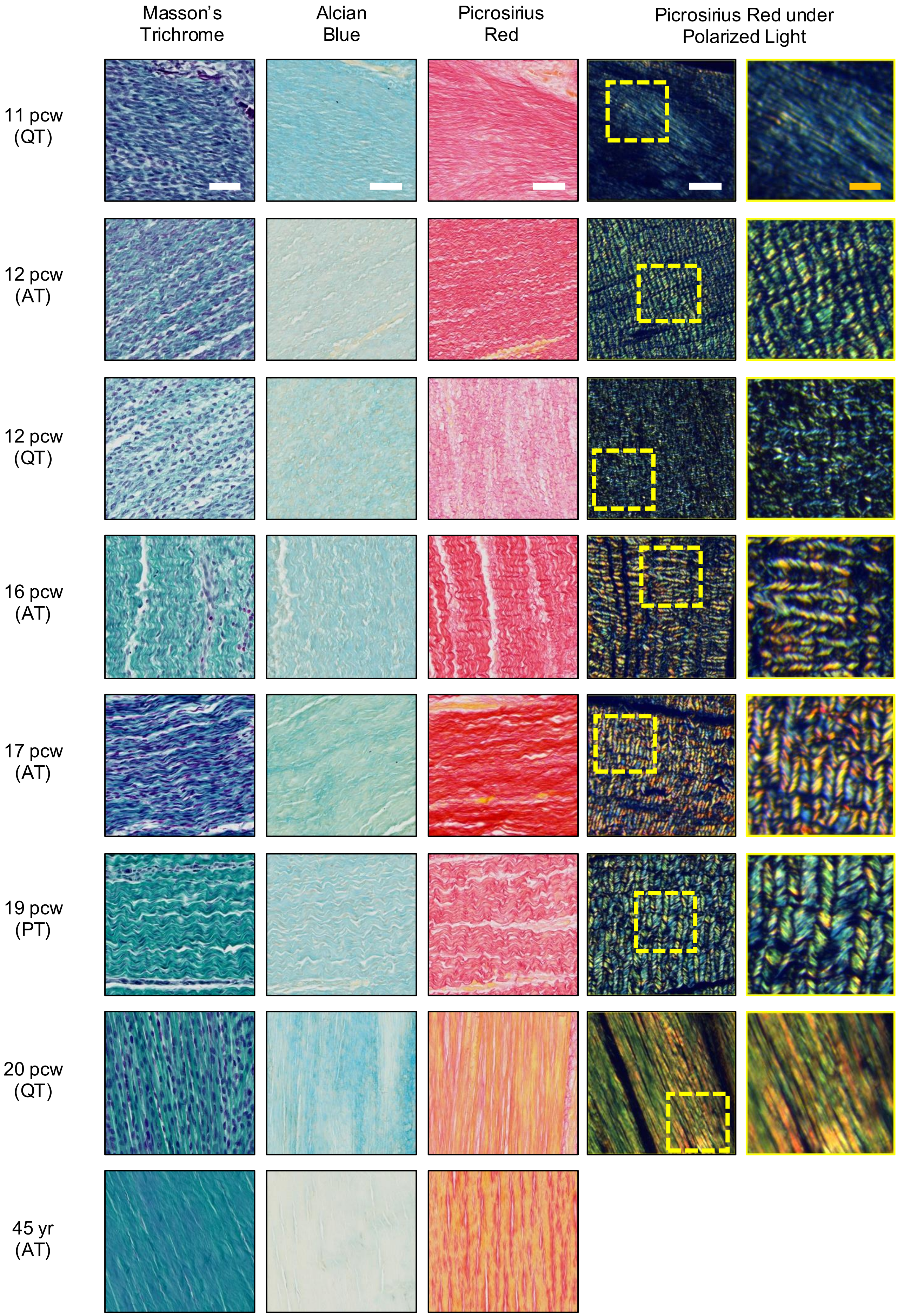

Masson's Trichrome
Alcian Blue
Picrosirius Red
Picrosirius Red under Polarized Light
11 pcw (QT)
12 pcw (AT)
12 pcw (QT)
16 pcw (AT)
17 pcw (AT)
19 pcw (PT)
20 pcw (QT)
45 yr (AT)

Figure S6. Photomicrographs of foetal and adult tendon tissues stained with masson's trichrome, alcian blue, and picrosirius red.

Brightfield microscopy was used for visualisation, with polarizing light filters additionally applied to the picrosirius red-stained tissues. Masson's trichrome stains for collagen fibres (blue), muscle fibres (red), and cell nuclei (dark purple). Alcian blue specifically stains for acidic mucopolysaccharides and glycosaminoglycans, which are components of proteoglycans and glycoproteins forming the ground substance (blue). Picrosirius red stains for collagen fibres, which appear red under brightfield microscopy and show varying colours (red, orange, yellow, and green) under polarized light, depending on the thickness and type of collagen. The size bars correspond to 50 μm (white) or 20 μm (orange). Examined tissues include Achilles tendon (AT), quadriceps tendon (QT), and patellar tendon (PT). Foetal tissues are denoted by post-conception weeks (pcw) and adult tissues by years (yr).

A

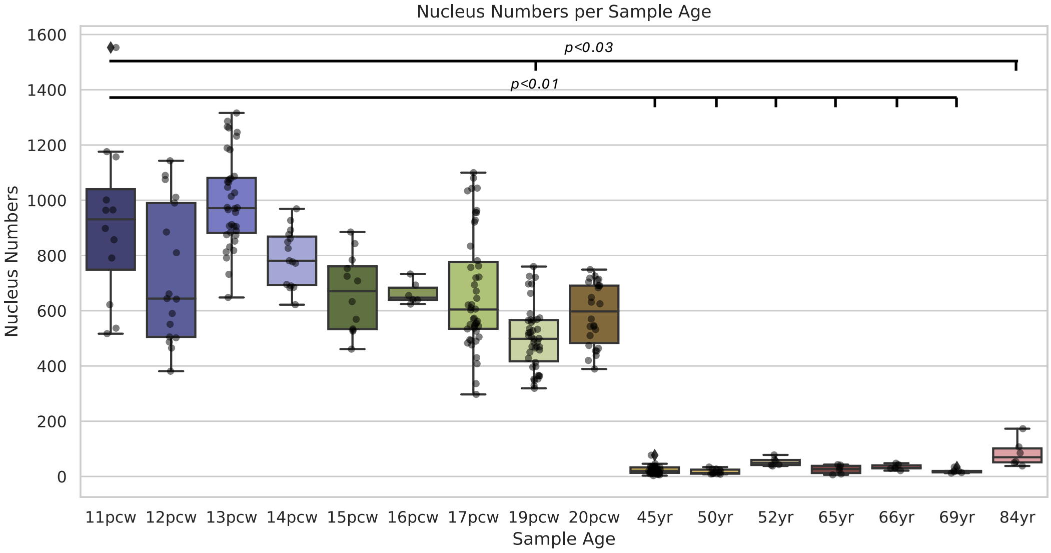
Nucleus Numbers per Sample Age
p<0.03
p<0.01
Nucleus Numbers
1600
1400
1200
1000
800
600
400
200
0
11pcw 12pcw 13pcw 14pcw 15pcw 16pcw 17pcw 19pcw 20pcw 45yr 50yr 52yr 65yr 66yr 69yr 84yr
Sample Age

B

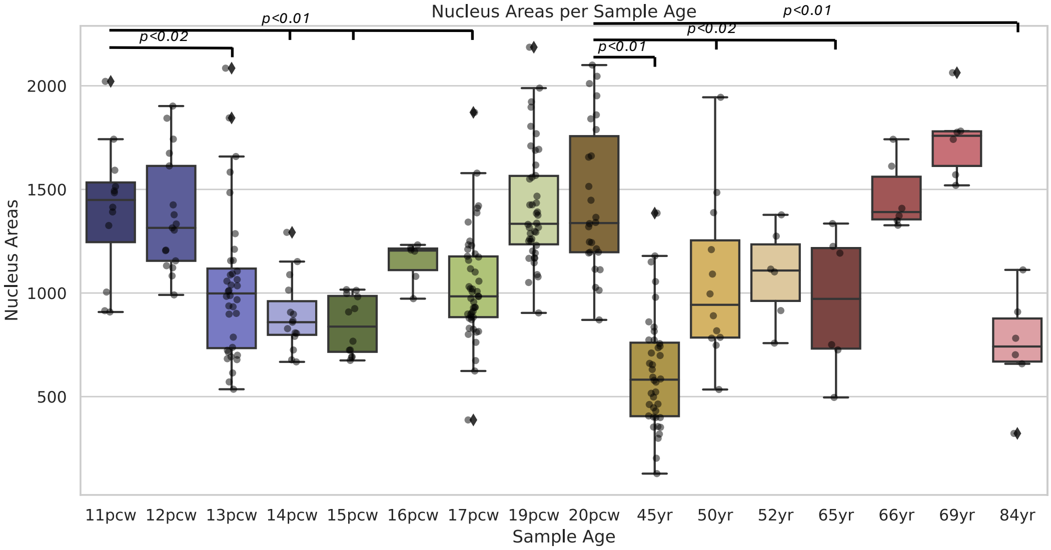
Nucleus Areas per Sample Age
p<0.01
p<0.02
p<0.01
p<0.02
p<0.01
Nucleus Areas
2000
1500
1000
500
11pcw 12pcw 13pcw 14pcw 15pcw 16pcw 17pcw 19pcw 20pcw 45yr 50yr 52yr 65yr 66yr 69yr 84yr
Sample Age

C

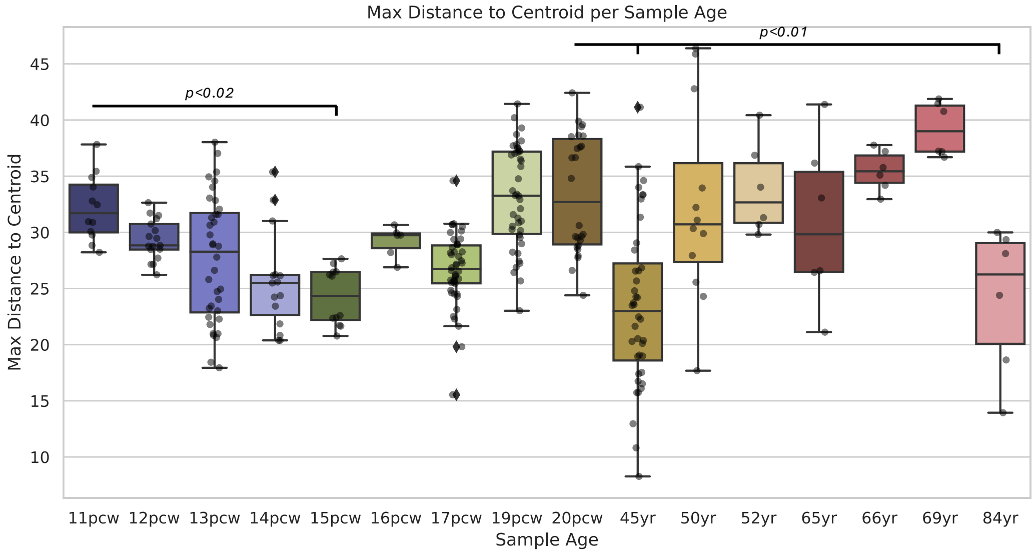
Max Distance to Centroid per Sample Age
p<0.01
p<0.02
Max Distance to Centroid
45
40
35
30
25
20
15
10
11pcw 12pcw 13pcw 14pcw 15pcw 16pcw 17pcw 19pcw 20pcw 45yr 50yr 52yr 65yr 66yr 69yr 84yr
Sample Age

Figure S7. Results of StarDist quantification of nuclear properties in haematoxylin-stained tissues.

Boxplots display (A) nuclei numbers, (B) nuclei areas, and (C) maximum distances to nuclei centroids for each sample age. Foetal tissues are denoted by post-conception weeks (pcw) and adult tissues by years (yr). Adult ages correspond to single donors. Statistical analysis was performed using a Kruskal-Wallis test followed by Dunn's post-hoc test for nuclei numbers (A), and ANOVA with Tukey HSD for nuclei areas and distances (B, C), measured in pixels within 250 μm^2 tiles. Significant comparisons against a single timepoint are shown: all foetal and adult comparisons against 11 pcw in (A), and foetal comparisons against 11 pcw and adult comparisons against 20 pcw in (B) and (C).

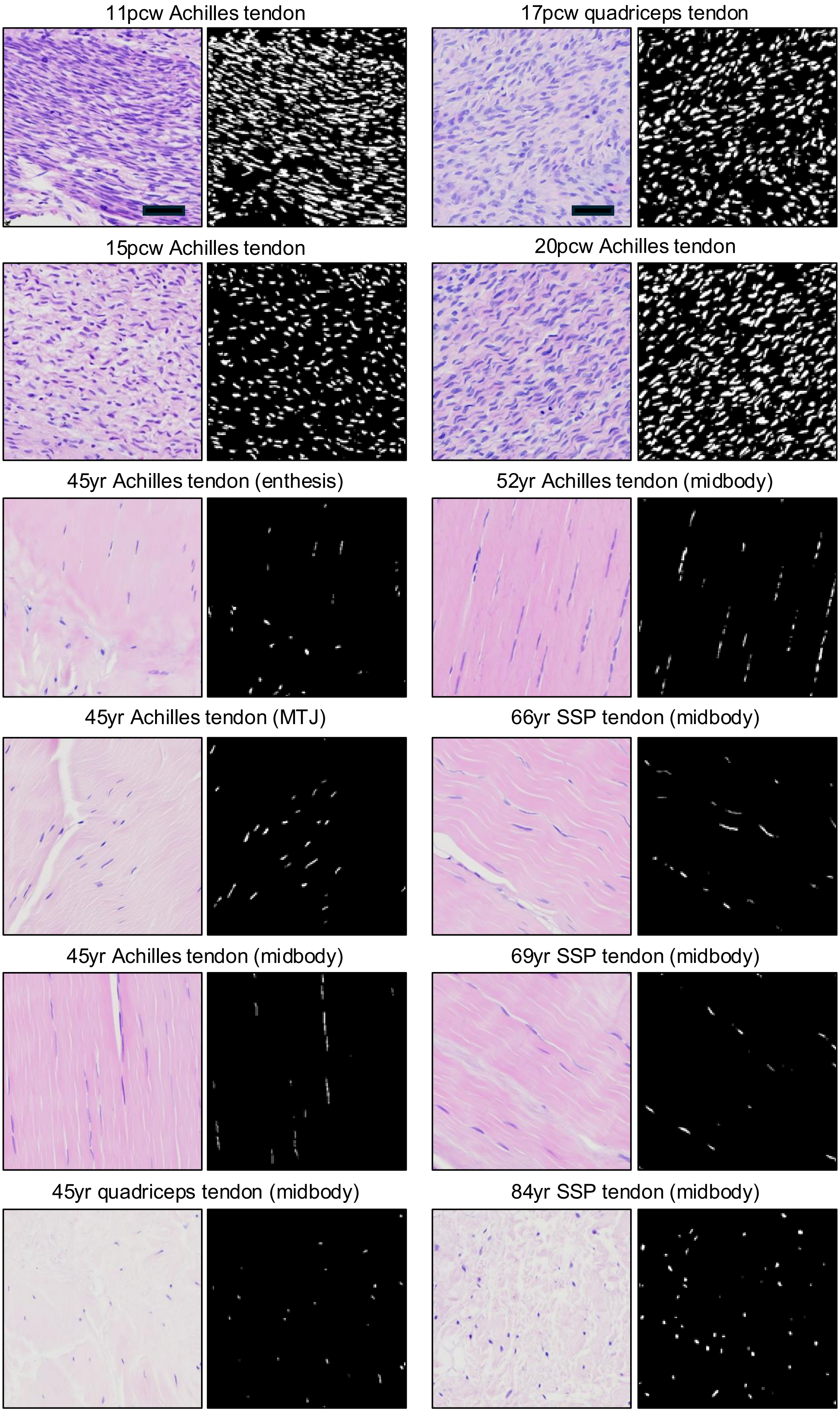
11pcw Achilles tendon
17pcw quadriceps tendon
15pcw Achilles tendon
20pcw Achilles tendon
45yr Achilles tendon (enthesis)
52yr Achilles tendon (midbody)
45yr Achilles tendon (MTJ)
66yr SSP tendon (midbody)
45yr Achilles tendon (midbody)
69yr SSP tendon (midbody)
45yr quadriceps tendon (midbody)
84yr SSP tendon (midbody)

Figure S8. Examples of foetal and adult tendon sample tiles used for StarDist nuclei segmentation.

Left panels show the original H&E-stained images, while right panels display the corresponding isolated haematoxylin channels used for nuclei segmentation and measurements with StarDist. Size bars correspond to 50 µm. Foetal tissues are denoted by post-conception weeks (pcw) and adult tissues by years (yr). SSP: supraspinatus tendon of the shoulder; MTJ: myotendinous junction.

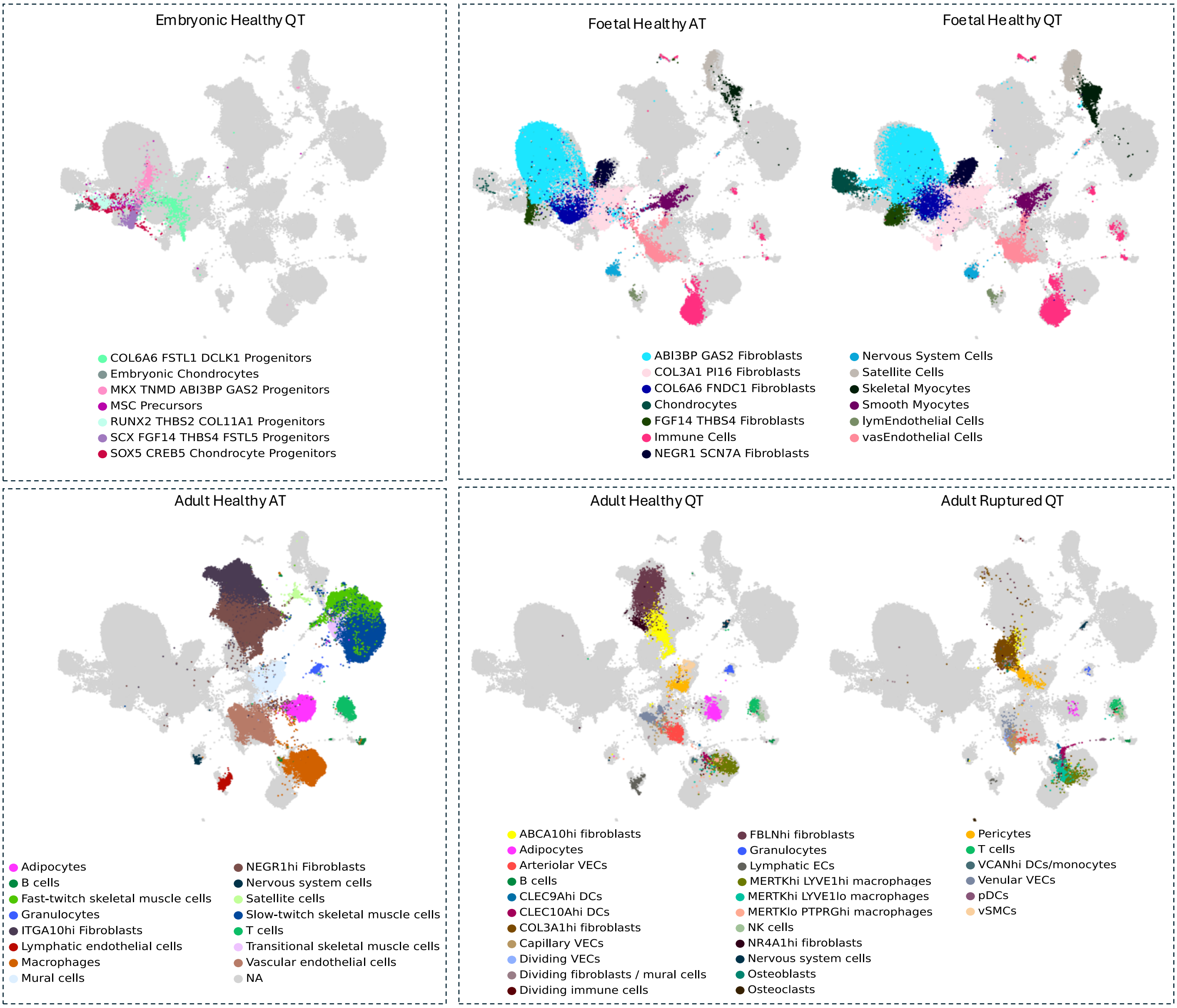
Embryonic Healthy QT
COL6A6 FSTL1 DCLK1 Progenitors
Embryonic Chondrocytes
MKX TNMD ABI3BP GAS2 Progenitors
MSC Precursors
RUNX2 THBS2 COL11A1 Progenitors
SCX FGF14 THBS4 FSTL5 Progenitors
SOX5 CREB5 Chondrocyte Progenitors
Foetal Healthy AT
Foetal Healthy QT
ABI3BP GAS2 Fibroblasts
COL3A1 PI16 Fibroblasts
COL6A6 FNDC1 Fibroblasts
Chondrocytes
FGF14 THBS4 Fibroblasts
Immune Cells
NEGR1 SCN7A Fibroblasts
Nervous System Cells
Satellite Cells
Skeletal Myocytes
Smooth Myocytes
lymEndothelial Cells
vasEndothelial Cells
Adult Healthy AT
Adipocytes
B cells
Fast-twitch skeletal muscle cells
Granulocytes
ITGA10hi Fibroblasts
Lymphatic endothelial cells
Macrophages
Mural cells
NEGR1hi Fibroblasts
Nervous system cells
Satellite cells
Slow-twitch skeletal muscle cells
T cells
Transitional skeletal muscle cells
Vascular endothelial cells
NA
Adult Healthy QT
Adult Ruptured QT
ABCA10hi fibroblasts
Adipocytes
Arteriolar VECs
B cells
CLEC9Ahi DCs
CLEC10Ahi DCs
COL3A1hi fibroblasts
Capillary VECs
Dividing VECs
Dividing fibroblasts / mural cells
Dividing immune cells
FBLNhi fibroblasts
Granulocytes
Lymphatic ECs
MERTKhi LYVE1hi macrophages
MERTKhi LYVE1lo macrophages
MERTKlo PTPRGhi macrophages
NK cells
NR4A1hi fibroblasts
Nervous system cells
Osteoblasts
Osteoclasts
Pericytes
T cells
VCANhi DCs/monocytes
Venular VECs
pDCs
vSMCs

Figure S9. Distribution of original cell type annotations from scANVI-integrated embryonic, foetal, and adult tendon datasets.

Annotated adult datasets were provided by Dr Carla Cohen (Achilles tendon) and Dr Jolet Mimpen (quadriceps tendon).

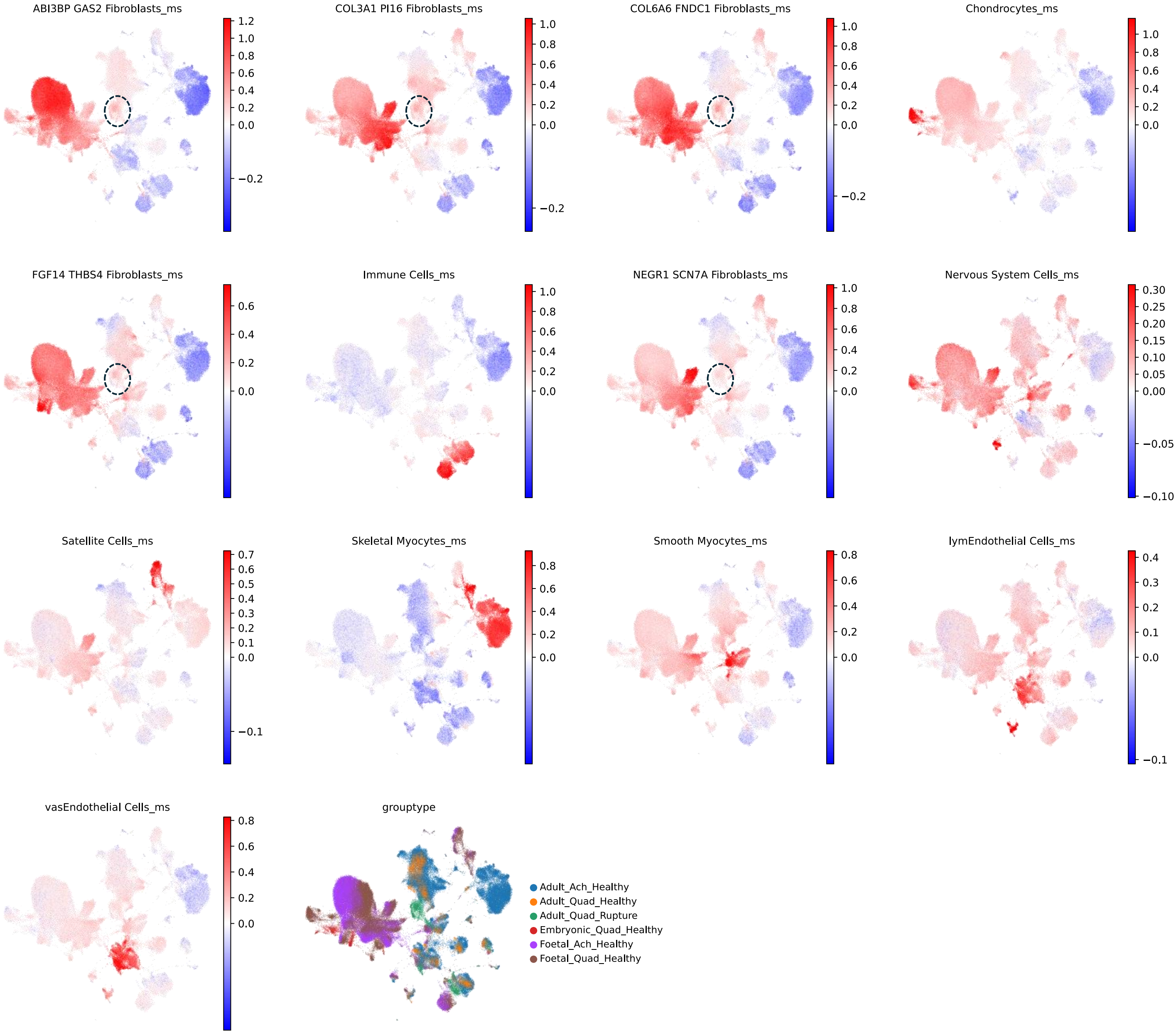
ABI3BP GAS2 Fibroblasts_ms
COL3A1 PI16 Fibroblasts_ms
COL6A6 FNDC1 Fibroblasts_ms
Chondrocytes_ms
FGF14 THBS4 Fibroblasts_ms
Immune Cells_ms
NEGR1 SCN7A Fibroblasts_ms
Nervous System Cells_ms
Satellite Cells_ms
Skeletal Myocytes_ms
Smooth Myocytes_ms
lymEndothelial Cells_ms
vasEndothelial Cells_ms
grouptype
Adult_Ach_Healthy
Adult_Quad_Healthy
Adult_Quad_Rupture
Embryonic_Quad_Healthy
Foetal_Ach_Healthy
Foetal_Quad_Healthy

Figure S10. Marker gene overlap between foetal and adult tendon cell types.

UMAPs show the spatial distribution of gene expression scores for the same top 50 DEGs specific to individual foetal tendon cell types (see Figure 6A). Colour intensity is scaled to the 99th percentile of the score distribution and indicates the relative expression level of the markers within the cells, with red corresponding to high expression of the top 50 foetal DEGs. Black ovals highlight the rupture-specific fibroblast population.

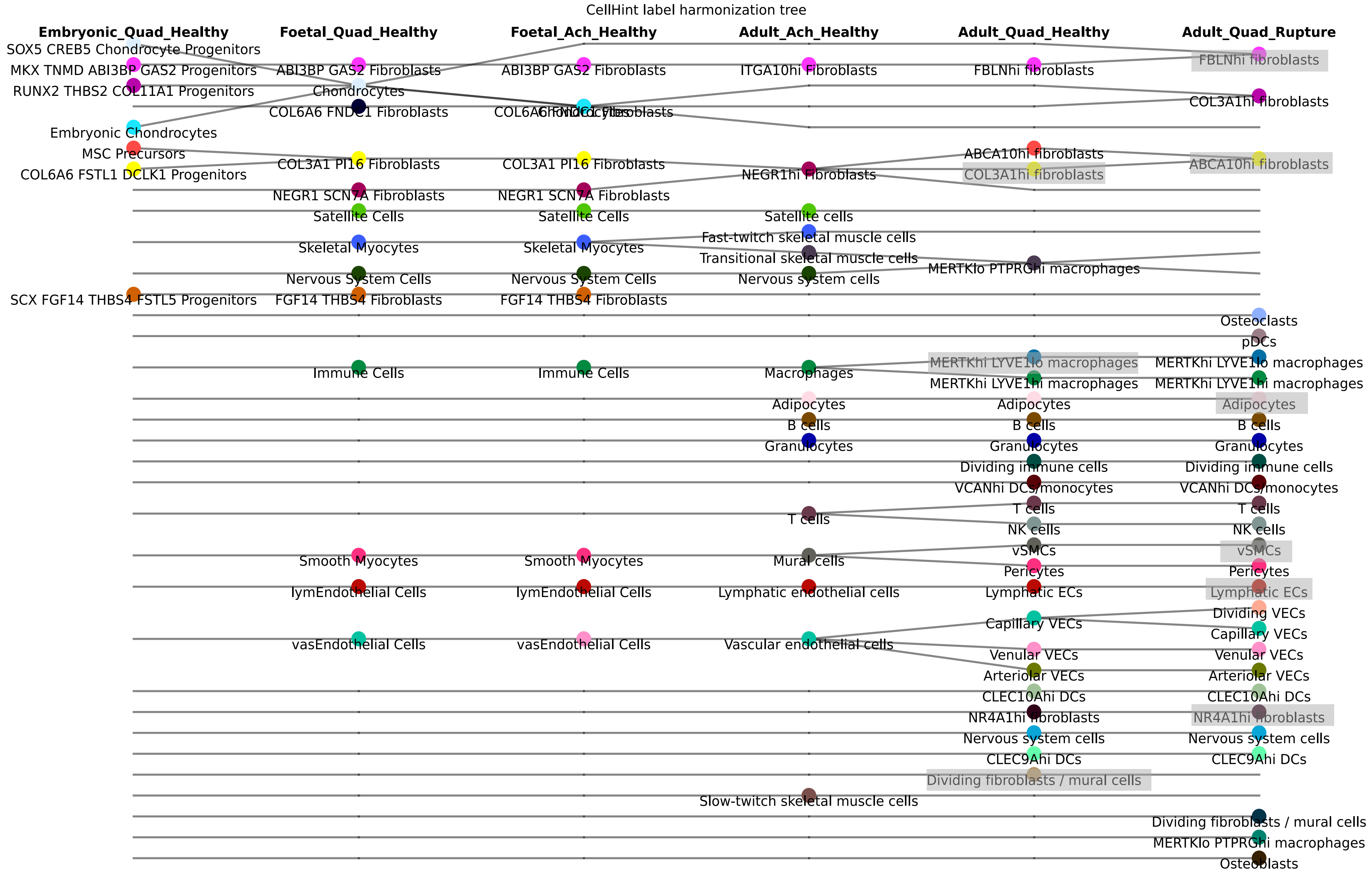
CellHint label harmonization tree
Embryonic_Quad_Healthy
Foetal_Quad_Healthy
Foetal_Ach_Healthy
Adult_Ach_Healthy
Adult_Quad_Healthy
Adult_Quad_Rupture
SOX5 CREB5 Chondrocyte Progenitors
MKX TNMD ABI3BP GAS2 Progenitors
RUNX2 THBS2 COL11A1 Progenitors
Embryonic Chondrocytes
MSC Precursors
COL6A6 FSTL1 DCLK1 Progenitors
SCX FGF14 THBS4 FSTL5 Progenitors
ABI3BP GAS2 Fibroblasts
Chondrocytes
COL6A6 FNDC1 Fibroblasts
COL3A1 PI16 Fibroblasts
NEGR1 SCN7A Fibroblasts
Satellite Cells
Skeletal Myocytes
Nervous System Cells
FGF14 THBS4 Fibroblasts
Immune Cells
Smooth Myocytes
lymEndothelial Cells
vasEndothelial Cells
ITGA10hi Fibroblasts
NEGR1hi Fibroblasts
Satellite cells
Fast-twitch skeletal muscle cells
Transitional skeletal muscle cells
Nervous system cells
Macrophages
Adipocytes
B cells
Granulocytes
T cells
Mural cells
Lymphatic endothelial cells
Vascular endothelial cells
Slow-twitch skeletal muscle cells
FBLNhi fibroblasts
ABCA10hi fibroblasts
COL3A1hi fibroblasts
MERTKlo PTPRGhi macrophages
MERTKhi LYVE1lo macrophages
MERTKhi LYVE1hi macrophages
Dividing immune cells
VCANhi DCs/monocytes
NK cells
vSMCs
Pericytes
Lymphatic ECs
Capillary VECs
Venular VECs
Arteriolar VECs
CLEC10Ahi DCs
NR4A1hi fibroblasts
CLEC9Ahi DCs
Dividing fibroblasts / mural cells
Osteoclasts
pDCs
Dividing VECs
Osteoblasts

Figure S11. CellHint harmonisation of annotated cell types across datasets and conditions.

Tree plot shows transcriptional relationships between matched cell types, based on distances calculated from batch-corrected scVI embeddings. Greyed out cell types represent unusually low cell populations (less than 102 cells) within a given dataset.

Adult Ruptured COL3A1hi Fibroblasts vs Foetal COL6A6 FNDC1 Fibroblasts

A

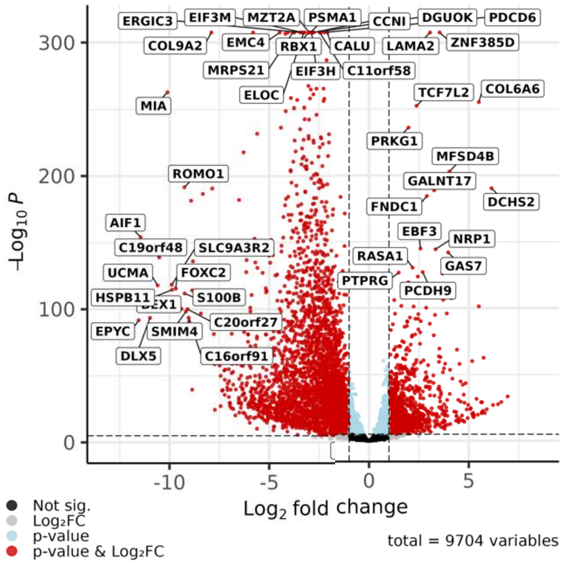

Downregulated BH FDR <0.01 (571):	Upregulated BH FDR <0.01 (325):
- Energy Metabolism and Mitochondrial Function<br>- Nucleotide and Ribose Phosphate Mechanism<br>- Protein Metabolism and Folding<br>- RNA Metabolism<br>- Protein Translation<br>- Cell Cycle and Division<br>- Response to Stress and Apoptosis<br>- Autophagy and Senescence<br>- Responses to TGF-b Signalling	- Cell Adhesion and Extracellular Matrix Organisation<br>- Muscle Development and Contraction<br>- Calcium Ion Transport and Homeostasis<br>- Neuron and Synapse Development and Organisation<br>- Cell Signalling and Transduction (Wnt signalling, GTPase, Rac, Rho, and GPCR transduction pathways)<br>- Organ/Tissue Developmental Processes and Cell Growth<br>- Vascular Development and Blood Circulation<br>- Cell Migration and Morphogenesis<br>- Immune and Inflammatory Responses

B

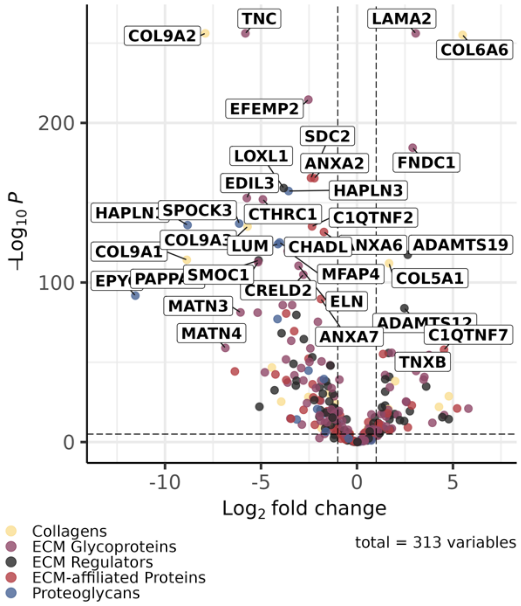

Downregulated padj <0.01, log2FC < -1 (127):	Upregulated padj <0.01, log2FC > 1 (66):
**Collagens:** COL9A1, COL9A2, COL9A3, COL8A1, COL2A1, COL26A1, COL11A2, COL11A1, COL15A1, COL8A2, COL13A1<br>**Proteoglycans:** EPYC, HAPLN1, SPOCK3, PODNL1, LUM, CHADL, PRELP, FMOD, ACAN, OGN, SPOCK1, OMD<br>**Glycoproteins:** MATN4, MATN3, TNC, EDIL3, EFEMP1, SMOC1, CTHRC1, MATN1, MFAP2, PCOLCE2, CILP2, MFAP4, NPNT, NELL2, LTBP1, IGFBP2, ELN, TSKU, FBN3, CRELD2, VWA1, CRISPLD2, EFEMP2, MFGE8, IGFBP5, MFAP1, IGFBP3, GAS6, RSPO3, FBLN2, IGFBP4, COMP, MGP, TGFBI, CRISPLD1, FBLN1, SMOC2, THBS2, EMID1, AEBP1, EMILIN1, SRPX, SPARC<br>**ECM-affiliated Proteins:** LGALS1, SDC4, GPC1, CLEC11A, SEMA3E, SDC1, SDC2, C1QTNF3, GPC5, SEMA5B, C1QTNF2, C1QTNF4, ANXA2, PLXNA4, ANXA5, ANXA7, ANXA6, LGALS3, GREM1, C1QTNF1, C1QTNF6<br>**ECM Regulators:** PAPPA2, CSTB, CST3, TIMP1, LOXL1, HYAL2, SERPINF1, MMP11, CTSD, CTSH, CTSK, SERPINH1, MMP28, EGLN2, CTSA, CTSB, PLOD3, PLOD1, SERPING1, P4HTM, TIMP3, SERPINI1, ADAMTS6, ADAMTS17, MMP2, SERPINE1, CTSC, MMP14, KAZALD1, ADAMTSL1, ADAM15	**Collagens:** COL6A6, COL5A3, COL10A1, COL6A3, COL5A1, COL18A1<br>**Glycoproteins:** HMCN2, EYS, FGL2, TNXB, NTN1, MFAP5, EMILIN2, LAMA2, FNDC1, ECM2, IGSF10, POSTN, NTNG1, DPT, LAMB1, FBN1, LAMA1, TNFAIP6, MATN2, HMCN1, RSPO2, SLIT2, ABI3BP, SPON2, POMZP3<br>**ECM-affiliated Proteins:** FREM3, C1QTNF7, PLXNC1, EMCN, FREM1, SEMA6C, SEMA3C, ELFN1, SEMA6D, SEMA3D, PLXNA3<br>**ECM Regulators:** MMP19, MMP21, ITIH5, ADAM33, ADAMTS19, ADAMTS12, PCSK6, PAPPA, ADAM32, ADAM19, ADAM12, ADAMTS2, ADAMTSL3, ADAM22, SULF2, ADAMTS9, CD109, ADAMTS15, ADAMTS5, SULF1, FAM20A, FAM20C, MMP24, SERPINB1

Figure S12. Analysis of significantly up- and downregulated genes and pathways in adult ruptured COL3A1hi Fibroblasts vs foetal COL6A6 FNDC1 fibroblasts.

(A) Volcano plot illustrating significantly up- and downregulated genes determined by the DESeq2 Wald test (p-adjusted < 0.01, log2FC ±1). The table to the right summarises the results of gene ontology (GO) biological processes (BP) analyses conducted using the gProfiler g:GOSt tool (Raudvere et al., 2019). GO:BP pathways sized 20-500 terms with Benjamini-Hochberg (BH) FDR values <0.01 were analysed and categorised by common functions (e.g. different pathways related to apoptosis were grouped together into an "Apoptosis" category). The numbers in brackets show the total number of different GO:BP pathways meeting the BH FDR criteria. (B) Volcano plot and accompanying table showing significantly up- and downregulated matrisomal genes, identified using the DESeq2 Wald test (p-adjusted < 0.01, log2FC ±1).

Adult Ruptured COL3A1hi Fibroblasts vs Foetal Chondrocytes

A

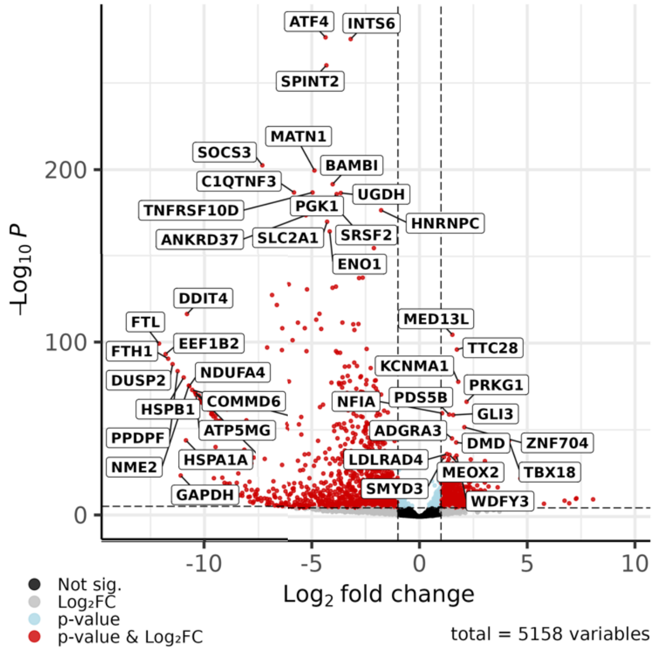

Downregulated BH FDR <0.01 (519):	Upregulated BH FDR <0.01 (126):
- Energy Metabolism and Mitochondrial Function<br>- Nucleotide and Ribose Phosphate Mechanism<br>- Protein Metabolism and Folding<br>- RNA Metabolism<br>- Protein Translation<br>- Cell Cycle and Division<br>- Response to Stress and Apoptosis<br>- Autophagy	- Cell Migration and Adhesion<br>- Cell Morphogenesis<br>- Muscle Cell Development and Differentiation<br>- Neuron and Axon Development<br>- Limb Development and Morphogenesis<br>- Cell Signalling (GTPase, Wnt, TGF-b, Smoothened, TOR and insulin)<br>- Cell Cycle and Division<br>- Metabolism and Energy Production<br>- Protein Modification and Degradation

B

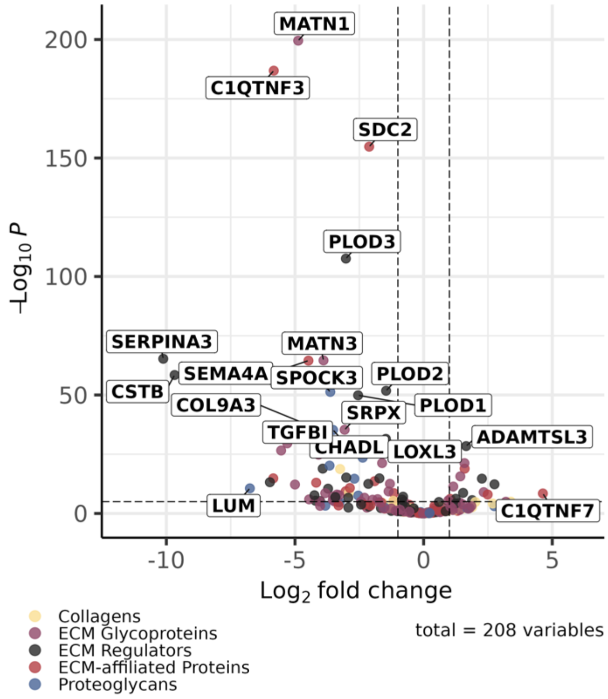

Downregulated padj <0.01, log2FC < -1 (78):	Upregulated padj <0.01, log2FC > 1 (28):
**Collagens:** COL9A3, COL9A2, COL6A2, COL9A1, COL2A1, COL15A1, COL8A1<br>**Proteoglycans:** LUM, EPYC, PRELP, SPOCK3, CHADL, DCN, HAPLN1, CHAD, SPOCK1, FMOD<br>**Glycoproteins:** MATN4, TGFBI, MGP, MATN1, IGFBP4, CTHRC1, EFEMP1, MFAP4, MATN3, VWA1, IGFBP7, PCOLCE, IGFBP2, IGFBP5, EFEMP2, SRPX, FBLN1, MFAP2, COMP, PCOLCE2, GAS6, LAMB2, NID2, AEBP1<br>**ECM-affiliated Proteins:** GPC3, C1QTNF3, SEMA4A, LGALS3, LGALS1, ANXA5, ANXA2, GPC1, PLXNA2, SDC2, SDC1, GPC5, LMAN1, ANXA6, ANXA7<br>**ECM Regulators:** SERPINA3, CSTB, TIMP1, SERPINH1, CTSL, CTSD, LOXL1, EGLN3, SERPINE1, PLOD3, P4HA2, SERPINE2, CST3, PLOD1, TIMP3, LOXL2, MMP2, P4HA1, CTSB, LOXL3, PLOD2	**Collagens:** COL1A1, COL5A2, COL24A1, COL12A1, COL25A1<br>**Proteoglycans:** ASPN<br>**Glycoproteins:** ECM2, FNDC1, LAMA2, SPON1, LTBP2, THSD4, SLIT3, SMOC1, CRISPLD1, SVEP1, TNC<br>**ECM-affiliated Proteins:** C1QTNF7, SEMA6D, PLXDC2, COLEC12<br>**ECM Regulators:** CD109, ADAM12, ADAM22, ADAMTSL3, ADAMTS19, FAM20C, MMP16

Figure S13. Analysis of significantly up- and downregulated genes and pathways in adult ruptured COL3A1hi Fibroblasts vs foetal Chondrocytes.

(A) Volcano plot illustrating significantly up- and downregulated genes determined by the DESeq2 Wald test (p-adjusted < 0.01, log2FC ±1). The table to the right summarises the results of gene ontology (GO) biological processes (BP) analyses conducted using the gProfiler g:GOSt tool (Raudvere et al., 2019). GO:BP pathways sized 20-500 terms with Benjamini-Hochberg (BH) FDR values <0.01 were analysed and categorised by common functions (e.g. different pathways related to apoptosis were grouped together into an "Apoptosis" category). The numbers in brackets show the total number of different GO:BP pathways meeting the BH FDR criteria. (B) Volcano plot and accompanying table showing significantly up- and downregulated matrisomal genes, identified using the DESeq2 Wald test (p-adjusted < 0.01, log2FC ±1).

Adult Healthy FBLNhi Fibroblasts vs Foetal ABI3BP GAS2 Fibroblasts

A

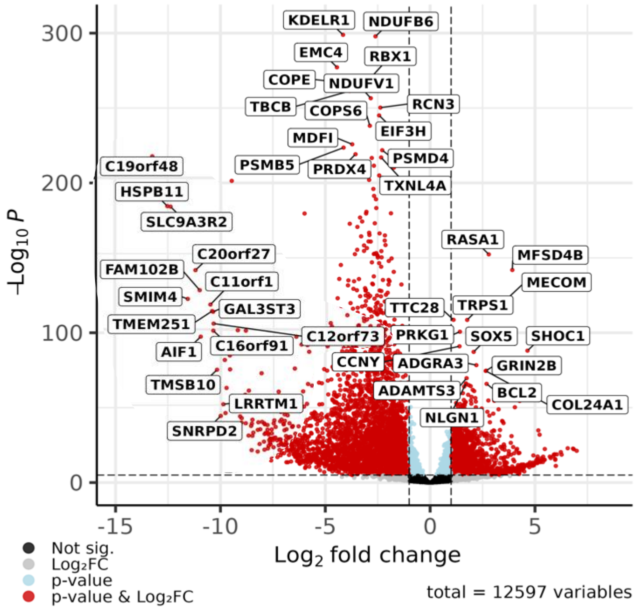

Downregulated BH FDR <0.01 (573):	Upregulated BH FDR <0.01 (141):
- Energy Metabolism and Mitochondrial Function<br>- Nucleotide and Ribose Phosphate Mechanism<br>- Protein Metabolism and Folding<br>- RNA Metabolism<br>- Protein Translation<br>- Proteolysis, Protein Catabolism<br>- Cell Cycle and Division<br>- Response to Stress and Apoptosis	- Cell Migration and Adhesion<br>- ECM Organisation<br>- Cell Morphogenesis<br>- Neuron and Axon Development<br>- Limb Development and Morphogenesis<br>- Cell Signalling (GTPase, Wnt, TGF-b, Smoothened, P3PKB)<br>- Ion Transport<br>- Protein and Lipid Metabolism

B

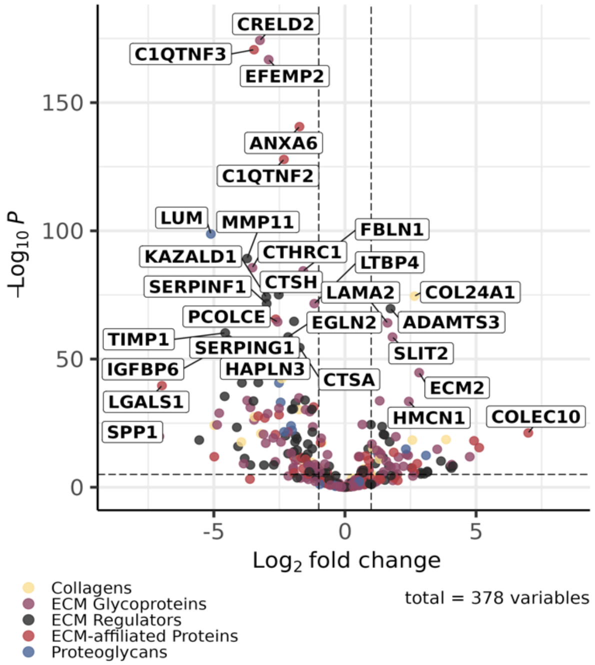

Downregulated padj <0.01, log2FC < -1 (124):	Upregulated padj <0.01, log2FC > 1 (74):
**Collagens:** COL9A1, COL22A1, COL9A3, COL4A1, COL3A1, COL26A1, COL6A2, COL4A2, COL2A1, COL25A1<br>**Proteoglycans:** LUM, HAPLN3, ASPN, DCN, SPOCK1, BGN, OGN, KERA, CHADL<br>**Glycoproteins:** SPP1, IGFBP2, IGFBP4, IGFBP6, EFEMP1, MFAP4, MFAP2, CTHRC1, BGLAP, CRELD2, MGP, TSKU, VWA1, EFEMP2, RSPO3, FBN3, PCOLCE, TNFAIP6, IGFBP5, SPARCL1, LAMC3, VWCE, NTNG2, IGFBP3, NTNG1, FBLN2, MFAP1, SPON2, NTN4, FBLN1, EMILIN1, IGFBP7, THBS4, LGI4, MFGE8, NELL2, FBLN5, LTBP4, SPARC, TNC, TGFBI<br>**ECM-affiliated Proteins:** LGALS1, CLEC14A, ANXA8, C1QTNF3, GPC5, GPC3, ANXA2, ANXA1, C1QTNF2, SDC4, GPC1, CLEC11A, SEMA4A, SEMA3A, SEMA7A, ANXA5, ANXA6, ANXA7, C1QL1<br>**ECM Regulators:** CSTB, CST3, TIMP1, SERPING1, AGT, C17orf58, HYAL2, A2M, MMP11, CTSC, TLL1, SERPINE2, F13A1, KAZALD1, SERPINF1, PLAT, CTSH, CTSK, CTSL, CTSZ, LOXL1, ADAM15, CTSF, HTRA3, MMP2, SERPINE1, CTSA, CTSD, P4HTM, P4HA2, MMP14, MMP23B, EGLN2, EGLN3, TIMP3, SERPINB6, PLOD1, PLOD3, MMP15, OGFOD2	**Collagens:** COL10A1, COL24A1, COL6A6, COL4A3, COL6A3, COL5A3, COL8A1, COL27A1<br>**Proteoglycans:** IMPG2, OMD, ACAN<br>**Glycoproteins:** EYS, MMRN2, SNED1, ECM2, TSPEAR, HMCN1, NDNF, GLDN, SMOC1, NTN1, LGI2, RSPO2, MATN2, FGL2, SLIT2, COMP, LAMA2, SPON1, POMZP3, LAMA1, CRIM1, CILP2, ABI3BP, LAMA3, CRISPLD2, SVEP1<br>**ECM-affiliated Proteins:** COLEC10, FREM3, C1QTNF7, PLXNC1, MUC20, SEMA4G, EMCN, MUC4, MUC1, SEMA3D, PLXNB1, SFTPD, CLEC4A<br>**ECM Regulators:** SERPIND1, MASP2, MMP19, MMP21, CELA2B, ADAM21, ADAMTS18, A2ML1, MEP1B, ADAMTS13, HPSE2, PAPPA, ADAMTS3, CD109, ADAM32, HYAL1, ADAMTS19, ADAMTS17, ADAMTS14, ADAM22, ADAMTS6, LOXL3, ADAM12, ADAMTSL2

Figure S14. Analysis of significantly up- and downregulated genes and pathways in adult healthy FBLNhi Fibroblasts vs foetal ABI3BP GAS2 fibroblasts.

(A) Volcano plot illustrating significantly up- and downregulated genes determined by the DESeq2 Wald test (p-adjusted < 0.01, log2FC ±1). The table to the right summarises the results of gene ontology (GO) biological processes (BP) analyses conducted using the gProfiler g:GOSt tool (Raudvere et al., 2019). GO:BP pathways sized 20-500 terms with Benjamini-Hochberg (BH) FDR values <0.01 were analysed and categorised by common functions (e.g. different pathways related to apoptosis were grouped together into an "Apoptosis" category). The numbers in brackets show the total number of different GO:BP pathways meeting the BH FDR criteria. (B) Volcano plot and accompanying table showing significantly up- and downregulated matrisomal genes, identified using the DESeq2 Wald test (p-adjusted < 0.01, log2FC ±1).

Adult Healthy ABCA10hi Fibroblasts vs Foetal COL3A1 PI16 Fibroblasts

A

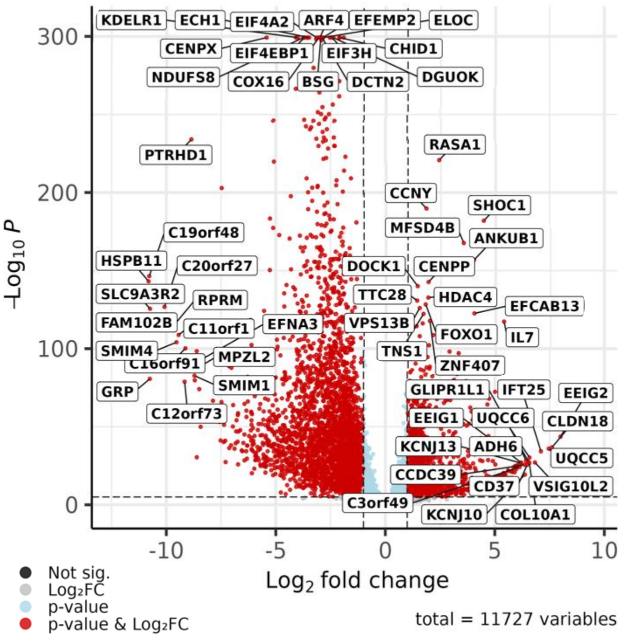

Downregulated BH FDR <0.01 (482):	Upregulated BH FDR <0.01 (186):
- Energy Metabolism and Mitochondrial Function<br>- Nucleotide and Ribose Phosphate Mechanism<br>- Protein Metabolism and Folding<br>- RNA Metabolism<br>- Protein Translation<br>- Proteolysis, Protein Catabolism<br>- Cell Cycle and Division<br>- Response to Stress and Apoptosis<br>- "Epithelial-to-mesenchymal transition"<br>- "Stem cell population maintenance"	- Cell Migration and Adhesion<br>- ECM Organisation<br>- Cell Morphogenesis<br>- Neuron and Axon Development<br>- Limb Development and Morphogenesis<br>- Cell Signalling (GTPase, Wnt, TGF-b, Smoothened, P3PKB)<br>- Ion Transport<br>- Protein and Lipid Metabolism

B

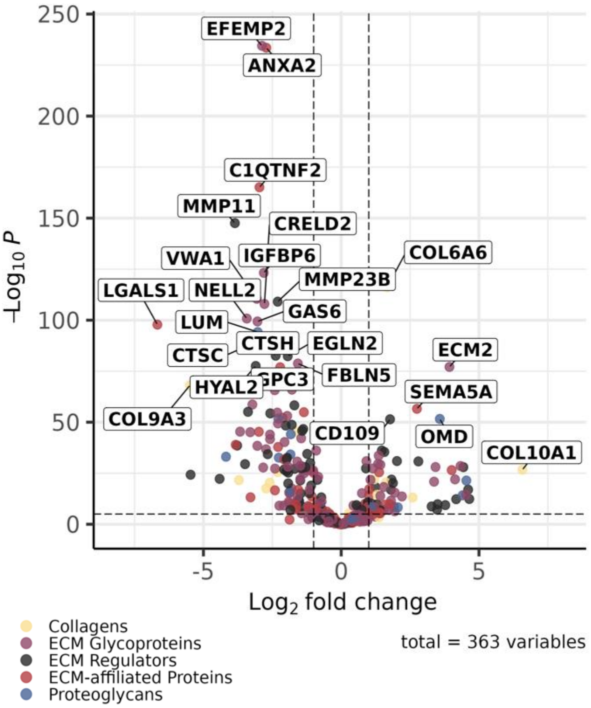

Downregulated padj <0.01, log2FC < -1 (124):	Upregulated padj <0.01, log2FC > 1 (74):
**Collagens:** COL9A3, COL9A1, COL9A2, COL26A1, COL13A1, COL6A2, COL2A1, COL1A1<br>**Proteoglycans:** SPOCK3, LUM, ASPN, FMOD, HAPLN3, DCN, OGN<br>**Glycoproteins:** BGLAP, NELL2, MFAP2, MFAP4, VWA1, CTHRC1, GAS6, EFEMP2, MGP, IGFBP6, CRELD2, RELN, VWCE, PCOLCE, TGFBI, IGFBP3, TSKU, FBN3, MATN4, FBLN1, SPARC, SRPX, IGFBP2, FRAS1, EFEMP1, AEBP1, COCH, FBLN5, VWA5A, TNFAIP6, EMILIN1, MFAP1, EDIL3, IGFBP5, FBLN2, NDNF, MFGE8, PXDNL, LGI4, SPON2, NID2<br>**ECM-affiliated Proteins:** LGALS1, ANXA3, ANXA2, ANXA5, GPC3, ANXA1, CLEC2B, CLEC14A, SDC1, SDC4, SEMA3A, SEMA4A, PARM1, C1QTNF2, C1QTNF3, C1QTNF4, C1QTNF6, ANXA6, ANXA8, LMAN1, GPC1, SDC3, SEMA4F, SEMA3D, SEMA6D, PLXNA3<br>**ECM Regulators:** MASP1, CSTB, MMP11, AGT, CST3, CTSC, TIMP1, CTSD, CTSH, SERPINF1, CTSD, CTSF, LOXL1, SERPINE1, P4HTM, HPSE2, EGLN2, PAPPA2, MMP23B, MMP17, MMP2, ADAMTS1, ADAMTSL2, ADAM15, ADAMTSL3, PLOD1, PLOD3, P4HTM, SERPINB6, SERPING1, LOX, F10, C17orf58	**Collagens:** COL10A1, COL4A3, COL6A6, COL5A3, COL28A1, COL4A1, COL6A3<br>**Proteoglycans:** PRG4, CHAD, OMD, IMPG2, PODN<br>**Glycoproteins:** MMRN2, EYS, NTN4, ECM2, SNED1, VIT, TNN, POMZP3, HMCN2, GLDN, PCOLCE2, EGFLAM, SPON1, LAMA1, MATN2, HMCN1, FBN1, RSPO2, LAMA4, IGFBP7, CRIM1, NTNG1<br>**ECM-affiliated Proteins:** FREM3, SEMA5A, EMCN, PLXDC1, PLXNA2, GPC6, MUC20, CLEC3B, PLXND1, CLEC2L, SEMA5B, SEMA3C, CLEC2D, C1QTNF7<br>**ECM Regulators:** MASP2, MMP21, ADAMTS16, ADAM21, ADAMTS18, A2M, SERPINE3, MEP1B, ADAMTS14, ADAM32, ADAM19, CD109, TLL1, ADAMTS3, ADAMTSL3, ADAMTS19, MMP19, ADAMTS15, ADAM12, MMP24, EGLN3, MMP16, LOXL3

Figure S15. Analysis of significantly up- and downregulated genes and pathways in adult healthy ABCA10 fibroblasts vs foetal COL3A1 PI16 fibroblasts.

(A) Volcano plot illustrating significantly up- and downregulated genes determined by the DESeq2 Wald test (p-adjusted < 0.01, log2FC ±1). The table to the right summarises the results of gene ontology (GO) biological processes (BP) analyses conducted using the gProfiler g:GOSt tool (Raudvere et al., 2019). GO:BP pathways sized 20-500 terms with Benjamini-Hochberg (BH) FDR values <0.01 were analysed and categorised by common functions (e.g. different pathways related to apoptosis were grouped together into an “Apoptosis” category). The numbers in brackets show the total number of different GO:BP pathways meeting the BH FDR criteria. (B) Volcano plot and accompanying table showing significantly up- and downregulated matrisomal genes, identified using the DESeq2 Wald test (p-adjusted < 0.01, log2FC ±1).

A

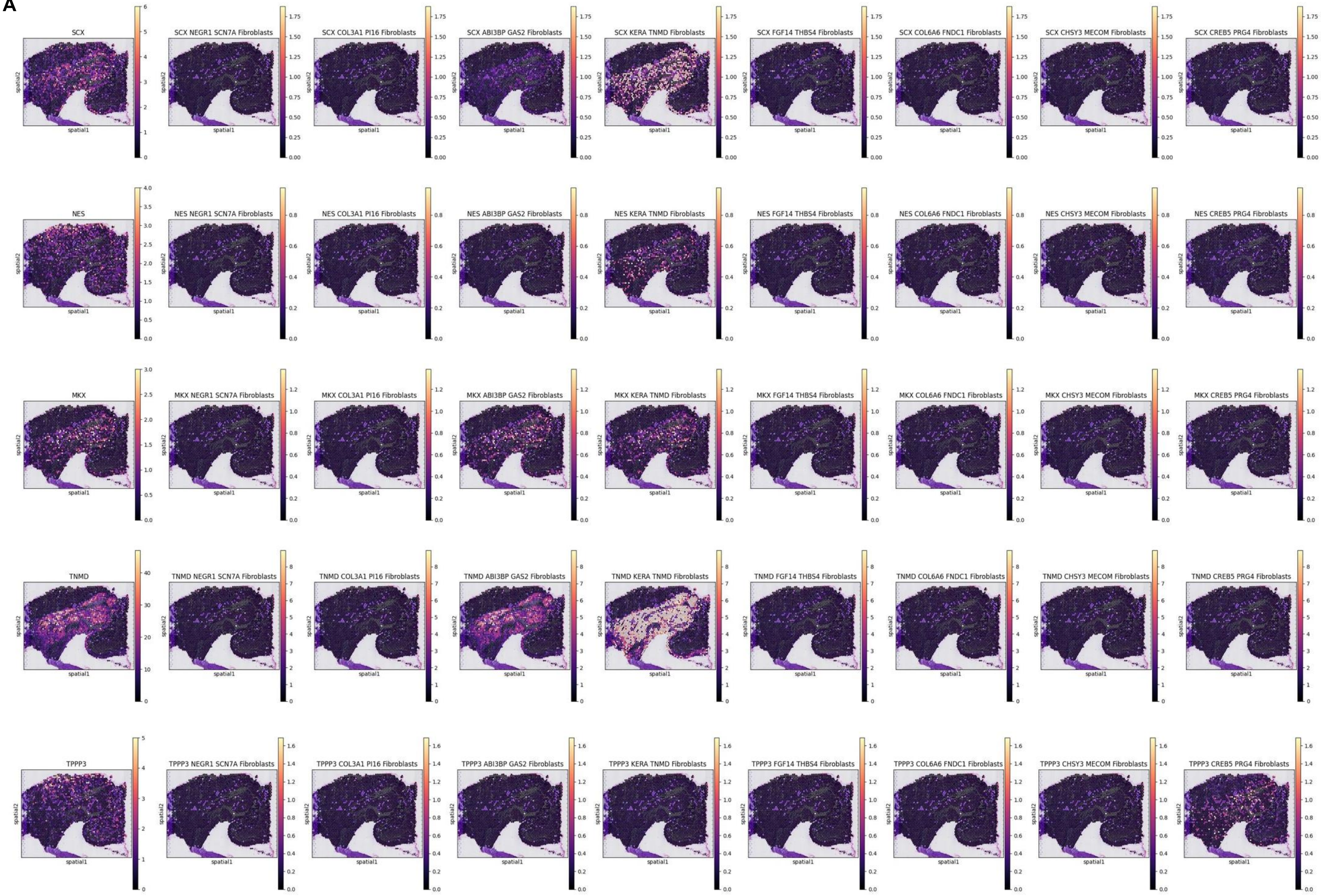

SCX
SCX NEGR1 SCN7A Fibroblasts
SCX COL3A1 PI16 Fibroblasts
SCX ABI3BP GAS2 Fibroblasts
SCX KERA TNMD Fibroblasts
SCX FGF14 THBS4 Fibroblasts
SCX COL6A6 FNDC1 Fibroblasts
SCX CHSY3 MECOM Fibroblasts
SCX CREB5 PRG4 Fibroblasts
NES
NES NEGR1 SCN7A Fibroblasts
NES COL3A1 PI16 Fibroblasts
NES ABI3BP GAS2 Fibroblasts
NES KERA TNMD Fibroblasts
NES FGF14 THBS4 Fibroblasts
NES COL6A6 FNDC1 Fibroblasts
NES CHSY3 MECOM Fibroblasts
NES CREB5 PRG4 Fibroblasts
MKX
MKX NEGR1 SCN7A Fibroblasts
MKX COL3A1 PI16 Fibroblasts
MKX ABI3BP GAS2 Fibroblasts
MKX KERA TNMD Fibroblasts
MKX FGF14 THBS4 Fibroblasts
MKX COL6A6 FNDC1 Fibroblasts
MKX CHSY3 MECOM Fibroblasts
MKX CREB5 PRG4 Fibroblasts
TNMD
TNMD NEGR1 SCN7A Fibroblasts
TNMD COL3A1 PI16 Fibroblasts
TNMD ABI3BP GAS2 Fibroblasts
TNMD KERA TNMD Fibroblasts
TNMD FGF14 THBS4 Fibroblasts
TNMD COL6A6 FNDC1 Fibroblasts
TNMD CHSY3 MECOM Fibroblasts
TNMD CREB5 PRG4 Fibroblasts
TPPP3
TPPP3 NEGR1 SCN7A Fibroblasts
TPPP3 COL3A1 PI16 Fibroblasts
TPPP3 ABI3BP GAS2 Fibroblasts
TPPP3 KERA TNMD Fibroblasts
TPPP3 FGF14 THBS4 Fibroblasts
TPPP3 COL6A6 FNDC1 Fibroblasts
TPPP3 CHSY3 MECOM Fibroblasts
TPPP3 CREB5 PRG4 Fibroblasts
spatial1
spatial2

B

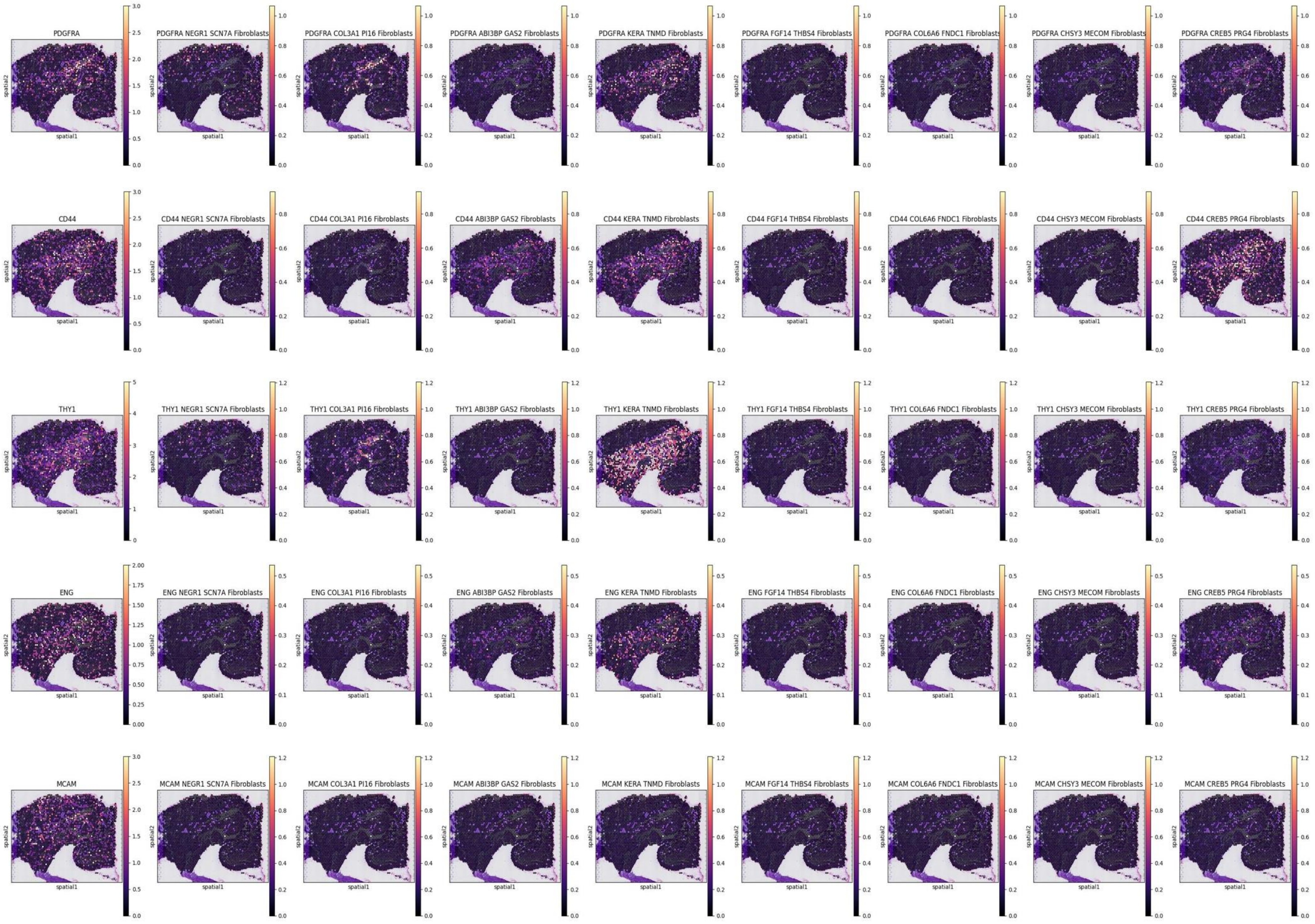
PDGFRA
PDGFRA NEGR1 SCN7A Fibroblasts
PDGFRA COL3A1 PI16 Fibroblasts
PDGFRA ABI3BP GAS2 Fibroblasts
PDGFRA KERA TNMD Fibroblasts
PDGFRA FGF14 THBS4 Fibroblasts
PDGFRA COL6A6 FNDC1 Fibroblasts
PDGFRA CHSY3 MECOM Fibroblasts
PDGFRA CREB5 PRG4 Fibroblasts
CD44
CD44 NEGR1 SCN7A Fibroblasts
CD44 COL3A1 PI16 Fibroblasts
CD44 ABI3BP GAS2 Fibroblasts
CD44 KERA TNMD Fibroblasts
CD44 FGF14 THBS4 Fibroblasts
CD44 COL6A6 FNDC1 Fibroblasts
CD44 CHSY3 MECOM Fibroblasts
CD44 CREB5 PRG4 Fibroblasts
THY1
THY1 NEGR1 SCN7A Fibroblasts
THY1 COL3A1 PI16 Fibroblasts
THY1 ABI3BP GAS2 Fibroblasts
THY1 KERA TNMD Fibroblasts
THY1 FGF14 THBS4 Fibroblasts
THY1 COL6A6 FNDC1 Fibroblasts
THY1 CHSY3 MECOM Fibroblasts
THY1 CREB5 PRG4 Fibroblasts
ENG
ENG NEGR1 SCN7A Fibroblasts
ENG COL3A1 PI16 Fibroblasts
ENG ABI3BP GAS2 Fibroblasts
ENG KERA TNMD Fibroblasts
ENG FGF14 THBS4 Fibroblasts
ENG COL6A6 FNDC1 Fibroblasts
ENG CHSY3 MECOM Fibroblasts
ENG CREB5 PRG4 Fibroblasts
MCAM
MCAM NEGR1 SCN7A Fibroblasts
MCAM COL3A1 PI16 Fibroblasts
MCAM ABI3BP GAS2 Fibroblasts
MCAM KERA TNMD Fibroblasts
MCAM FGF14 THBS4 Fibroblasts
MCAM COL6A6 FNDC1 Fibroblasts
MCAM CHSY3 MECOM Fibroblasts
MCAM CREB5 PRG4 Fibroblasts

Data S1. Fibroblast type-specific expression of fascicular and sheath TSPC markers.

(A, B) Visium spatial RNA-seq plots show normalised gene expression scores of selected markers across all cells (left column) and within select cell types (all other columns).

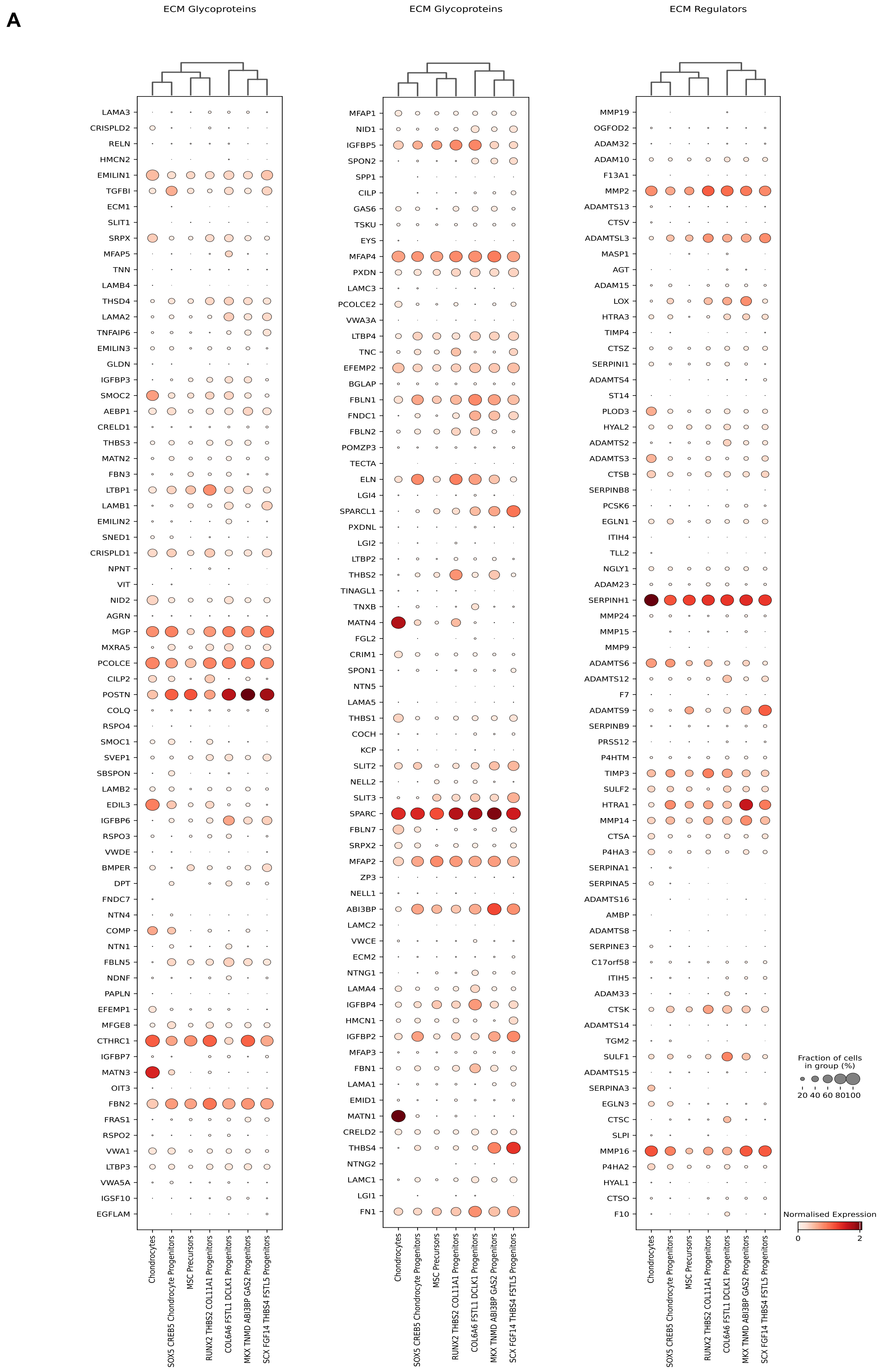
A
ECM Glycoproteins
LAMA3 CRISPLD2 RELN HMCN2 EMILIN1 TGFBI ECM1 SLIT1 SRPX MFAP5 TNN LAMB4 THSD4 LAMA2 TNFAIP6 EMILIN3 GLDN IGFBP3 SMOC2 AEBP1 CRELD1 THBS3 MATN2 FBN3 LTBP1 LAMB1 EMILIN2 SNED1 CRISPLD1 NPNT VIT NID2 AGRN MGP MXRA5 PCOLCE CILP2 POSTN COLQ RSPO4 SMOC1 SVEP1 SBSPON LAMB2 EDIL3 IGFBP6 RSPO3 VWDE BMPER DPT FNDC7 NTN4 COMP NTN1 FBLN5 NDNF PAPLN EFEMP1 MFGE8 CTHRC1 IGFBP7 MATN3 OIT3 FBN2 FRAS1 RSPO2 VWA1 LTBP3 VWA5A IGSF10 EGFLAM
Chondrocytes
SOX5 CREB5 Chondrocyte Progenitors
MSC Precursors
RUNX2 THBS2 COL11A1 Progenitors
COL6A6 FSTL1 DCLK1 Progenitors
MKX TNMD ABI3BP GAS2 Progenitors
SCX FGF14 THBS4 FSTL5 Progenitors
ECM Glycoproteins
MFAP1 NID1 IGFBP5 SPON2 SPP1 CILP GAS6 TSKU EYS MFAP4 PXDN LAMC3 PCOLCE2 VWA3A LTBP4 TNC EFEMP2 BGLAP FBLN1 FNDC1 FBLN2 POMZP3 TECTA ELN LGI4 SPARCL1 PXDNL LGI2 LTBP2 THBS2 TINAGL1 TNXB MATN4 FGL2 CRIM1 SPON1 NTN5 LAMA5 THBS1 COCH KCP SLIT2 NELL2 SLIT3 SPARC FBLN7 SRPX2 MFAP2 ZP3 NELL1 ABI3BP LAMC2 VWCE ECM2 NTNG1 LAMA4 IGFBP4 HMCN1 IGFBP2 MFAP3 FBN1 LAMA1 EMID1 MATN1 CRELD2 THBS4 NTNG2 LAMC1 LGI1 FN1
Chondrocytes
SOX5 CREB5 Chondrocyte Progenitors
MSC Precursors
RUNX2 THBS2 COL11A1 Progenitors
COL6A6 FSTL1 DCLK1 Progenitors
MKX TNMD ABI3BP GAS2 Progenitors
SCX FGF14 THBS4 FSTL5 Progenitors
ECM Regulators
MMP19 OGFOD2 ADAM32 ADAM10 F13A1 MMP2 ADAMTS13 CTSV ADAMTSL3 MASP1 AGT ADAM15 LOX HTRA3 TIMP4 CTSZ SERPINI1 ADAMTS4 ST14 PLOD3 HYAL2 ADAMTS2 ADAMTS3 CTSB SERPINB8 PCSK6 EGLN1 ITIH4 TLL2 NGLY1 ADAM23 SERPINH1 MMP24 MMP15 MMP9 ADAMTS6 ADAMTS12 F7 ADAMTS9 SERPINB9 PRSS12 P4HTM TIMP3 SULF2 HTRA1 MMP14 CTSA P4HA3 SERPINA1 SERPINA5 ADAMTS16 AMBP ADAMTS8 SERPINE3 C17orf58 ITIH5 ADAM33 CTSK ADAMTS14 TGM2 SULF1 ADAMTS15 SERPINA3 EGLN3 CTSC SLPI MMP16 P4HA2 HYAL1 CTSO F10
Chondrocytes
SOX5 CREB5 Chondrocyte Progenitors
MSC Precursors
RUNX2 THBS2 COL11A1 Progenitors
COL6A6 FSTL1 DCLK1 Progenitors
MKX TNMD ABI3BP GAS2 Progenitors
SCX FGF14 THBS4 FSTL5 Progenitors
Fraction of cells in group (%)
20 40 60 80100
Normalised Expression
0 2

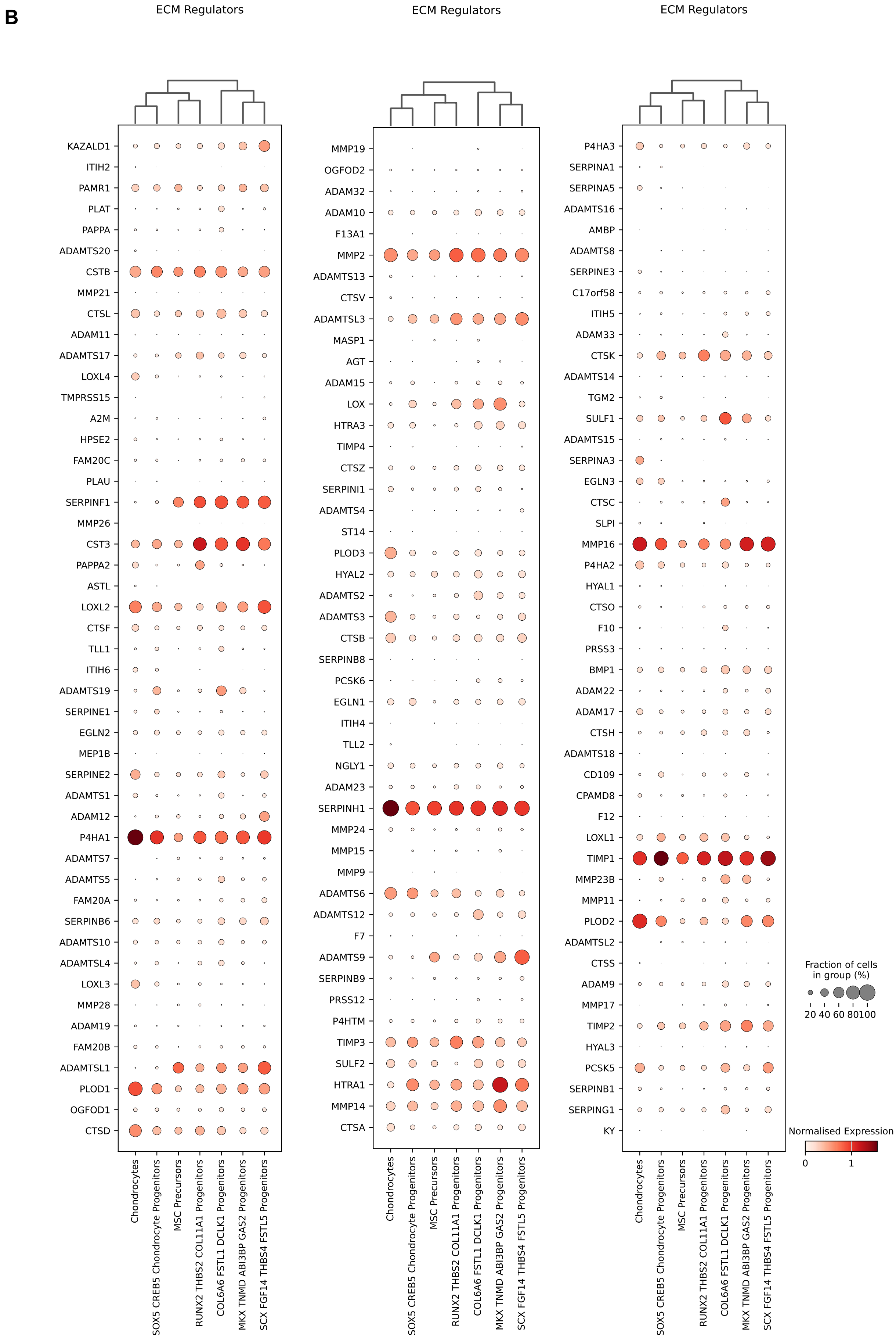
B
ECM Regulators
KAZALD1
ITIH2
PAMR1
PLAT
PAPPA
ADAMTS20
CSTB
MMP21
CTSL
ADAM11
ADAMTS17
LOXL4
TMPRSS15
A2M
HPSE2
FAM20C
PLAU
SERPINF1
MMP26
CST3
PAPPA2
ASTL
LOXL2
CTSF
TLL1
ITIH6
ADAMTS19
SERPINE1
EGLN2
MEP1B
SERPINE2
ADAMTS1
ADAM12
P4HA1
ADAMTS7
ADAMTS5
FAM20A
SERPINB6
ADAMTS10
ADAMTSL4
LOXL3
MMP28
ADAM19
FAM20B
ADAMTSL1
PLOD1
OGFOD1
CTSD
ECM Regulators
MMP19
OGFOD2
ADAM32
ADAM10
F13A1
MMP2
ADAMTS13
CTSV
ADAMTSL3
MASP1
AGT
ADAM15
LOX
HTRA3
TIMP4
CTSZ
SERPINI1
ADAMTS4
ST14
PLOD3
HYAL2
ADAMTS2
ADAMTS3
CTSB
SERPINB8
PCSK6
EGLN1
ITIH4
TLL2
NGLY1
ADAM23
SERPINH1
MMP24
MMP15
MMP9
ADAMTS6
ADAMTS12
F7
ADAMTS9
SERPINB9
PRSS12
P4HTM
TIMP3
SULF2
HTRA1
MMP14
CTSA
ECM Regulators
P4HA3
SERPINA1
SERPINA5
ADAMTS16
AMBP
ADAMTS8
SERPINE3
C17orf58
ITIH5
ADAM33
CTSK
ADAMTS14
TGM2
SULF1
ADAMTS15
SERPINA3
EGLN3
CTSC
SLPI
MMP16
P4HA2
HYAL1
CTSO
F10
PRSS3
BMP1
ADAM22
ADAM17
CTSH
ADAMTS18
CD109
CPAMD8
F12
LOXL1
TIMP1
MMP23B
MMP11
PLOD2
ADAMTSL2
CTSS
ADAM9
MMP17
TIMP2
HYAL3
PCSK5
SERPINB1
SERPING1
KY
Chondrocytes
SOX5 CREB5 Chondrocyte Progenitors
MSC Precursors
RUNX2 THBS2 COL11A1 Progenitors
COL6A6 FSTL1 DCLK1 Progenitors
MKX TNMD ABI3BP GAS2 Progenitors
SCX FGF14 THBS4 FSTL5 Progenitors
Fraction of cells
in group (%)
20 40 60 80100
Normalised Expression
0
1

C

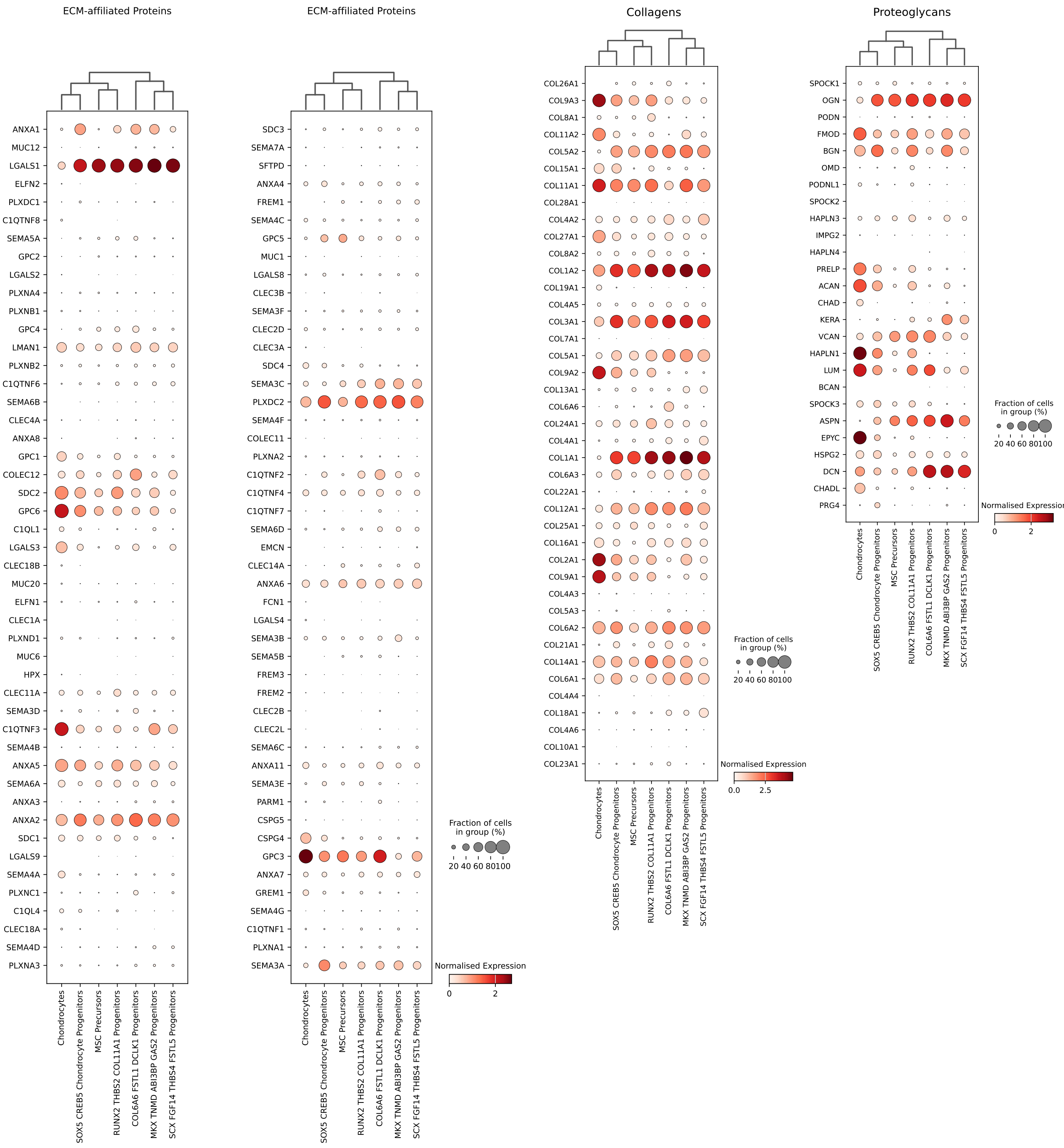

Data S2. Embryonic 6-9pcw tendon matrisomal gene expression profiles.

(A-C) Dotplots show mean normalised expression levels alongside the proportions of expressing cell types for matrisomal genes categorised as ECM-affiliated proteins, ECM glycoproteins, and ECM regulators. The lists of matrisomal genes were obtained from the matrisomeDB database (Shao et al., 2020).
